# Dual reactivity disulfide bridging reagents; enabling new approaches to antibody fragment bioconjugation†

**DOI:** 10.1039/d2sc04531a

**Published:** 2022-09-27

**Authors:** Alina Chrzastek, Ioanna A. Thanasi, James A. Irving, Vijay Chudasama, James R. Baker

**Affiliations:** Department of Chemistry, University College London 20 Gordon Street WC1H OAJ London UK j.r.baker@ucl.ac.uk v.chudasama@ucl.ac.uk; UCL Respiratory, Rayne Institute, University College London WC1E 6JF London UK

## Abstract

Disulfide bridging, also known as disulfide stapling, is a powerful strategy for the construction of site-selective protein bioconjugates. Here we describe the first examples of a new class of such reagents, containing a ‘stable-labile’ design. These dual-reactive reagents are designed to form a stable bond to one cysteine and a labile bond to the second; resulting in a robust attachment to the protein with one end of the bridge, whilst the other end serves as a reactive handle for subsequent bioconjugation. By incorporating thioesters into these bridges, we demonstrate that they are primed for native chemical ligation (NCL) with N-terminal cysteines; offering an alternative to the requirement for C-terminal thioesters for use in such ligations. Alternatively, the use of hydrazine as the ligating nucleophile enables a separate cargo to be attached to each cysteine residue, which are exploited to insert variably cleavable linkers. These methodologies are demonstrated on an antibody fragment, and serve to expand the scope of disulfide bridging strategies whilst offering a convenient route to the construction of multifunctional antibody fragment conjugates.

## Abstract 2

Here we describe the first examples of a new class of disulfide bridging reagents, designed to insert a ‘stable-labile’ linkage; which can then be exploited to generate dual functional antibody fragment conjugates.

## Graphical abstract

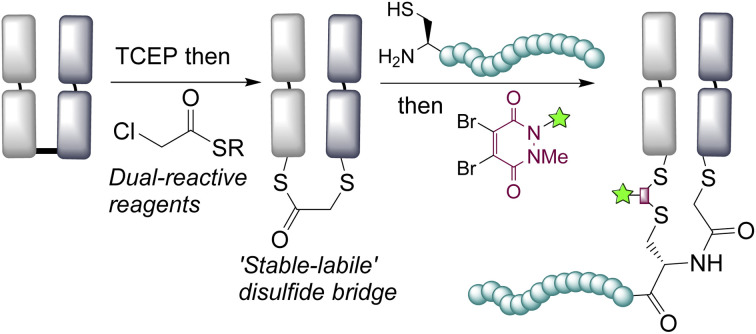

## Introduction

Disulfide bridging, also known as disulfide stapling, is a powerful strategy for the construction of site-selective bioconjugates. Many proteins and peptides contain accessible disulfide bonds, which can be targeted by reduction-bridging protocols to afford conjugates which retain the covalent link between the cysteine residues. For example, this represents a leading approach for the construction of site-selective antibody–drug conjugates (ADCs) from native antibodies.^1,2^ Fab antibody fragments, which contain a single accessible interchain disulfide bond, have been very effectively targeted using such strategies for the construction of ADCs, imaging agents, bispecifics, and multifunctional conjugates.^3–6^ A range of reagents have been developed to effect this bridging of disulfide bonds,^7^ including next generation maleimides (NGMs),^8–10^ pyridazinediones (PDs),^11,12^ bis-sulfones,^3^ divinylpyrimidines,^13,14^ divinyltriazines,^15,16^ arylenedipropiolonitriles,^17^ dichlorotetrazines,^18^ and others.^19–22^ Whilst such reagents are valuable additions to the bioconjugation toolbox they often require multistep syntheses to access functionalised variants, and new strategies which expand the applications afforded by disulfide bridging and offer facile access to multifunctional conjugates,^23^ are widely sought. In one recent example of the diversification of conjugates accessible by disulfide bridging, we have described bisthioester reagents, which following bridging subsequently undergo cysteine-to-lysine transfer to afford site-selective lysine conjugates.^24^

**Scheme 1:**
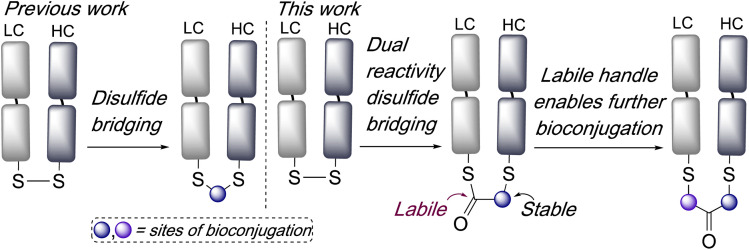
Previous work has involved the development of reagents which re-bridge disulfide bonds. In this work, reagents which have dual reactivity are explored, which incorporate a labile bond into a bridge and serves as a handle to enable subsequent bioconjugation.

Here we envisaged that an intriguing alternative for disulfide bridging reagents would be represented by a dual-reactivity ‘stable-labile’ design *i.e.* containing a functional group which reacts to form a stable bond with one cysteine, and another functional group which reacts to form a labile bond with the other cysteine. This would result in a robust attachment to the protein with one end of the bridge, whilst the other serves as a reactive handle for subsequent bioconjugation (Scheme 1). Furthermore, use of a thioester as the labile component would represent an opportunity for native chemical ligation (NCL) mediated functionalisation.

## Results and discussion

**Scheme 2:**
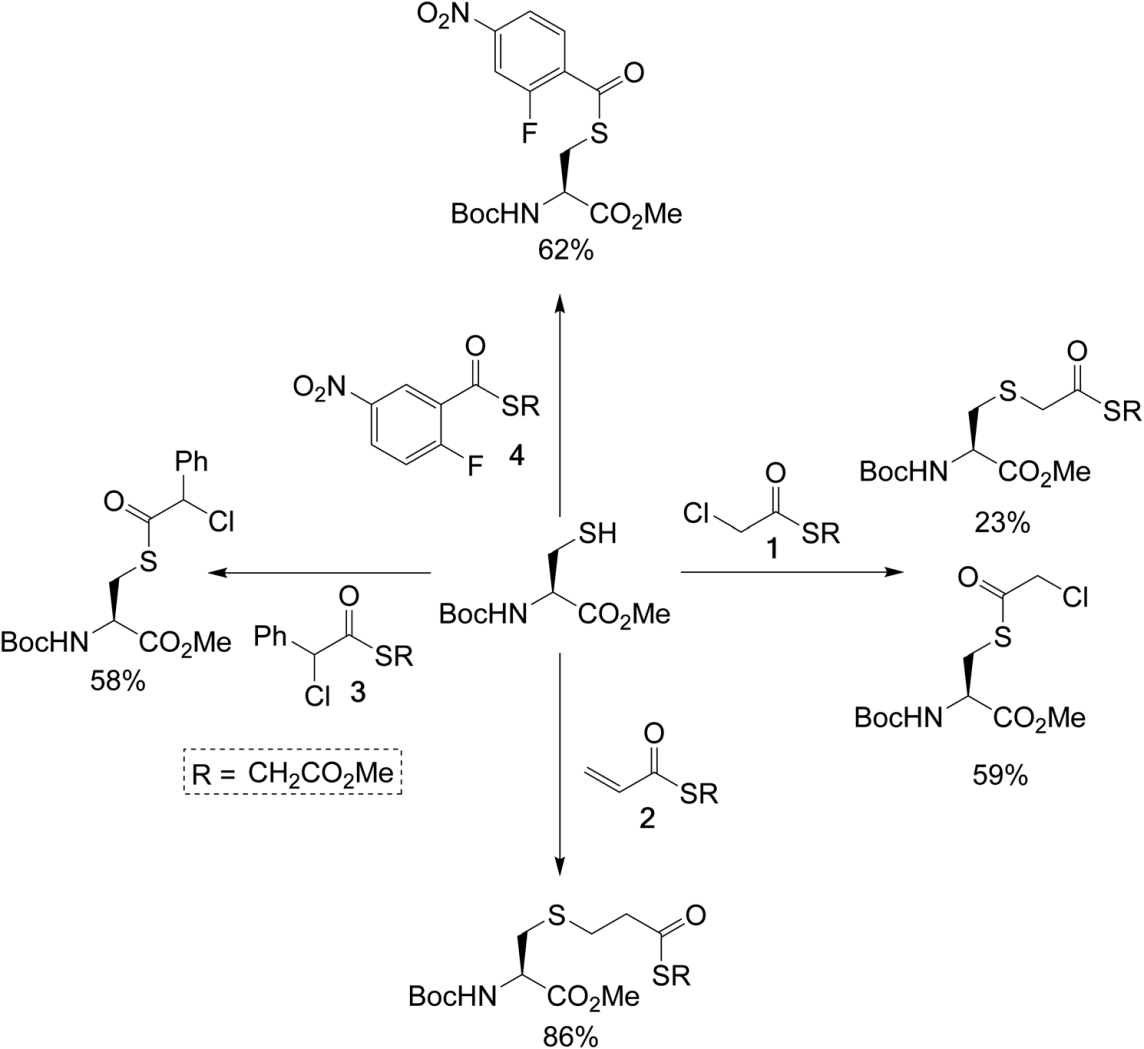
Single amino-acid model study, using *N*-Boc-Cys-OMe and treating it with the dual-reactive reagents in phosphate buffer (50 mM)/MeCN (80 : 20), pH 7.4.

To test the feasibility of this concept, we identified a panel of 4 reagents. All would contain a thioester, reactive to transthioesterification to install the labile linkage. Then α-chloro-thioesters 1 and 3 would react by S_N_2 displacement,^25^ and acrylic thioester 2 and aryl fluoride 4 by conjugate addition and S_N_Ar respectively on Fab fragment, to install the stable thioether linkages. Each of these reagents was initially reacted on *N*-Boc-Cys-OMe (50 mM phosphate buffer/MeCN (80 : 20), pH 7.4), to get information about the relative thiol reactivity of the two functional groups in each structure (Scheme 2). The product isolated in each reaction revealed that for compounds 1, 3, and 4 transthioesterification was taking place faster than the S_N_2 or S_N_Ar reactions; although notably in the case of α-chloro-thioesters 1 a minor product formed by S_N_2 displacement was also observed. For acrylic thioester 2 the conjugate addition was faster than transthioesterification, which is consistent with literature on thiol additions to related α,β-unsaturated thioesters.^26^ It should be noted that in these reactions competing thiol oxidation was also observed which prevented full conversions, and is the reason the yields are moderate.

**Fig. 1:**
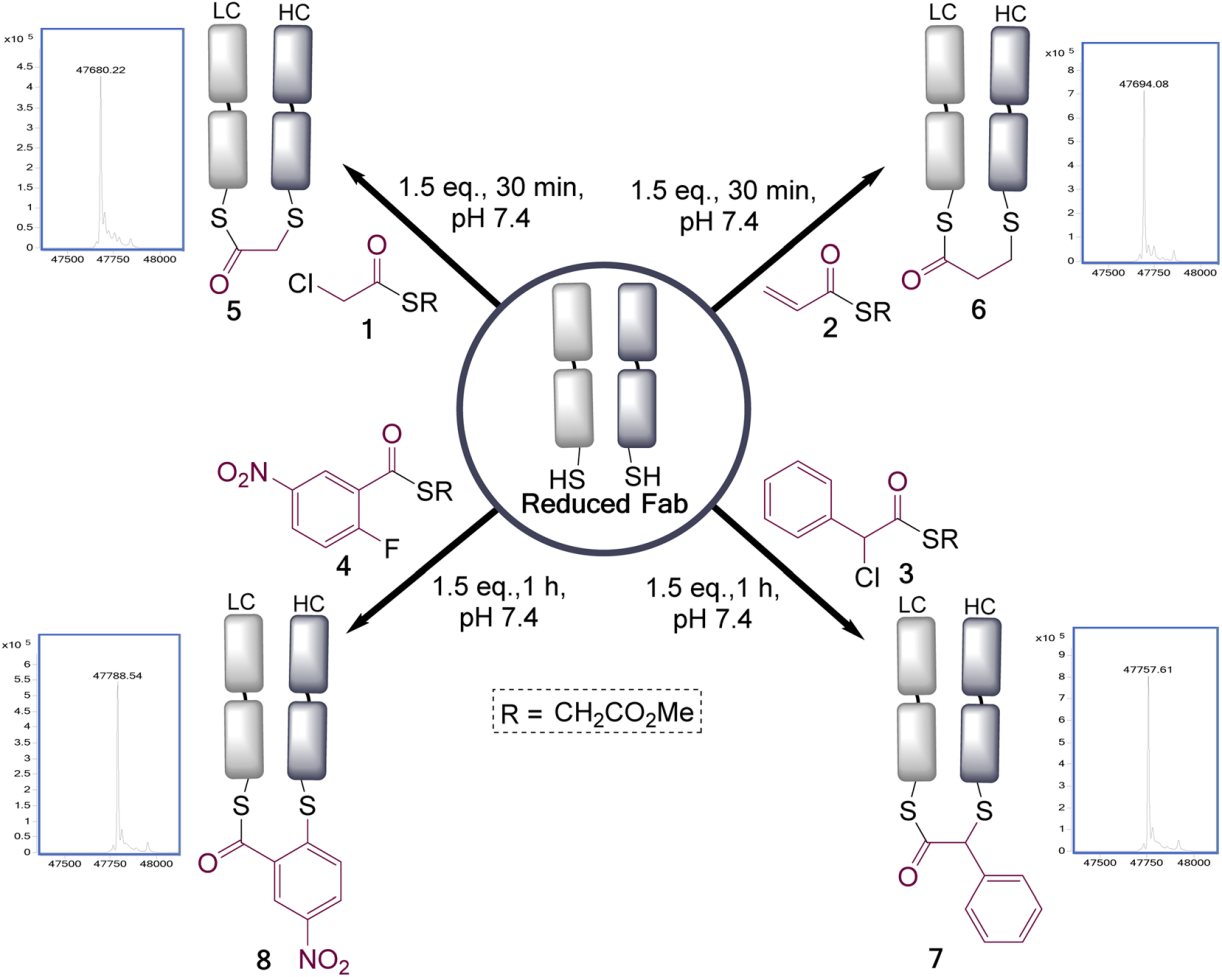
Reduced trastuzumab Fab, generated by treatment of the Fab with TCEP, is disulfide bridged with selection of dual reactivity reagents. LC = light chain, HC = heavy chain. Two regioisomers are formed in each case, with the major one is as shown (*vide infra*).

These four reagents were then applied to the Fab fragment of Her2+ targeting breast cancer drug Trastuzumab (Ontruzant). As the Fab fragment contains a single solvent accessible disulfide bond this represented an ideal system for this study (Fig. 1), and notably antibody fragment conjugates are of broad interest due to their potential for enhanced tumour penetration and tunable half-lives.^27–29^ Pleasingly, all the reagents extremely efficiently re-bridged the disulfide bond within 1 h at 22 °C, pH 7.4 (1.5 eq. of reagents added). This revealed that the rate enhancement for the bridging step, due to the proximity of the cysteine residues, was sufficient to overcome the differential in reactivity of the two functional groups present in these reagents; as no competing double addition was observed (see ESI† for full LCMS data).

To understand how stable the resultant bridged thioester conjugates were, a hydrolysis study was performed (see ESI Fig. S8, S14, S19 and S24†). To achieve complete hydrolysis of conjugates 5 and 6 in 24 h, the pH had to be increased to 8.5 (acyl transfer, presumed to be on to near-by lysines, also observed as a minor pathway competing with hydrolysis). Under these same conditions conjugate 8 had undergone only partial hydrolysis, and conjugate 7 was in-tact; demonstrating a range of reactivities can be achieved with these reagents. Interestingly, hydrolysis of these conjugates serves to cap one cysteine residue out of a pair, which is not usually practicable using classical cysteine conjugation reagents. It should also be noted that at pH 7.4 conjugate 5 had only undergone a small amount of detectable hydrolysis in 24 h (ESI Fig. S9†); however, we still favour making these bridged intermediates directly before use, to preclude any possibility of hydrolysis on storage.

**Fig. 2:**
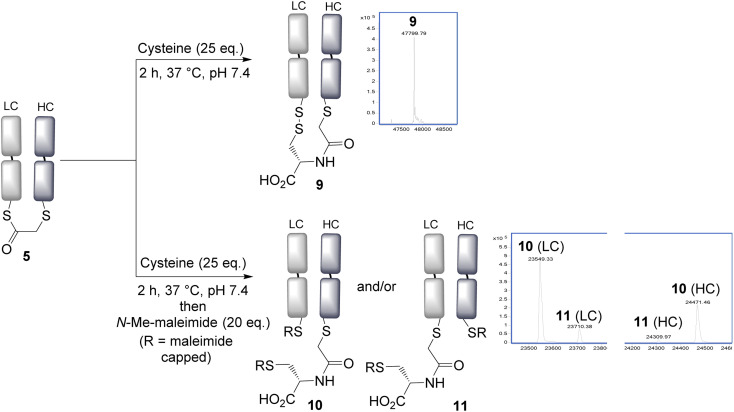
Native chemical ligation is effective using cysteine, and a new disulfide bond is formed by re-oxidation connecting the heavy and light chains. LC = light chain, HC = heavy chain.

With the panel of bridged Fab conjugates in hand, we explored the opportunity of using the incorporated thioester as a handle for NCL. This was enticing as it would allow a very convenient route to the production of antibody–peptide/protein bioconjugates, precluding the need to generate a C-terminal thioester.^30^ Reaction of the bridged thioester 5 with cysteine (25 eq., 2 h, 37 °C) was found to efficiently undergo a NCL conjugation, and LCMS analysis showed the formation of the conjugate 9. Notably the newly inserted cysteine had, as desired, oxidised to form a disulfide bond with the remaining antibody Cys, to re-establish the covalent bridge between the heavy and light chains. Alternatively, the free thiols could be capped with *N*-methylmaleimide to form conjugate 10 and 11; which allowed LCMS analysis to reveal the ratio of the two regioisomers 10 and 11 formed upon bridging (Fig. 2). An approximate 5 : 1 ratio was observed, determined by the LCMS raw data, with the major product (10) derived from the S_N_2 reaction having taken place on the heavy chain. This is consistent with this HC cysteine having a lower p*K*_a_ compared to the C-terminal light chain cysteine, and hence being more reactive and preferentially carrying out the irreversible step in the bridging reaction. NCL was observed to occur with all bridged thioesters 5–8 (see ESI Fig. S6, S12, S17 and S22†). However, the secondary chloride 7 and the aryl fluoride 8 required harsher conditions (100 eq. cysteine, 4 h, 37 °C, pH 7.4), with the latter not reaching completion under these conditions, consistent with the reduced reactivity of these thioesters. The regioselectivity in all cases resulted in the major product having the light chain cysteine forming the thioester linkage, with the highest selectivity achieved using acrylic reagent 6 (∼12 : 1, see ESI Fig. S12†). In any case, such regioisomers are unlikely to have significantly different properties given the similarity in the position of the attachments on the antibody fragment.

**Fig. 3:**
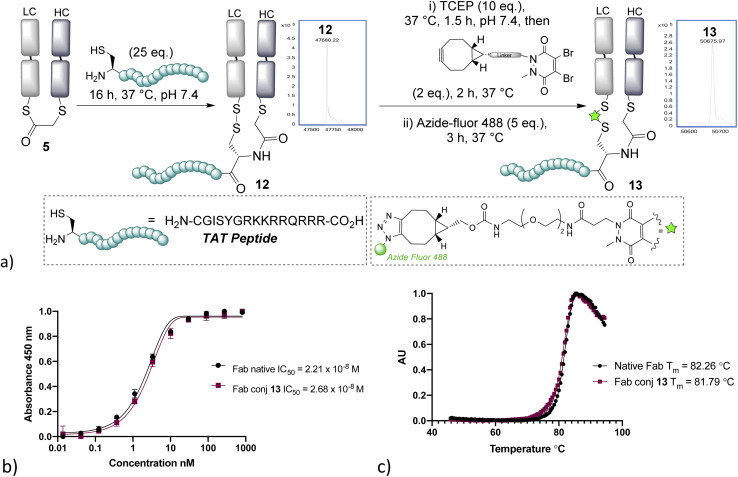
(a) Native chemical ligation on the bridged thioester with a TAT peptide affords antibody fragment–peptide conjugates; (b) ELISA study of conjugate 13, (c) thermal shift assay of conjugate 13. LC = light chain, HC = heavy chain.

With NCL on bridged thioesters validated as a viable bioconjugation protocol, we trialled the approach to the formation of an antibody-fragment peptide bioconjugate. A TAT peptide containing an N-terminal cysteine was selected, as it represented an ideal model peptide and would demonstrate the applicability of the method to the construction of antibody–cell penetrating peptide conjugates, which are of interest in controlling the internalising properties of antibodies.^31,32^ NCL on the bridged thioester 5 with the TAT peptide was found to occur effectively overnight, cleanly affording the re-oxidised conjugate 12 (Fig. 3). Furthermore, the disulfide could be re-bridged with a pyridazinedione (PD)-BCN^33^ and then post-clicked with Azide-fluor 488 to afford a fluorescently labelled antibody–peptide dual conjugate 13. UV/Vis absorbance gave a fluorophore-to-antibody ratio (FAR) of 0.9, providing further evidence of conjugation efficiency to support the LCMS data. Notably, dual clickable PDs have also been reported by us previously in disulfide bridging, and their use could be envisaged in this sequence, if desired, for the construction of triconjugates.^5,12^ HER2 ELISA and thermal shift assay on conjugate 13 revealed no decrease of binding and no significant loss of thermal stability, which is in line with published reports; and consistent with modification being distal from the complementary determining regions (CDRs) and the reformation of the covalent linkage between chains retaining stability.^34^

**Fig. 4:**
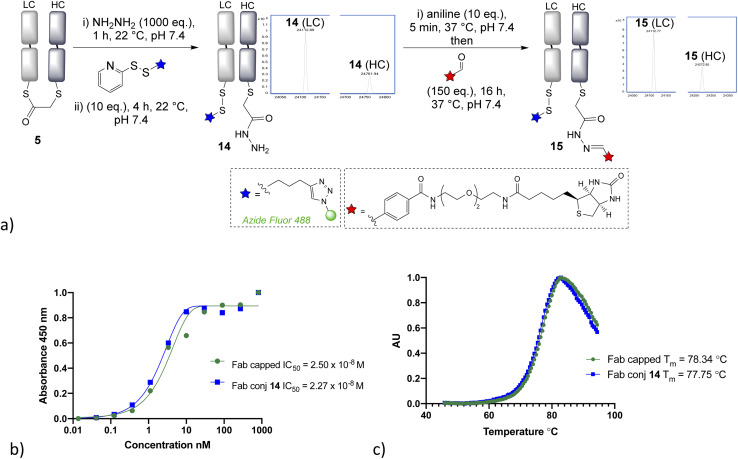
(a) Hydrazine addition to the bridged thioester enables dual conjugation approach, demonstrated by the formation of cleavable disulfide and hydrazone linkages, (b) ELISA assay of conjugate 14, (c) thermal shift assay of conjugate 14. LC = light chain, HC = heavy chain.

Having demonstrated that α-chlorothioester 1 re-bridging of the Fab disulfide enables bioconjugation using N-terminal cysteine containing peptides *via* NCL, we investigated the possibility of adding different amine nucleophiles to afford alternative dual conjugates. Whilst the retention of the covalent link between heavy and light chains is likely to afford extra stability, there is no strong evidence that this is essential for the majority of viable applications of such antibody conjugates; as the chains are strongly bound by intermolecular forces.^35^ A screen of several amines revealed that the bridged thioester was insufficiently reactive to achieve efficient conjugation with most primary or secondary amines under the buffered conditions (1 h, pH 7.4, 37 °C, 1000 eq. of aniline, pyrrolidine, benzylamine, piperidine, *p*-anisidine, propargylamine, hydroxylamine). However it was found that hydrazine, which is a useful nucleophile for hydrazone bioconjugations,^36^ was effective (Fig. 4). Addition of 1000 eq., pH 7.4 for 1 h, afforded a hydrazide liberating a cysteine which could then be employed in a subsequent bioconjugation. We elected to form a disulfide, using pyridyldisulfide-azide fluor reagent, as it would represent a cleavable linkage for future applications. Notably the formation of disulfide bonds from native cysteine residues in antibodies is not normally possible, as the adjacent cysteines are prone to re-oxidise with each other preferentially. The dual modified conjugate 14, successfully formed, was analysed by LCMS in the form of the heavy and light chain conjugates (notably the minor regioisomer in these final conjugates is variable in how well it is detected in the LCMS traces). Finally, hydrazone ligation with biotin-aldehyde was carried out to demonstrate controlled formation of variably cleavable dual conjugates 15. Furthermore, the biotin tag could be directly used in such conjugates, for example avidin pull down assays for purification purposes. The disulfide containing linker was shown to be cleavable in presence of 5 mM GSH at 37 °C in 4 h (see ESI Fig. S35†), which is in line with published reports.^37,38^ The stability of such disulfide bonds can be extended if desired by addition of steric hindrance, such as an adjacent methyl group.^39^ The hydrazone bond remained stable at pH 4–6, as expected due to the electron poor aromatic linker used,^36,40^ and more electron rich aromatic or aliphatic linkers could be employed if pH controlled cleavability was desired.^41–43^ The ELISA study again demonstrated full retention of binding activity for the dual conjugate, whereas the thermal stability showed a decrease of 4 °C for the capped-reduced Fab and dual conjugate in comparison to native Fab fragment (see Fig. 4). This confirms that the loss of the disulfide bridge does indeed lead to a detectable reduction in stability, although as stated it is unclear that this would preclude any applications given the relatively high *T*_m_.

In conclusion, we have demonstrated that dual reactivity ‘stable-labile’ disulfide bridging reagents offer a new approach to the construction of bioconjugates, and numerous designs can be conceived and explored going forwards. Despite having two functional groups with varying cysteine reactivity, we showed that a diverse selection of such dual-reactive reagents, with mechanisms of conjugation reactions including S_N_2, conjugate addition and S_N_Ar, efficiently bridged the reduced disulfide bond of a Fab fragment. By inserting one stable linkage (*i.e.* a thioether), and one labile linkage (*i.e.* a thioester), these reagents afford a robust attachment to the protein with one end of the bridge whilst offering the other end as a site for subsequent ligation. The direct attachment of a N-terminal cysteine containing peptides by native chemical ligation could then be carried out, representing an extremely convenient approach to form such antibody fragment–peptide conjugates; and precluding the need to produce a C-terminal thioester. Alternatively, the use of hydrazine as the ligating nucleophile enabled a separate cargo to be attached to each cysteine residue, which could be exploited to insert variably cleavable linkers such as a disulfide and a hydrazone. These methodologies expand the scope of disulfide bridging reagents and offer an extremely convenient route to the construction of multifunctional antibody fragment conjugates.

## Author contributions

J. R. B., V. C. and A. C. conceived and designed the project; J. R. B., V. C. and A. C. conceived and designed the experiments; A. C. carried out the experimental work; I. A. T. carried out some of the chemistry experiments; J. A. I. contributed to the thermal-shift assay; J. R. B. and A. C. wrote the manuscript with contributions from the other authors.

## Conflicts of interest

The authors declare no competing financial interests.

## Supplementary Material

SC-013-D2SC04531A-s001
