# Supplementary material for: Dual reactivity disulfide bridging reagents; enabling new approaches to antibody fragment bioconjugation
Source: Chem Sci. 2022 Sep 27;13(39):11533–9. doi: 10.1039/d2sc04531a (PMC9555722; doi:10.1039/d2sc04531a)
Supplement: SC-013-D2SC04531A-s001 [file SC-013-D2SC04531A-s001.pdf]

Electronic Supplementary Material (ESI) for Chemical Science.
This journal is © The Royal Society of Chemistry 2022

Electronic Supplementary Information

Dual reactivity of disulfide bridging reagents for antibody bioconjugation

Alina Chrzastek[1], Ioanna A. Thanasi[1],
James Irving[2], Vijay Chudasama[*1], James R. Baker[*1]

[1]Department of Chemistry, University College London, 20 Gordon Street, WC1H OAJ, London, United Kingdom

[2]UCL Respiratory, Rayne Institute, University College London, WC1E 6JF, London,

* E-mails: j.r.baker@ucl.ac.uk, v.chudasama@ucl.ac.uk

Table of Contents

Chemical synthesis general remarks 4

Synthesis and characterisation of compounds 4

Methyl 2-((2-chloroacetyl)thio)acetate (1) 4

Methyl 6-(methoxycarbonyl)-2,2-dimethyl-4,10-dioxo-3-oxa-8,11-dithia-5-azatridecan-13-oate (S1) and methyl *N*-(*tert*-butoxycarbonyl)-*S*-(2-chloroacetyl)cysteinate (S2) 6

methyl 2-(acryloylthio)acetate (2) 10

methyl (*S*)-6-(methoxycarbonyl)-2,2-dimethyl-4,11-dioxo-3-oxa-8,12-dithia-5-azatetradecan-14-oate (S3) 11

methyl 2-((2-chloro-2-phenylacetyl)thio)acetate (3) 13

methyl *N*-(*tert*-butoxycarbonyl)-*S*-(2-chloro-2-phenylacetyl)-*L*-cysteinate (S4) 14

2-fluoro-5-nitrobenzoic acid (S5) 16

methyl 2-((2-fluoro-5-nitrobenzoyl)thio)acetate (4) 17

methyl *N*-(*tert*-butoxycarbonyl)-*S*-(2-fluoro-5-nitrobenzoyl)-*D*-cysteinate (S6) 19

pent-4-yne-1-thiol[1] (S7) 21

2-(pent-4-yn-1-yldisulfaneyl)pyridine (S8) 23

***tert*-butyl (2-(2-(2-aminoethoxy)ethoxy)ethyl)carbamate[2] (S9)** 24

***tert*-butyl (2-(2-(2-(5-((3a*S*,4*S*,6a*R*)-2-oxohexahydro-1*H*-thieno[3,4-*d*]imidazol-4-yl)pentanamido)ethoxy)ethoxy)ethyl)carbamate[2] (S10)** 26

4-formyl-*N*-(2-(2-(2-(5-((3a*R*,4*R*,6a*S*)-2-oxohexahydro-1*H*-thieno[3,4-*d*]imidazol-4-yl)pentanamido)ethoxy)ethoxy)ethyl)benzamide[2] (S11) 28

((1*R*,8*S*,9*s*)-bicyclo[6.1.0]non-4-yn-9-yl)methyl (2-(2-(2-(3-(4,5-dibromo-2-methyl-3,6-dioxo-3,6-dihydropyridazin-1(2*H*)-yl)propanamido)ethoxy)ethoxy)ethyl)carbamate[3] (S12) 30

Bioconjugation general marks 30

LCMS general remarks 31

Native Fab 32

Reduced Fab 33

Reaction of Fab with α-chlorothioester (1) 34

Reaction of Fab conjugate 5 and cysteine 35

Reaction of Fab conjugate 5 with cysteine and *N*-Me-maleimide 36

Stability study of Fab conjugate 5 38

Reaction of Fab with methyl 2-(acryloylthio)acetate (2) **39**

Reaction of Fab conjugate 6 with cysteine and *N*-Me-maleimide **41**

Stability study of Fab conjugate 6 .... **43**

Reaction of Fab with methyl 2-((2-chloro-2-phenylacetyl)thio)acetate (3) **44**

Reaction of Fab conjugate 7 with cysteine and *N*-Me-maleimide **45**

Stability study of Fab conjugate 7 .... **47**

Reaction of Fab with methyl 2-((2-fluoro-5-nitrobenzoyl)thio)acetate (4) **48**

Reaction of Fab conjugate 8 with cysteine and *N*-Me-maleimide **50**

Stability study of Fab conjugate 8 .... **52**

Reaction of Fab with α-chlorothioester (1) and TAT peptide **53**

Reaction of Fab with α-chlorothioester (1) with TAT peptide and BCN-PD **55**

Reaction of Fab with α-chlorothioester (1) with TAT peptide, BCN-PD and Azide-fluor 488 .... **57**

Reaction of Fab with α-chlorothioester (1), hydrazine hydrate, pyridyldisulfide and Azide-Fluor 488 .... **59**

Reaction of Fab with α-chlorothioester (1), hydrazine hydrate, pyridyldisulfide, Azide-fluor 488, and biotin-aldehyde **61**

Dual conjugation – disulfide cleavage **63**

Enzyme-linked immunosorbent assay (ELISA) – Trastuzumab against HER2 .... **65**

Thermal Shift Assay **66**

References **66**

Chemical synthesis general remarks

All chemical reagents and solvents were purchased from commercial sources; Sigma UK, Fisher UK or VWR UK and used as per manufacturer instructions. Buffers were prepared with double-deionised water and filter sterilised (0.20 μm). All chemical reactions were carried out at atmospheric pressure, under argon. Room temperature (RT) is defined as between 15-25 °C. The term *in vacuo* refers to organic solvent removal using Buchi rotary evaporator between 15-60 °C. Chemical reactions were monitored using thin layer chromatography (TLC) on pre-coated silica gel plates (254 μm) purchased from VWR, UK. Detection of synthesised compounds was done by UV (254 nm and 365 nm) or chemical stain ($KMnO_4$, ninhydrin). Flash column chromatography was carried out using pre-loaded FlashPure Eco Flex column on Biotage Isolera Spektra One flash chromatography system. ^{1}H NMR and ^{13}C NMR were obtained at ambient temperature on a Bruker Advance AMX600 instrument operating at 600 MHz or 700 MHz ^{1}H and 150 MHz for ^{13}C in the stated solvent. Chemical shifts (δ) are reported in parts per million (ppm) and coupling constants (*J*) in Hertz (Hz). The multiplicity of each signal is indicated as s-singlet, d-doublet, t-triplet, q-quartet, quin-quintet, m-multiplet (i.e., complex peak obtained due to overlap) or a combination of these. All assignments were made with the aid of DEPT, COSY, HSQC, HMBC, or NOESY corelation experiments. Infra-red spectra were recorded on a Bruker ALPHA FT-IR spectrometer operating in ATR mode, with frequencies given in reciprocal centimetres (cm^{-1}). Melting points were taken on a Gellenkamp apparatus and are uncorrected. High and low resolution mass spectra were recorded on a VG70 SE mass spectrometer, operating in modes ESI, EI, or CI (+ or -) depending on the sample, at the Department of Chemistry, University College London. Buffers used in organic reactions were prepared with double-deionised water and filter sterilised (0.20 μm); 50 mM Phosphate Buffer, pH 6.75; 5 mM glutathione in 50 mM Phosphate Buffer, pH 6.5.

Synthesis and characterisation of compounds

Methyl 2-((2-chloroacetyl)thio)acetate (1)

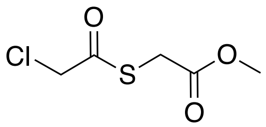

Methyl thioglycolate (0.28 mL, 3.14 mmol, 1.0 eq.) and triethylamine (0.44 mL, 3.14 mmol, 1.0 eq.) were combined in DCM (2.0 mL). The mixture was then added dropwise over 2 h into a stirring solution of chloroacetyl chloride (0.75 ml, 9.43 mmol, 3.0 eq.) in DCM (10.0 mL) and this was left O/N. The reaction was performed at room temperature, under argon atmosphere and constant agitation. The solvent was then removed *in vacuo* and purification by column chromatography (gradient elution from 100%

cyclohexane to 40% EtOAc in cyclohexane) afforded the target compound as light-yellow oil (484 mg, 2.65 mmol, 84% yield).

^{1}H NMR (700 MHz, $CDCl_3$) δ_H 4.22 (s, 2H), 3.72 (s, 2H), 3.71 (s, 3H); ^{13}C (150 MHz, $CDCl_3$) δ_C 193.2 (C), 168.6 (C), 53.05 (CH_3), 47.9 (CH_2), 31.7 (CH_2); IR (oil) V_{max}/cm^{-1} 3003, 2955, 1794, 1680; LRMS (ESI) *m/z* (%) 183.0 ([^{35}M+H]$^+$, 100), 185.0 ([^{37}M+H]$^+$, 30); HRMS (ESI) *m/z* calculated for [$C_5H_7{}^{35}ClO_3S$] 182.9883, observed 182.9888.

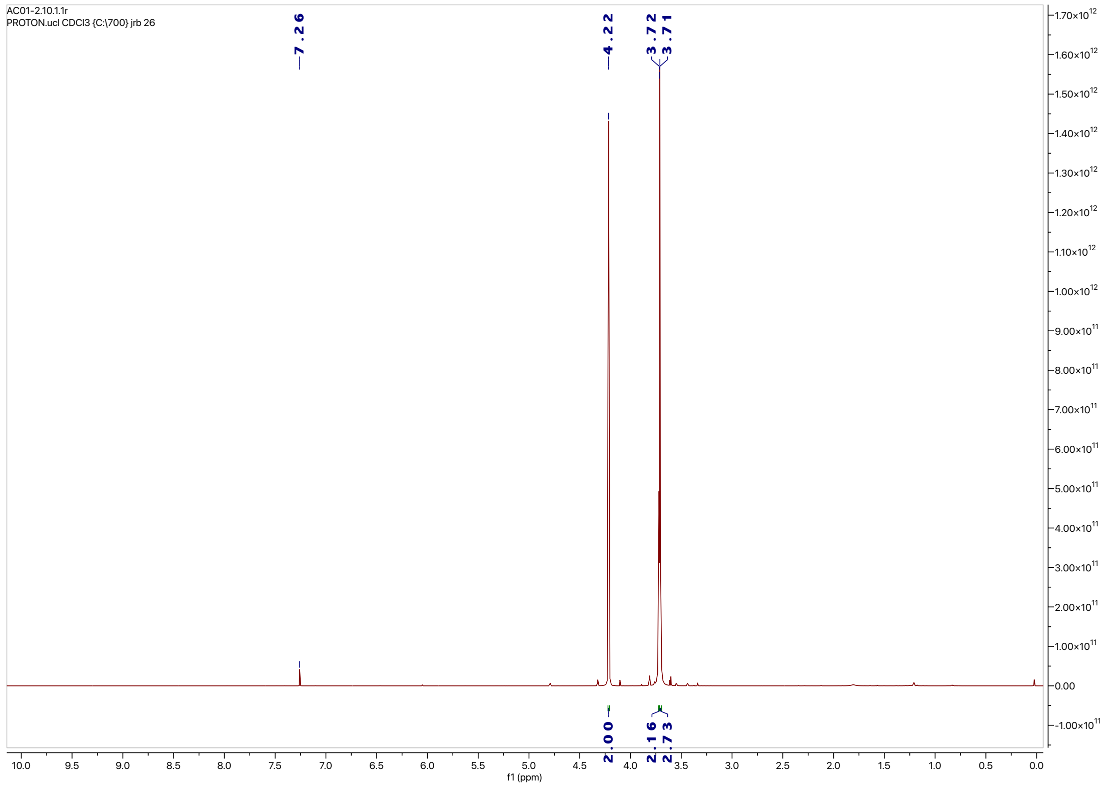

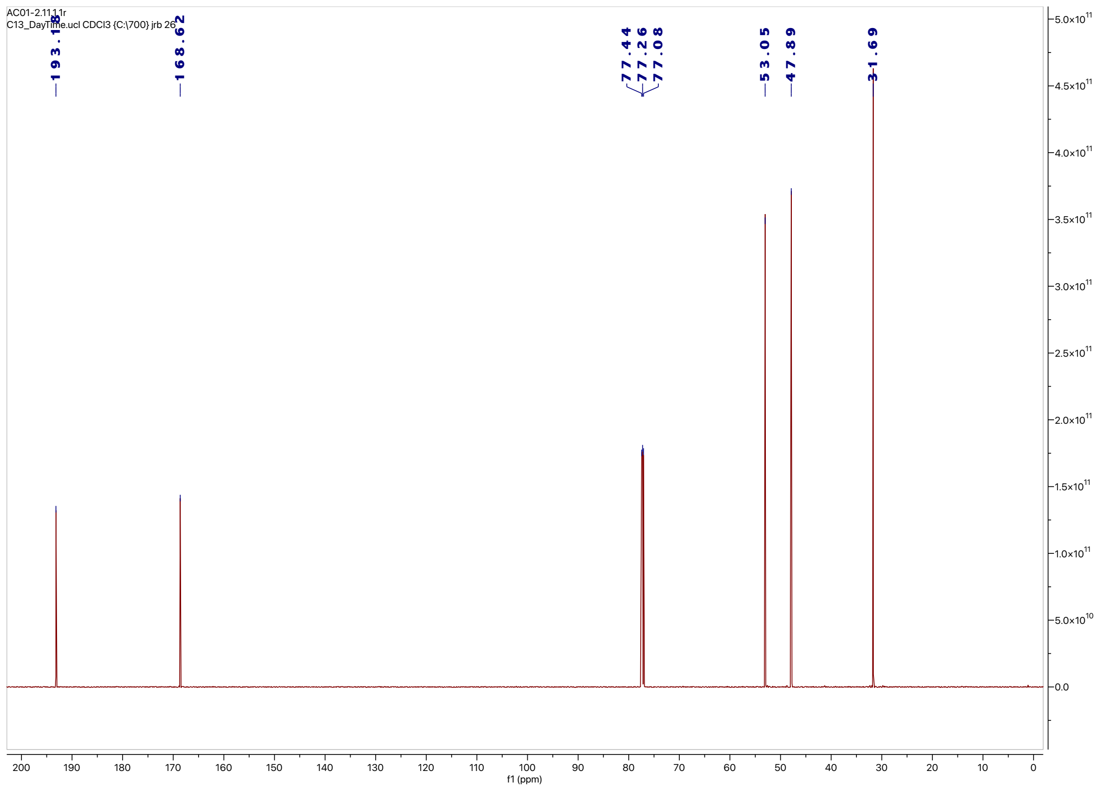

Methyl 6-(methoxycarbonyl)-2,2-dimethyl-4,10-dioxo-3-oxa-8,11-dithia-5-azatridecan-13-oate (S1) and methyl *N*-(*tert*-butoxycarbonyl)-*S*-(2-chloroacetyl)cysteinate (S2)

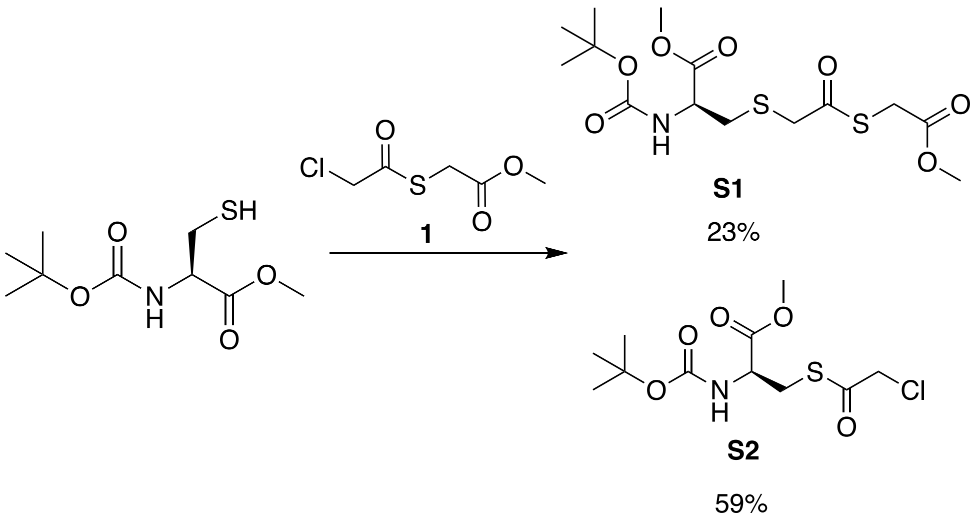

Methyl 2-((2-chloroacetyl)thio)acetate **1** (0.04 g, 0.24 mmol, 1.1 eq.) was dissolved in 0.5 mL of MeCN followed by addition of 0.7 mL of 50 mM Phosphate Buffer pH 6.75 (addition of MeCN will increase pH to 7.4). *N*-Boc-L-cysteine methyl ester (0.05 g, 0.22 mmol, 1.0 eq.) was then added and the mixture was stirred at room temperature for 3 h. The solvent was then removed *in vacuo* and purification by column chromatography (gradient elution from 100% cyclohexane to 30% EtOAc in cyclohexane) afforded compounds **S1** and **S2**.

S1 (clear oil, 19.8 mg, 0.05 mmol, 23% yield): ^{1}H NMR (600 MHz, $CDCl_3$) δ_H 5.41 (s, 1H), 4.61-4.60 (m, 1H), 4.24 (s, 2H), 3.78 (s, 3H), 3.75 (s, 3H), 3.75 (s, 2H), 3.01-2.93 (m, 2H), 1.44 (s, 9H); ^{13}C (150 MHz, $CDCl_3$) δ_C 193.3 (C), 171.0 (C), 168.7 (C), 155.3 (C), 80.4 (C), 54.9 (C), 53.1 (CH_3), 52.9 (CH_3), 47.9 (CH_2), 31.7 (CH_2), 28.4 (CH_3), 27.5 (CH_2); IR (oil) V_{max}/cm^{-1} 2978, 2954, 1741, 1705, 1596; LRMS (ESI) *m/z* (%) 282.0 ([M–Boc]$^+$, 55), 382.1 ([M+H]$^+$, 85), 404.1 ([M + Na]$^+$, 100); HRMS (ESI) *m/z* calculated for [$C_{14}H_{23}NO_7S_2$] 381.0915, observed 381.0988.

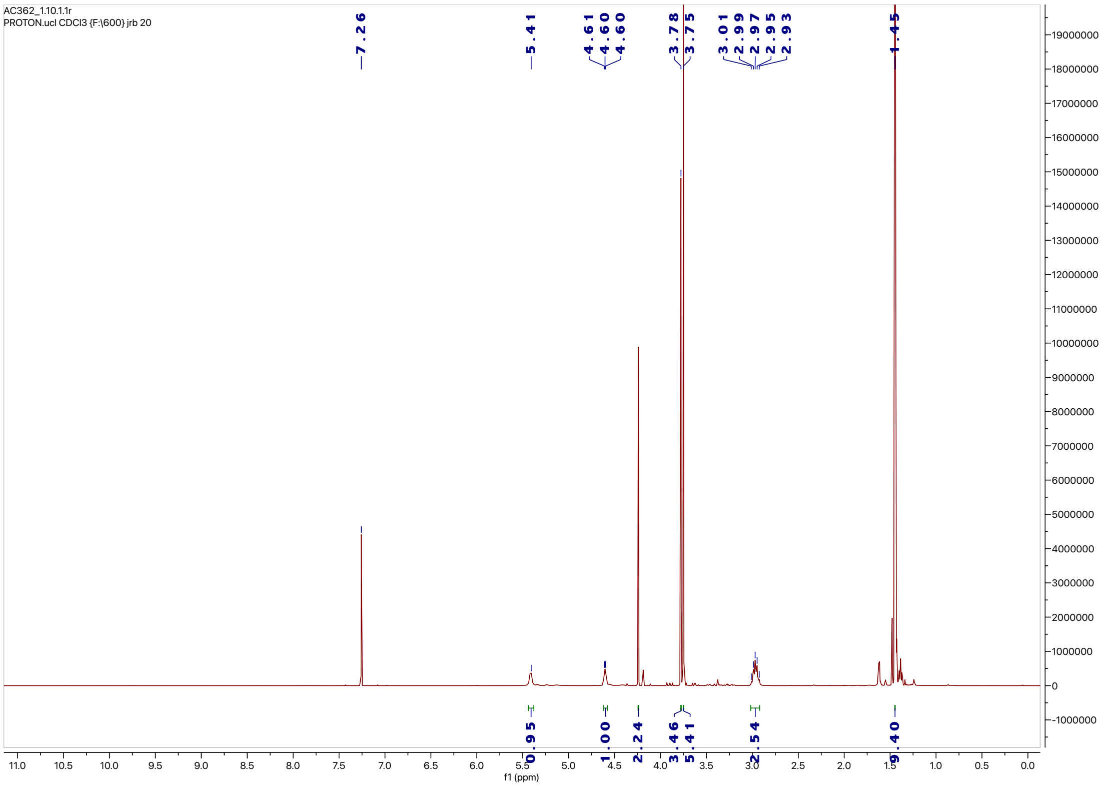

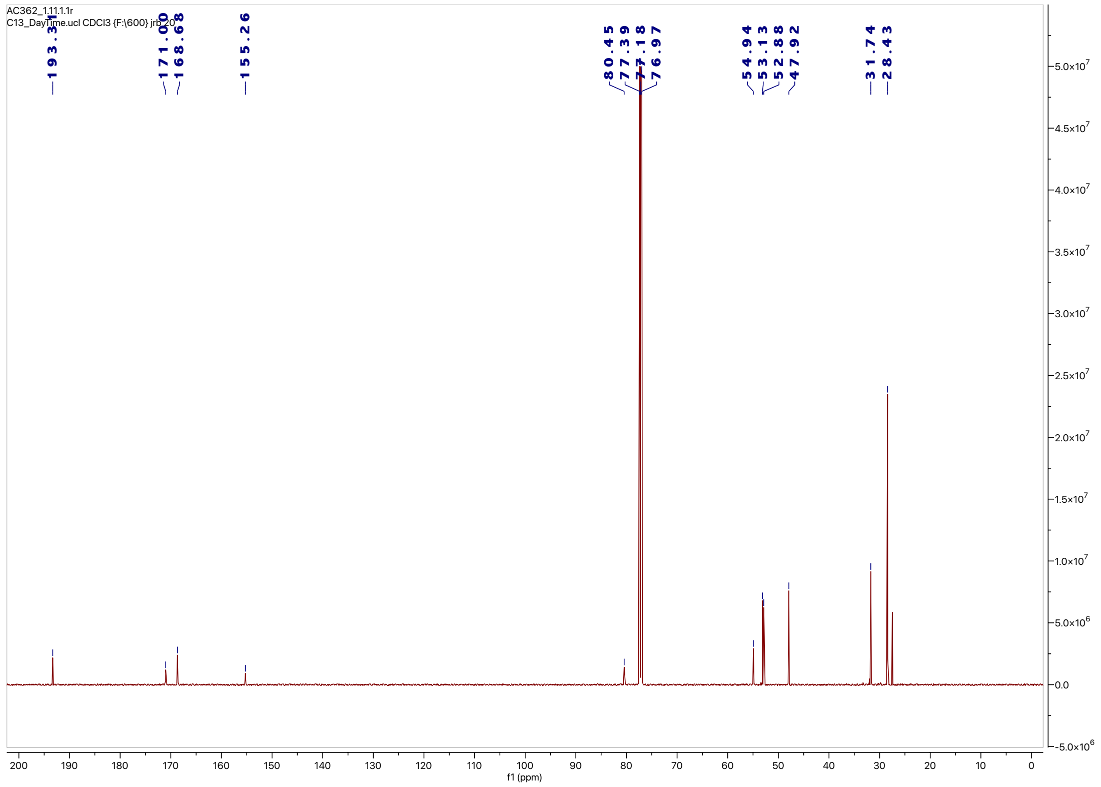

S2 (clear oil, 40.1 mg, 0.13 mmol, 59% yield): ^{1}H NMR (600 MHz, $CDCl_3$) δ_H 5.24 (s, 1H), 4.57-4.54 (m, 1H), 4.18 (s, 2H), 3.75 (s, 3H), 3.48-3.45 and 3.36-3.33 (m, 2H), 1.43 (s, 9H); ^{13}C (150 MHz, $CDCl_3$) δ_C 193.6 (C), 170.9 (C), 155.2 (C), 80.5 (C), 53.0 (CH_3), 52.8 (CH), 48.0 (CH_2), 31.9 (CH_2), 28.4 (CH_3); IR (oil) V_{max}/cm^{-1} 2979, 2954, 1744, 1706; LRMS (ESI) *m/z* (%) 312.0 ([^{35}M+H]$^+$, 30), 314.0 ([^{37}M+H]$^+$, 10), 212.0 ([^{35}M – Boc]$^+$, 80); HRMS (ESI) *m/z* calculated for [$C_{11}H_{18}{}^{35}ClNO_5S$] 311.0594, observed 311.0484.

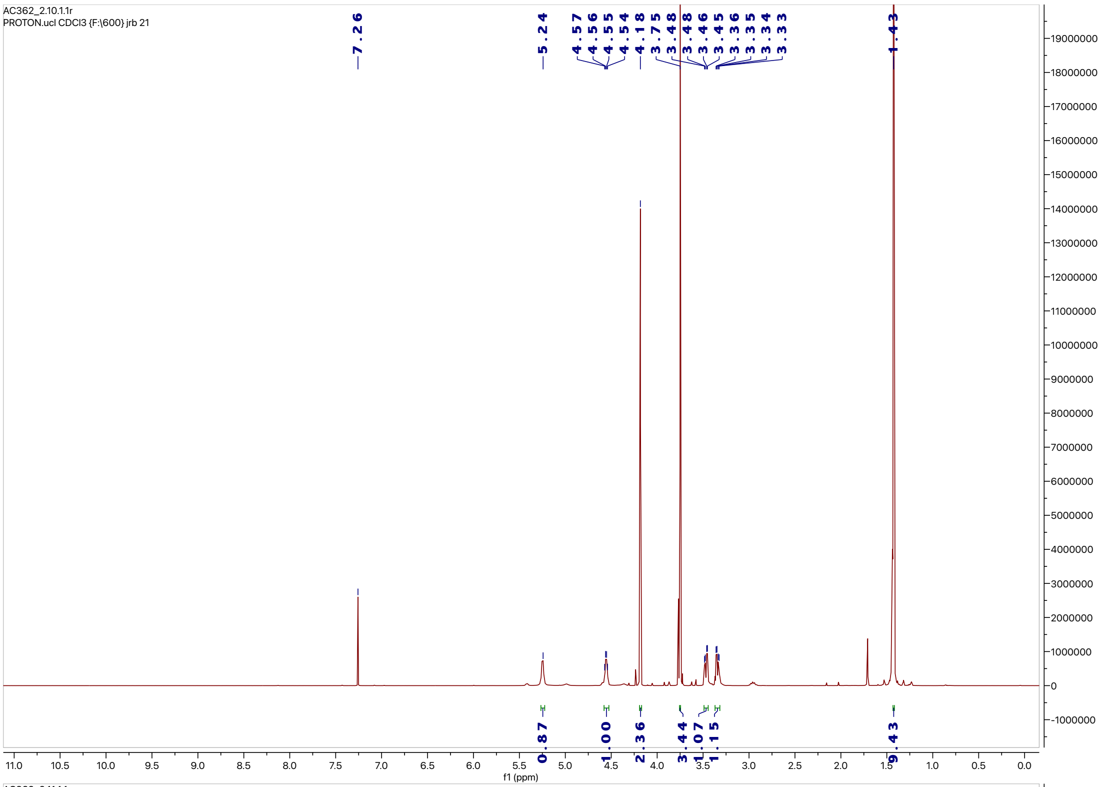
AC362_2.10.1.1r
PROTON.ucl CDCl3 {F:\600} jrb 21
7.26
5.24
4.57
4.56
4.55
4.54
4.18
3.75
3.48
3.46
3.45
3.36
3.35
3.34
3.33
1.43
0.87
1.00
2.36
3.44
1.07
1.15
9.43
f1 (ppm)

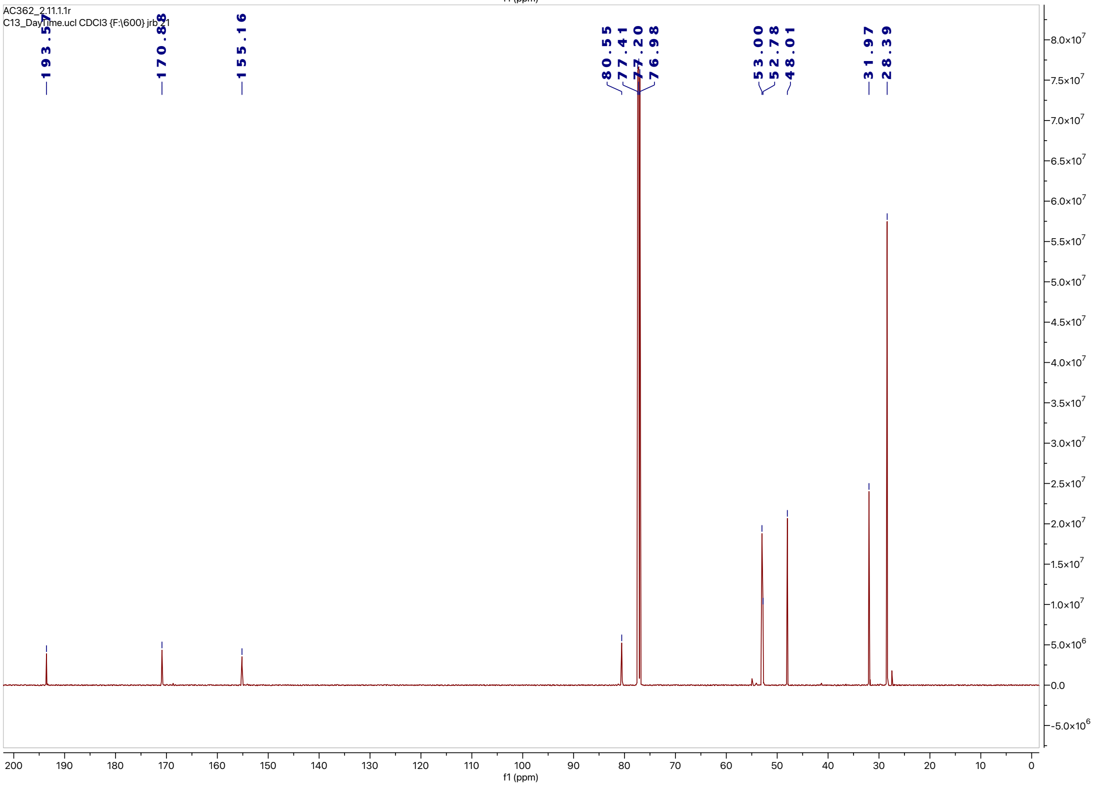
AC362_2.11.1.1r
C13_DayTime.ucl CDCl3 {F:\600} jrb 21
193.57
170.88
155.16
80.55
77.41
77.20
76.98
53.00
52.78
48.01
31.97
28.39
f1 (ppm)

methyl 2-(acryloylthio)acetate (2)

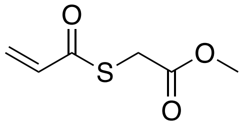

Methyl thioglycolate (0.20 mL, 2.24 mmol, 1.0 eq.) and triethylamine (0.17 mL, 2.24 mmol, 1.0 eq.) were combined in DCM (2.0 mL). The mixture was then added dropwise over 2 h into a stirring solution of acryloyl chloride (0.68 mL, 6.72 mmol, 3.0 eq.) in DCM (10.0 mL), this was left O/N. The reaction was performed at room temperature, under argon atmosphere and constant agitation. The solvent was then removed *in vacuo* and purification by column chromatography (gradient elution from 100% cyclohexane to 30% EtOAc in cyclohexane) afforded the target compound as colourless waxy oil (183 mg, 1.14 mmol, 51% yield).

^{1}H NMR (600 MHz, $CDCl_3$) δ_H 6.42-6.33 (m, 2H), 5.77 (dd, 1H, *J* = 9.7, 1.2 Hz), 3.77 (s, 2H), 3.73 (s, 3H); ^{13}C (150 MHz, $CDCl_3$) δ_C 188.6 (C), 169.3 (C), 134.3 (CH_2), 128.0 (CH), 53.0 (CH_3), 30.9 (CH_2); IR (oil) V_{max}/cm^{-1} 2953, 1737, 1673; LRMS (ESI) *m/z* (%) 161.0 ([M+H]$^+$, 100); HRMS (ESI) *m/z* calculated for [$C_6H_8O_3S$] 160.0194, observed 160.0268.

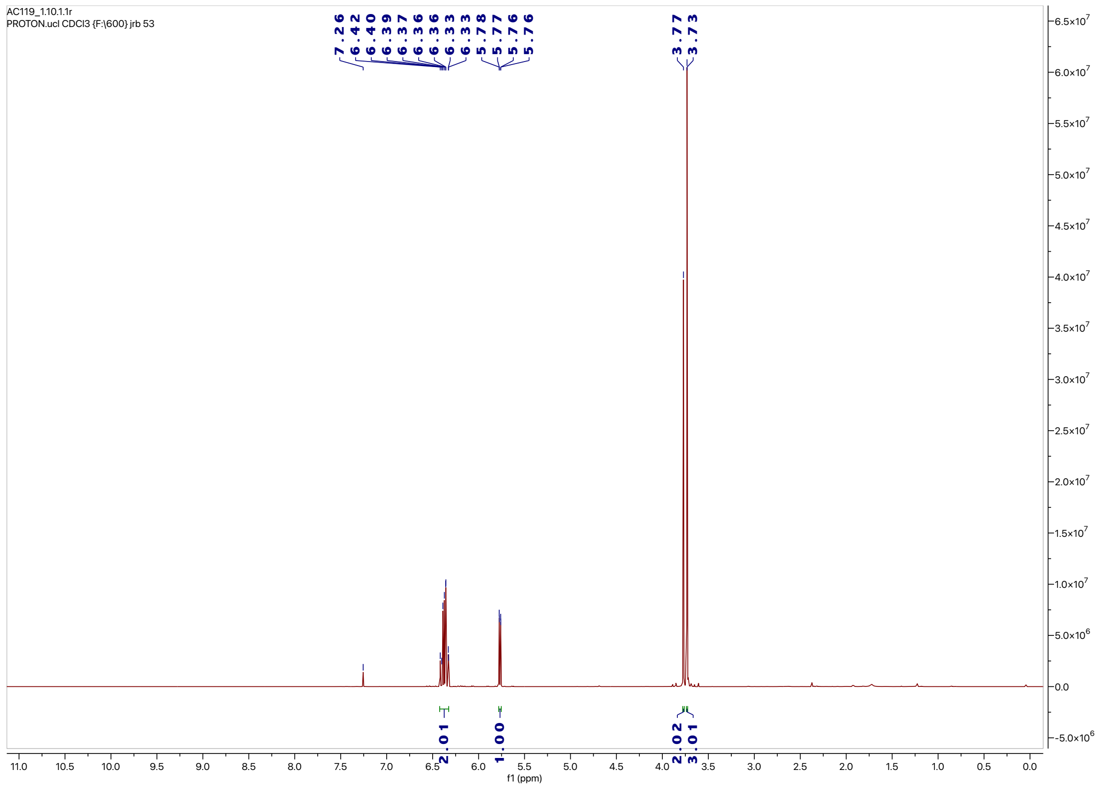

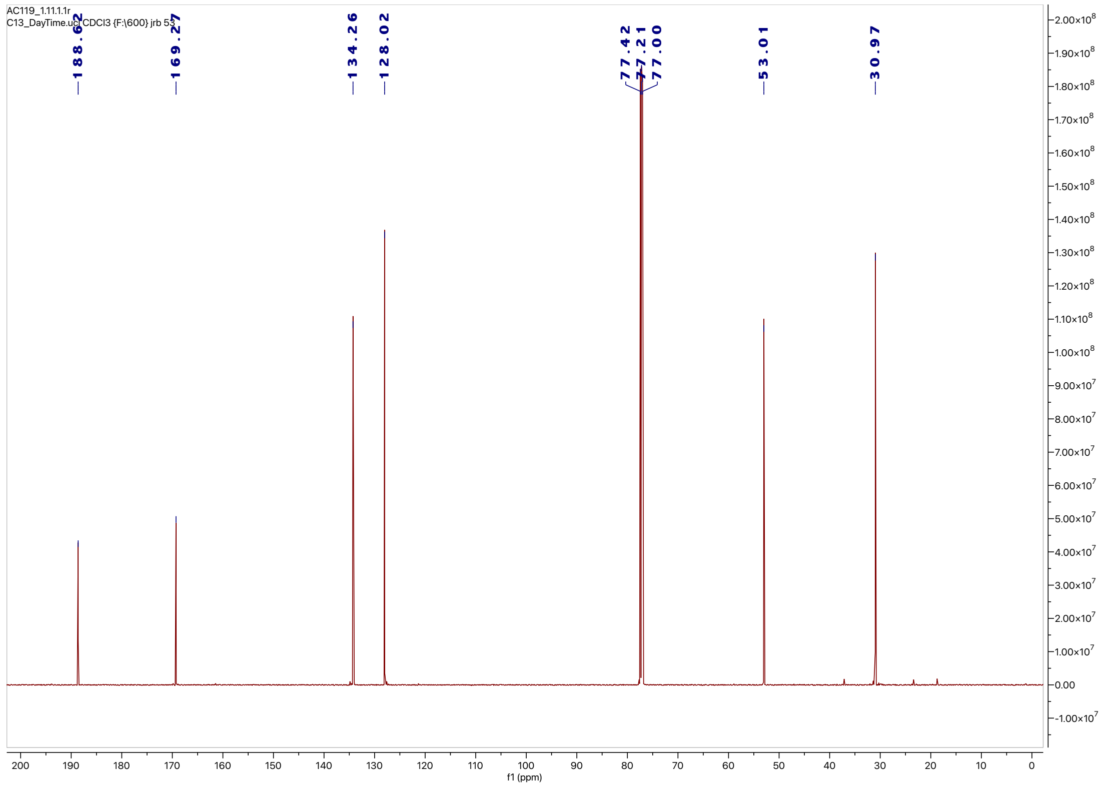

methyl (*S*)-6-(methoxycarbonyl)-2,2-dimethyl-4,11-dioxo-3-oxa-8,12-dithia-5-azatetradecan-14-oate (S3)

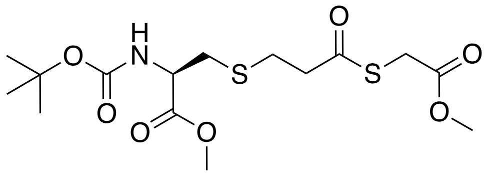

Methyl 2-(acryloylthio)acetate **2** (0.05 ml, 0.30 mmol, 3.0 eq.) was dissolved in 0.5 mL of MeCN followed by addition of 0.7 mL of 50 mM Phosphate Buffer pH 6.75 (addition of MeCN will increase pH to 7.4). *N*-Boc cysteine methyl ester (0.02 g, 0.10 mmol, 1.0 eq.) was then added and the mixture was stirred for 3 h at room temperature. The solvent was then removed *in vacuo* and purification by column chromatography (gradient elution from 100% cyclohexane to 40% ethyl acetate in cyclohexane) afforded the target compound as clear oil (33.8 mg, 0.09 mmol, 86% yield*). *Minor impurities observed in the NMR of ethyl acetate.

^{1}H NMR (600 MHz, $CDCl_3$) δ_H 5.37 (d, 1H, *J* = 8.1 Hz), 4.53-4.50 (m, 1H), 3.75 (s, 3H), 3.72 (s, 3H), 3.70 (s, 2H), 3.00-2.93 (m, 2H), 2.88-2.85 (m, 2H), 2.82-2.80 (m, 2H), 1.42 (s, 9H); ^{13}C (150 MHz, $CDCl_3$) δ_C 195.6 (C), 171.5 (C), 169.1 (C), 155.3 (C), 80.4 (C), 53.4 (CH), 52.9 (CH_3), 52.8 (CH_3), 43.7 (CH_2), 34.9 (CH_2), 31.2

(CH_2), 28.4 (CH_3), 27.7 (CH_2); IR (oil) V_{max}/cm^{-1} 2977, 2954, 1741, 1694; LRMS (ESI) *m/z* (%), 396.1 ([M+H]$^+$, 100); HRMS (ESI) *m/z* calculated for [$C_{15}H_{25}NO_7S_2$] 395.1072, observed 395.1137.

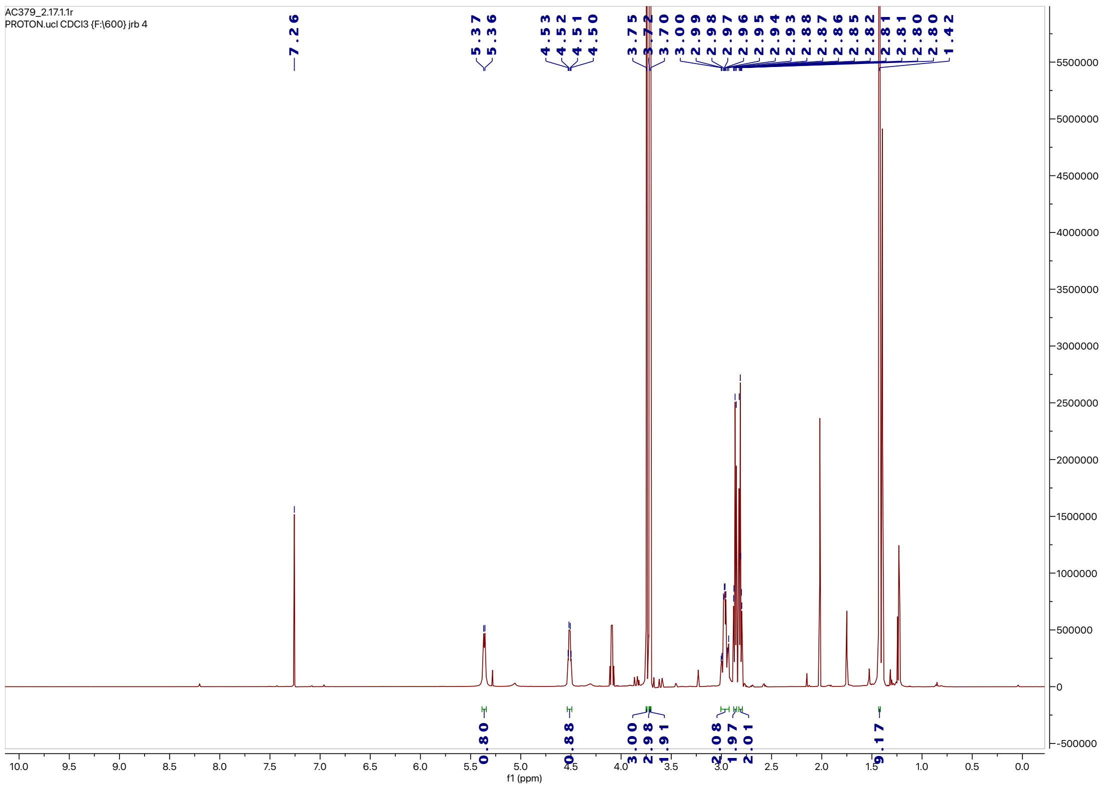

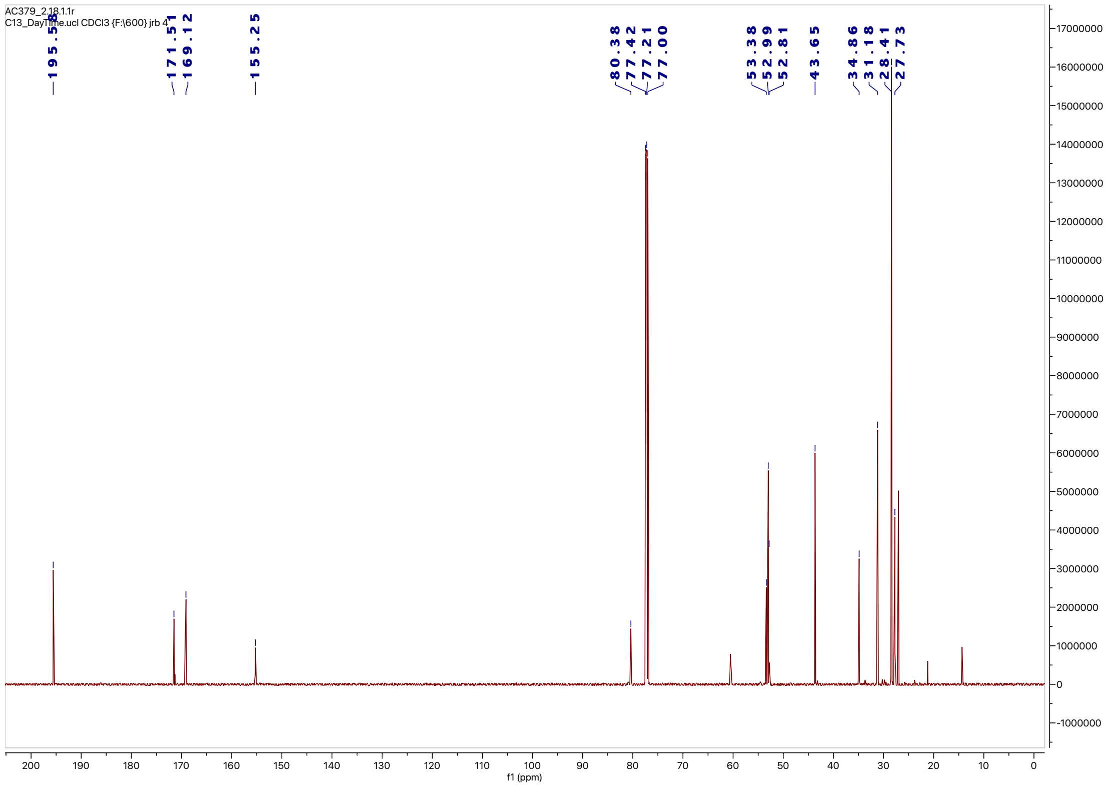

methyl 2-((2-chloro-2-phenylacetyl)thio)acetate (3)

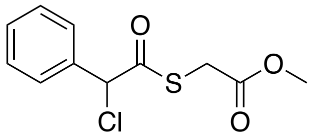

Methyl thioglycolate (0.20 mL, 2.24 mmol, 1.0 eq.) and triethylamine (0.31 mL, 2.24 mmol, 1.0 eq.) were combined in DCM (2.0 mL). The mixture was then added dropwise over 2 h time into a stirring solution of α-chlorophenylacetyl chloride (1.06 mL, 6.72 mmol, 3.0 eq.) in DCM (10.0 mL). The reaction was performed at room temperature, under argon atmosphere and constant agitation. The solvent was then removed *in vacuo* and purification by column chromatography (gradient elution from 100% pet. ether to 30% ethyl acetate in pet. ether) afforded the target compound as light-yellow oil (698 mg, 2.70 mmol, quantitative yield).

^{1}H NMR (600 MHz, $CDCl_3$) δ_H 7.48-7.47 (m, 2H), 7.40-7.38 (m, 3H), 5.50 (s, 1H), 3.70 (s, 5H); ^{13}C (150 MHz, $CDCl_3$) δ_C 194.6 (C), 168.6 (C), 135.5 (CH), 129.7 (CH), 129.2 (CH), 128.4 (CH), 65.7 (CH), 53.1 (CH_3), 32.3 (CH_2); IR (oil) $_{Vmax}$/cm^{-1} 2953, 2844, 1738, 1679; LRMS (ESI) *m/z* (%) 259.0 ([^{35}M+H]$^+$, 100), 261.0 ([^{37}M+H]$^+$, 35); HRMS (ESI) *m/z* calculated for [$C_{11}H_{11}{}^{35}ClO_3S$] 258.0117, observed 258.0197.

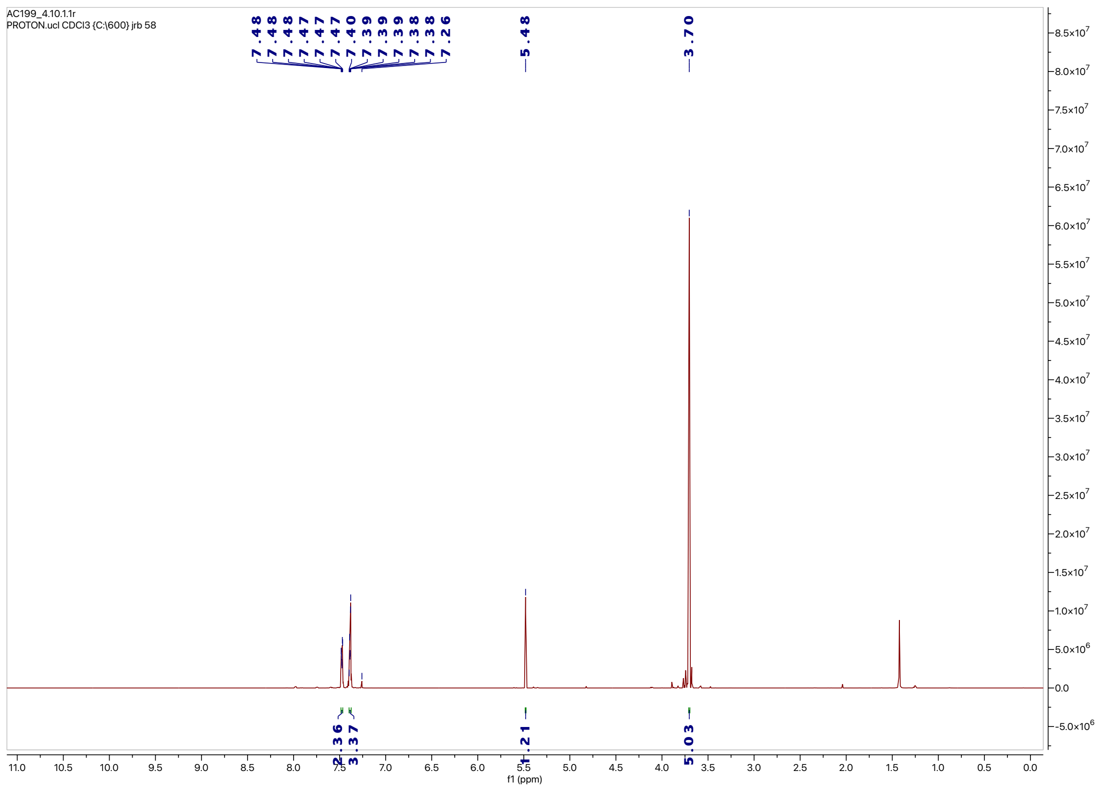

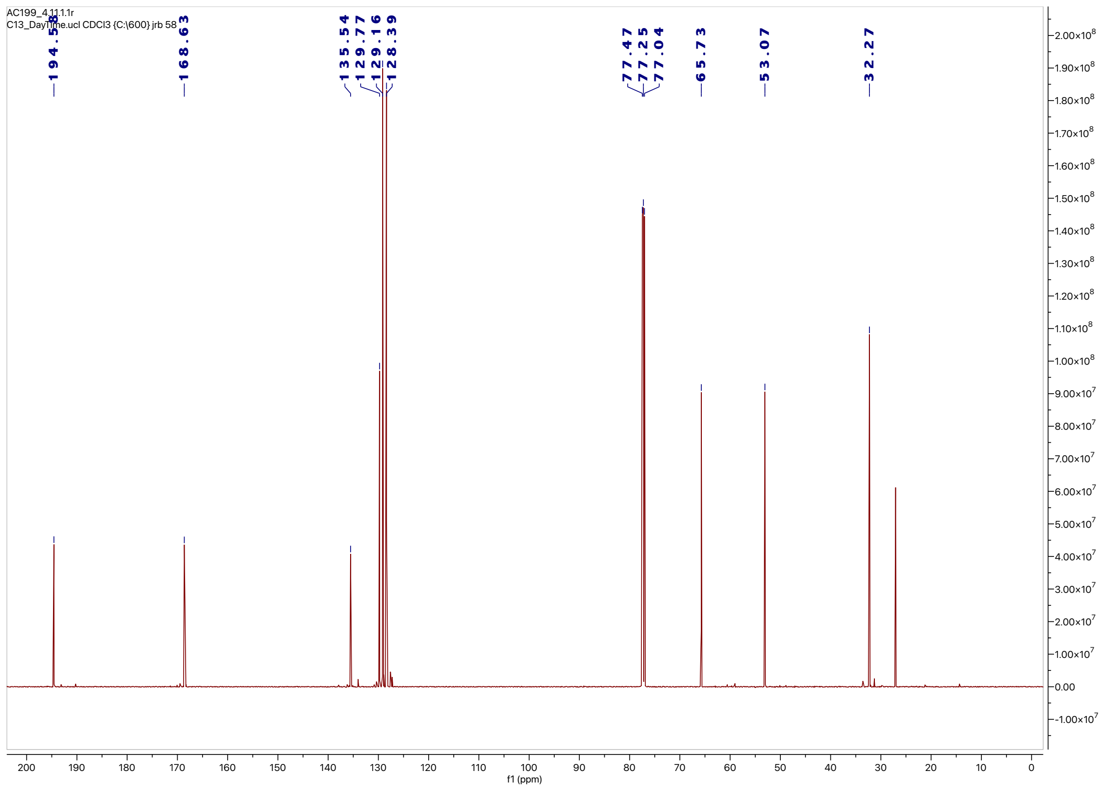

methyl *N*-(*tert*-butoxycarbonyl)-*S*-(2-chloro-2-phenylacetyl)-*L*-cysteinate (S4)

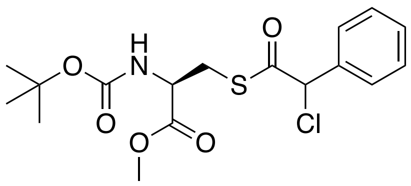

Methyl 2-((2-chloro-2-phenylacetyl)thio)acetate **3** (0.12 g, 0.46 mmol, 1.1 eq.) was dissolved in 0.5 mL of MeCN followed by addition of 0.7 mL of 50 mM Phosphate Buffer pH 6.75 (addition of MeCN will increase pH to 7.4). *N*-Boc cysteine methyl ester (0.09 g, 0.41 mmol, 1.0 eq.) was then added and the mixture was stirred for 3 h at room temperature. The solvent was then removed *in vacuo* and purification by column chromatography (gradient elution from 100% cyclohexane to 40% diethyl ether in cyclohexane) afforded the target compound as clear oil (93.1 mg, 0.24 mmol, 58% yield).

^{1}H NMR (600 MHz, CD_3CN) δ_H 7.49-7.48 (m, 2H), 7.44-7.42 (m, 3H), 5.72 (s, 1H), 4.33-4.32 (m, 1H), 3.65 (s, 3H), 3.46-3.41 and 3.22-3.16 (m, 2H), 1.39 (s, 9H); ^{13}C (150 MHz, CD_3CN) δ_C 195.8 (C), 171.8 (C), 156.2 (C), 137.1 (C), 130.6 (C), 130.1 (C), 129.3 (C), 80.4 (C), 66.3 (CH), 53.7 (CH), 53.2 (CH_3), 32.3 (CH_2), 28.4 (CH_3); IR (oil) V_{max}/cm^{-1} 2977, 2954, 1741, 1694; LRMS (ESI) *m/z* (%) 388.0 ([^{35}M+H]$^+$, 100), 390.0

(^{37}[M+H]$^{+}$, 33), 288.1 ([^{35}M-Boc]$^{+}$, 95), 410.1 [(^{35}M+Na]$^{+}$, 100); HRMS (ESI) *m/z* calculated for [$C_{17}H_{22}{}^{35}ClNO_5S$] 387.0907, observed 387.0979.

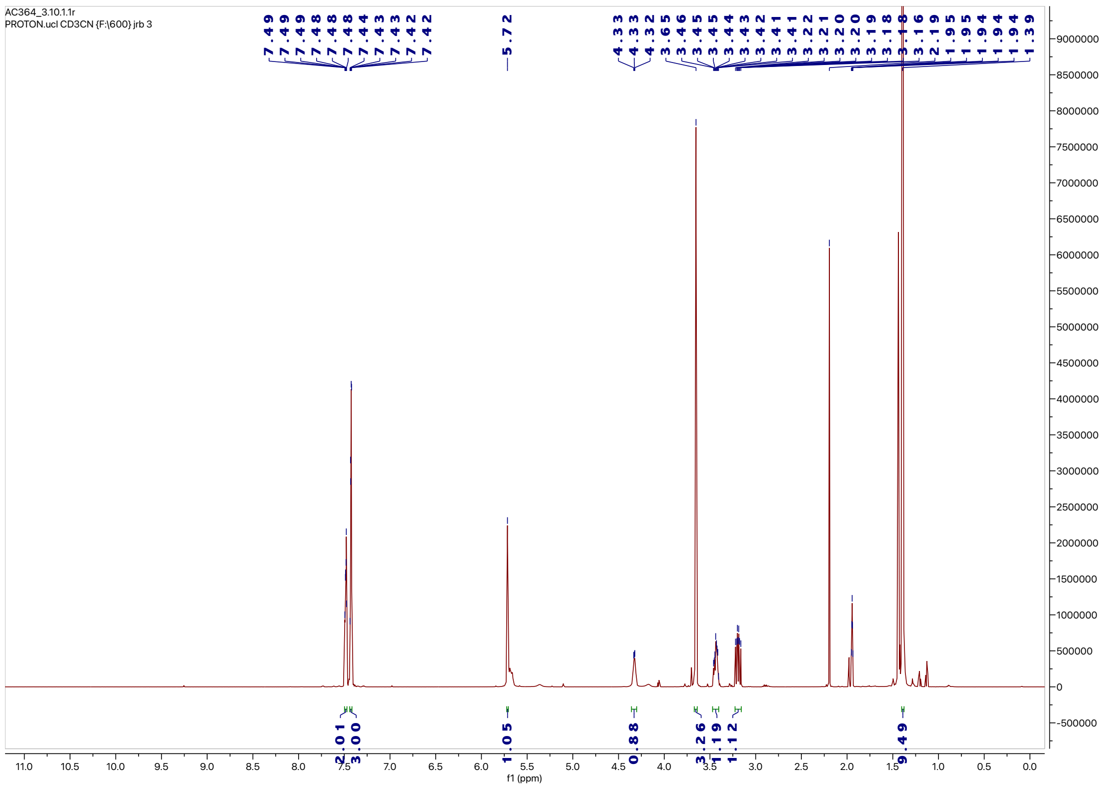

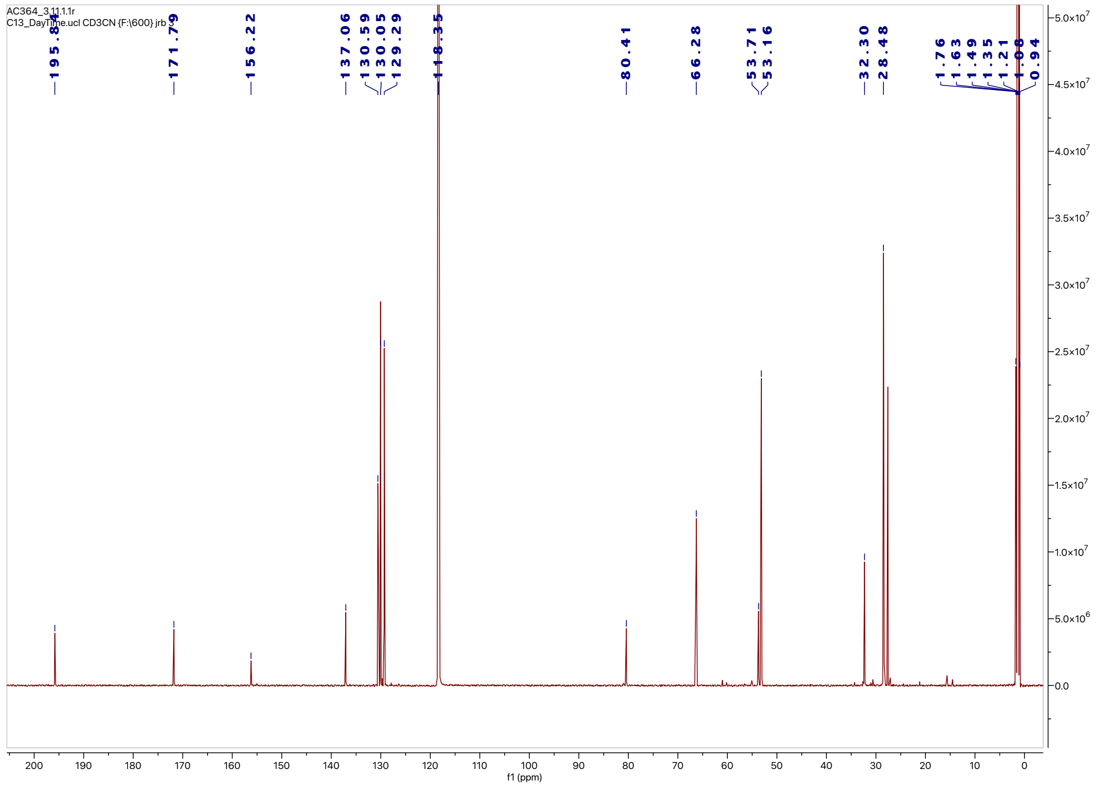

2-fluoro-5-nitrobenzoic acid (S5)

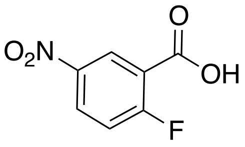

Nitric acid (0.42 mL, 10.1 mmol, 4.0 eq.) was cooled to 0 °C followed by a slow addition of sulphuric acid (0.54 mL, 10.1 mmol, 4.0 eq.). After stirring for 10 min, 2-fluorobenzoyl chloride (0.30 mL, 2.52 mmol, 1.0 eq.) was added and further stirred at 0 °C for 2 h. After this time, the solvent was removed *in vacuo* and the resulting yellow paste was dissolved in EtOAc (15 mL) and washed with H_2O (10 mL). The product was extracted with EtOAc (3 x 10 mL), dried ($MgSO_4$) and the solvent was removed *in vacuo*. The resulting solid was dry loaded on silica and purified by flash chromatography (gradient elution from 100% cyclohexane to 50% EtOAc in cyclohexane and 1% AcOH) to afford the target compound as white powder (413 mg, 2.23 mmol, 89% yield).

^{1}H NMR (600 MHz, MeOD) δ_H 8.79-8.77 (m, 1H), 8.49-8.46 (m, 1H), 7.47 (t, 1H, *J* = 9.0 Hz); ^{13}C (150 MHz, MeOD) δ_C 167.4 and 165.6 (C), 165.1 and 165.0 (C), 145.4 (C), 130.8 and 130.7 (CH), 129.1 and 129.0 (CH), 121.7 and 121.6 (C), 119.8 and 119.6 (CH); IR (solid) V_{max}/cm^{-1} 2929, 2858, 1657; LRMS (ESI) *m/z* (%) 186.0 ([M+H]$^+$, 100); HRMS (ESI) *m/z* calculated for [$C_7H_4FNO_4$] 185.0124, observed 185.0195.

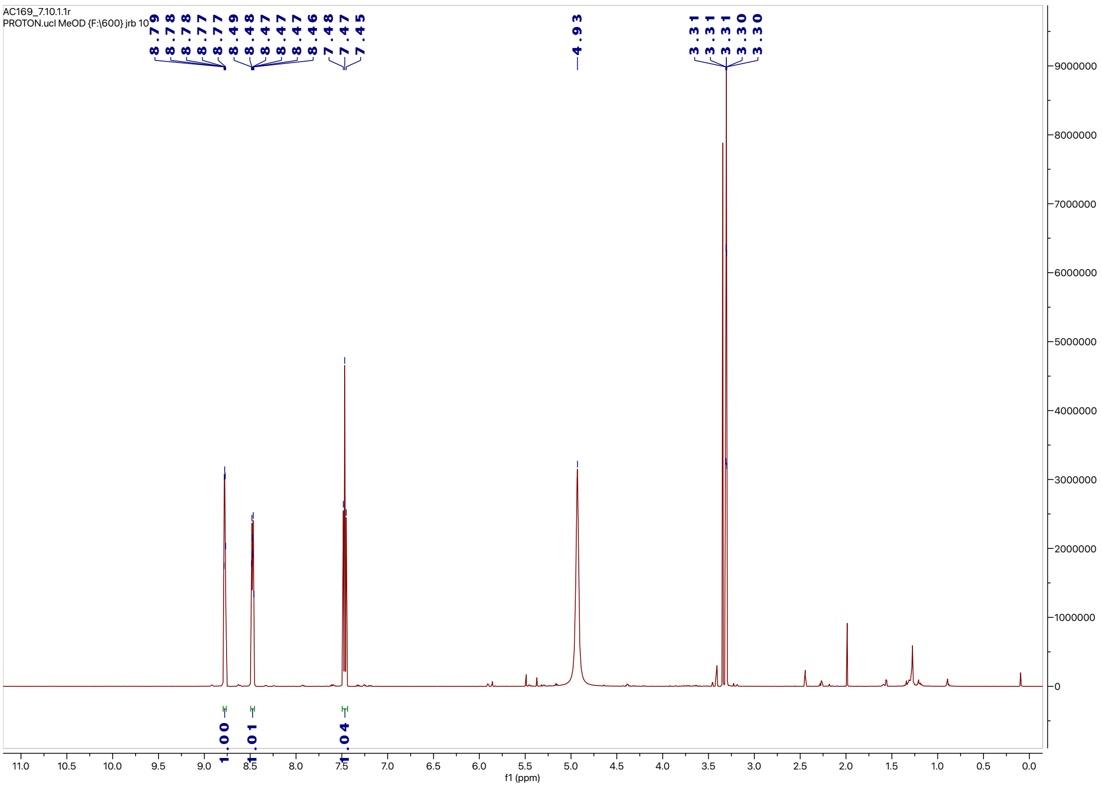

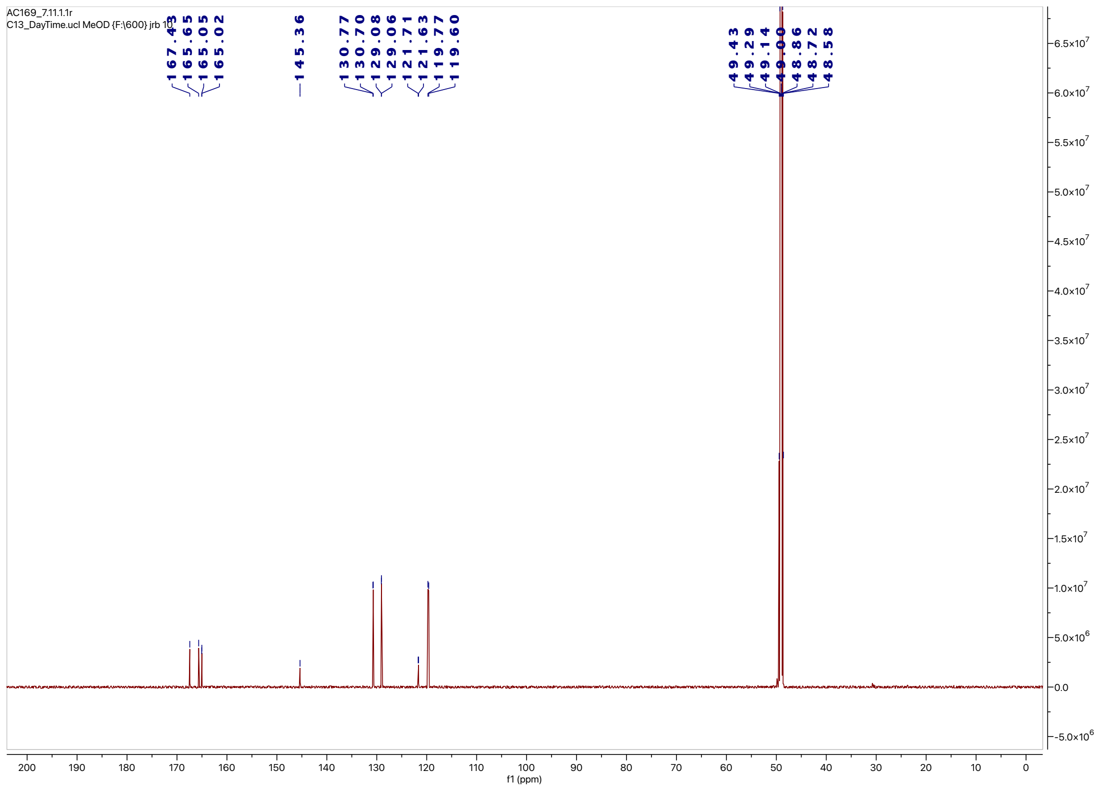

methyl 2-((2-fluoro-5-nitrobenzoyl)thio)acetate (4)

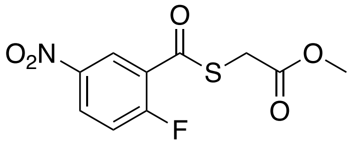

2-fluoro-5-nitrobenzoic acid (**S5**) (0.20 g, 1.08 mmol, 1.1 eq.) was dissolved in 5 mL of DMF followed by addition of N-Ethoxycarbonyl-2-ethoxy-1,2-dihydroquinoline (0.29 g, 1.19 mmol, 1.2 eq.), this was stirred at room temperature for 30 min. Lastly, methyl thioglycolate (0.09 mL, 0.97 mmol, 1.0 eq.) was added and the mixture was left stirring O/N at 80 °C. The solvent was then removed *in vacuo* and purification by column chromatography (gradient elution from 100% DCM to 10% methanol in DCM) afforded the target compound as yellow oil (205 mg, 0.75 mmol, 77%).

^{1}H NMR (600 MHz, $CDCl_3$) δ_H 8.79-8.78 (m, 1H), 8.45-8.42 (m, 1H), 7.38 (t, 1H, *J* = 9.2 Hz), 3.92 (s, 2H), 3.79 (s, 3H); ^{13}C (150 MHz, $CDCl_3$) δ_C 185.3 and 185.3 (C), 168.6 and 164.8 (C), 163.0 (C), 144.3 (C), 129.9 and 129.8 (C), 126.2 and 126.1 (CH), 125.4 and 125.3 (CH), 118.7 and 118.5 (CH), 53.22 (CH_3), 31.9 and 31.8 (CH_2); IR (oil) V_{max}/cm^{-1} 3105, 3076, 2964, 1744, 1707; LRMS (ESI) *m/z* (%) 274.0 ([M + H]$^+$, 100); HRMS (ESI) *m/z* calculated for [$C_{10}H_8FNO_5S$] 273.0107, observed 273.0175.

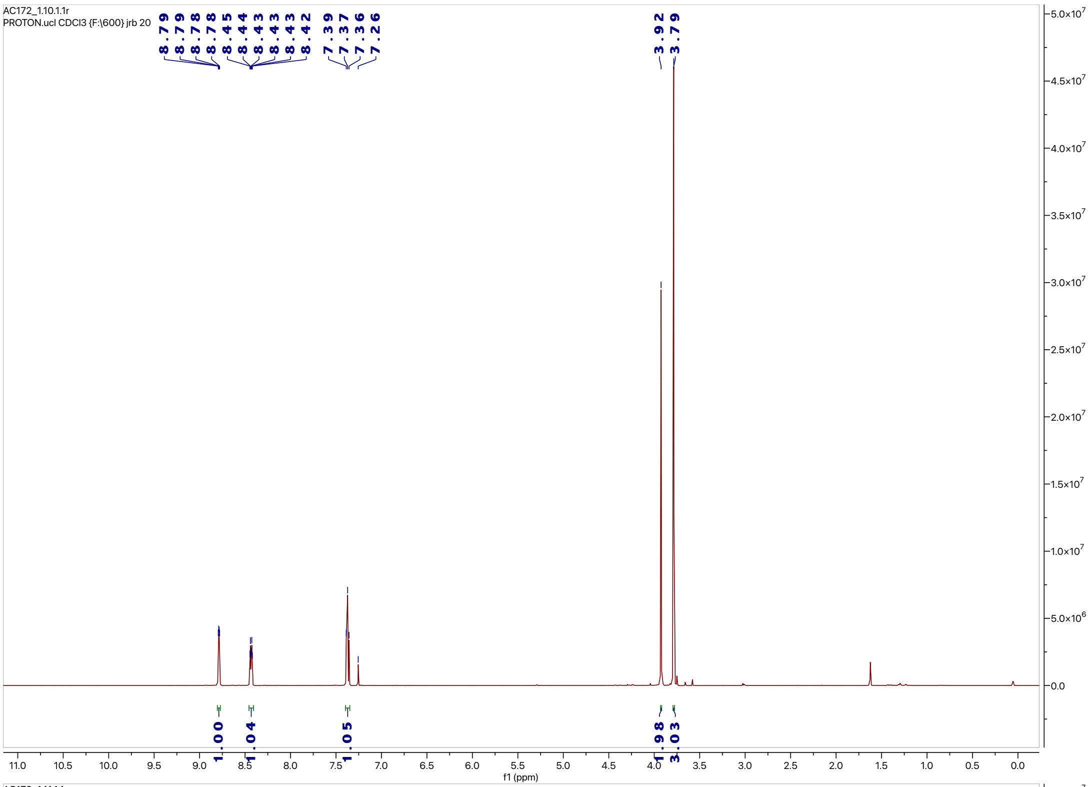
AC172_1.10.1.1r
PROTON.ucl CDCl3 {F:\600} jrb 20
8.79 8.79 8.78 8.78 8.45 8.44 8.43 8.43 8.43 8.42 7.39 7.37 7.36 7.26
3.92 3.79
1.00 1.04 1.05 1.98 3.03
f1 (ppm)

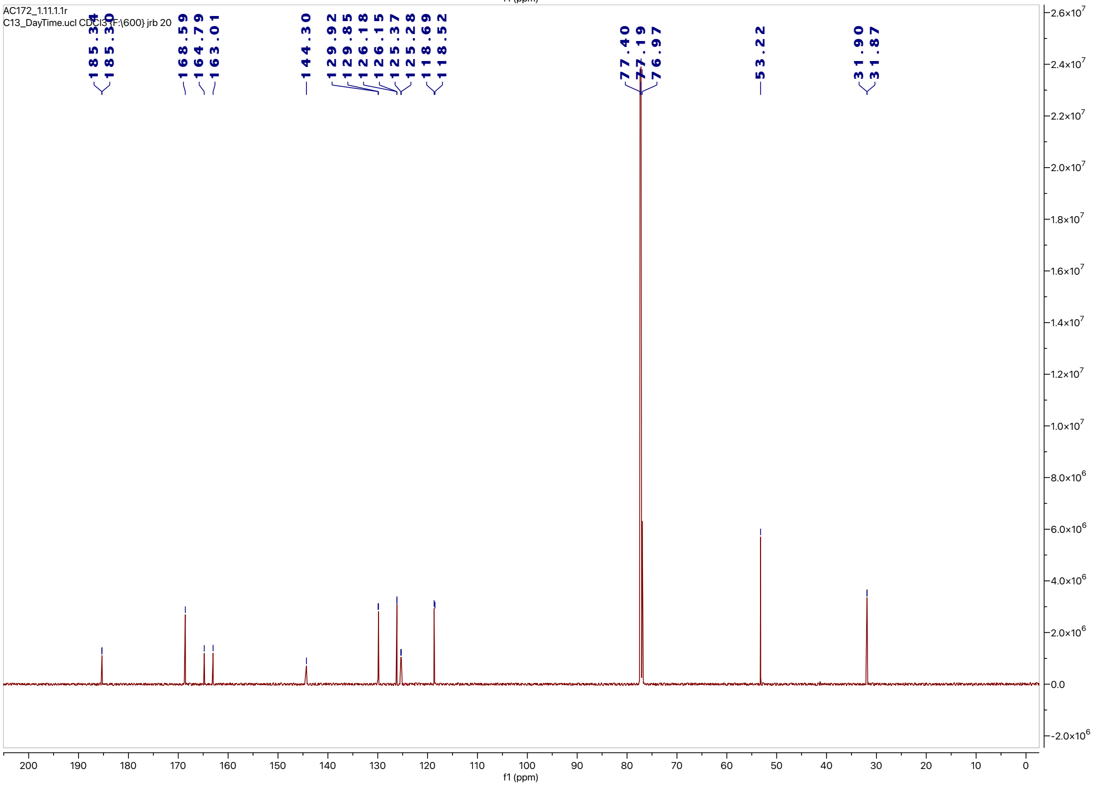
AC172_1.11.1.1r
C13_DayTime.ucl CDCl3 {F:\600} jrb 20
185.34 185.30 168.59 164.79 163.01 144.30 129.92 129.85 126.18 126.15 125.37 125.28 118.69 118.52
77.40 77.19 76.97 53.22 31.90 31.87
f1 (ppm)

methyl *N*-(*tert*-butoxycarbonyl)-*S*-(2-fluoro-5-nitrobenzoyl)-*D*-cysteinate (S6)

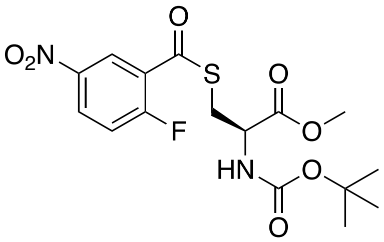

Methyl 2-((2-fluoro-5-nitrobenzoyl)thio)acetate **4** (0.06 g, 0.20 mmol, 1.1 eq.) was dissolved in 0.5 mL of MeCN followed by addition of 0.7 mL of 50 mM Phosphate Buffer pH 6.75 (addition of MeCN will increase pH to 7.4). *N*-Boc cysteine methyl ester (0.04 g, 0.18 mmol, 1.0 eq.) was then added and the mixture was stirred for 3 h at room temperature. The solvent was then removed *in vacuo* and purification by column chromatography (gradient elution from 100% cyclohexane to 40% ethyl acetate in cyclohexane) afforded the target compound as yellow oil (45.0 mg, 0.11 mmol, 62% yield).

^{1}H NMR (600 MHz, $CDCl_3$) δ_H 8.72-8.70 (m, 1H), 8.42-8.39 (m, 1H), 7.34 (t, 1H, *J* = 9.1 Hz), 5.32 (s, 1H), 4.67-4.63 (m, 1H), 3.78 (s, 3H), 3.67-3.64 and 3.53-3.48 (m, 2H), 1.42 (s, 9H); ^{13}C (150 MHz, $CDCl_3$) δ_C 186.3 and 186.2 (C), 170.9 (C), 164.4 and 162.6 (C), 155.2 (C), 144.2 (C), 129.7 and 129.6 (CH), 126.1 and 126.0 (CH), 118.6 and 118.5 (CH), 80.6 (C), 53.1 (CH_3), 52.8 (CH), 32.1 (CH_2), 28.4 (CH_3); IR (yellow oil) V_{max}/cm^{-1} 3101, 3076, 2964, 1742, 1690, 1642; LRMS (ESI) *m/z* (%) 403.0 ([M + H]$^+$, 100); HRMS (ESI) *m/z* calculated for [$C_{16}H_{19}FN_2O_7S$] 402.0897, observed 402.0977.

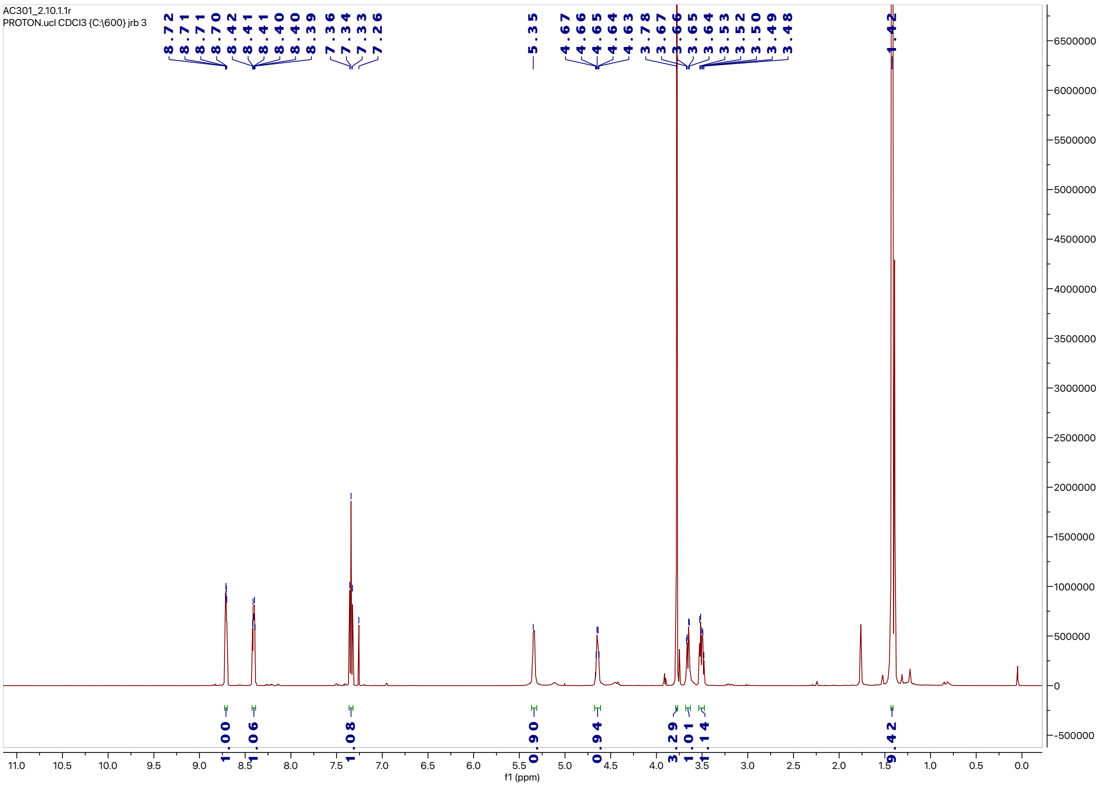
AC301_2.10.1.1r
PROTON.ucl CDCl3 {C:\600} jrb 3
8.72 8.71 8.71 8.70 8.42 8.41 8.41 8.40 8.40 8.39 7.36 7.34 7.33 7.26
5.35
4.67 4.66 4.65 4.64 4.63 3.78 3.67 3.66 3.65 3.64 3.53 3.52 3.50 3.49 3.48
1.42
1.00 1.06 1.08 0.90 0.94 3.29 1.01 1.14 9.42
f1 (ppm)

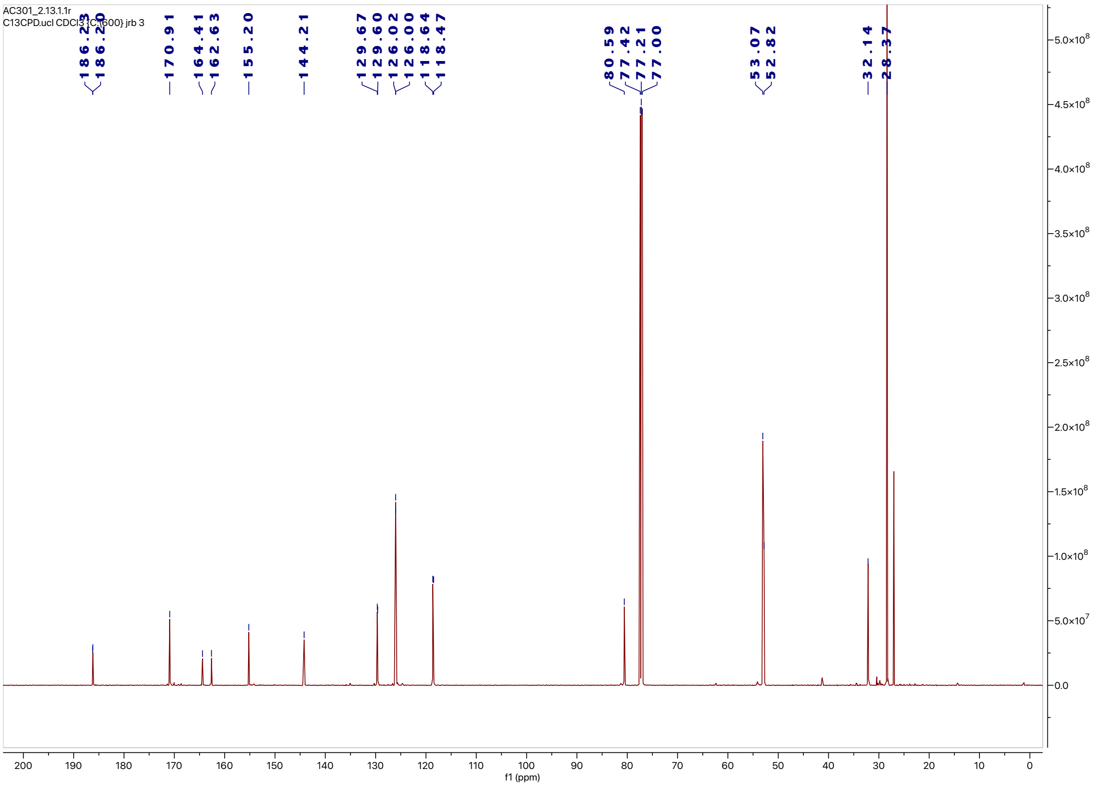
AC301_2.13.1.1r
C13CPD.ucl CDCl3 {C:\600} jrb 3
186.23 186.20
170.91 164.41 162.63 155.20 144.21
129.67 129.60 126.02 126.00 118.64 118.47
80.59 77.42 77.21 77.00
53.07 52.82
32.14 28.37
f1 (ppm)

pent-4-yne-1-thiol[1] (S7)

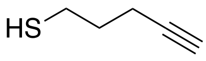

Methanesulfonylchloride (0.87 mL, 11.3 mmol, 1.5 eq.) was added over 5 min at 0 °C to a stirred solution of 4-pentyn-1-ol (0.70 mL, 7.5 mmol, 1.0 eq.) and triethylamine (1.34 mL, 15.0 mmol, 2.0 eq.) in DCM. After 5 min at 0 °C the reaction was warmed up to room temperature and stirred for 2 h. This was then washed with water (2 x 10 mL) and brine (1 x 10 mL), dried over $MgSO_4$, and concentrated *in vacuo*. The crude sulfonate was used without purification, and it was dissolved in DMF (47 mL), to that potassium thioacetate (0.90 g, 7.87 mmol, 1.05 eq.) was added. The reaction mixture was heated up to 50 °C for 20 h, then diluted with DCM (40 mL). DMF was removed by repeated washing with water (4 x 10 mL). The combined organics were concentrated *in vacuo*.
Alongside that, a suspension of K_2CO_3 (0.38 g, 2.75 mmol, 1.1 eq.) in MeOH (15 mL) was stirred for 20 min at room temperature. This was then added to the main crude thioacetate. The evolution of reaction was monitored by TLC. After 2 h, the reaction mixture was quenched with 1 M HCl until pH 2 was obtained and extracted with DCM (3 x 10 mL). The organic layer was washed with aq. NaCl, dried over $MgSO_4$, filtered, and concentrated *in vacuo*. The crude product was then purified using column chromatography (gradient elution from 100% cyclohexane to 20% ethyl acetate in cyclohexane) afforded the target compound as light-yellow oil (179 mg, 1.79 mmol, 24% yield).

^1H NMR (600 MHz, $CDCl_3$) δ_H 2.79 (t, 2H, *J* = 7.0 Hz), 2.33 (dt, 2H, *J* = 6.9, 2.7 Hz), 1.97 (t, 1H, *J* = 2.6 Hz), 1.92 (quin, 2H, *J* = 7.4 Hz); ^{13}C (150 MHz, $CDCl_3$) δ_C 83.4 (C), 69.3 (CH), 37.5 (CH_2), 27.8 (CH_2), 17.3 (CH_2); IR (oil) V_{max}/cm^{-1} 3292, 2194; LRMS (ESI) *m/z* (%) 101.0 ($[M + H]^+$, 100); HRMS (ESI) *m/z* calculated for [C_5H_8S] 100.0346, observed 100.0419.

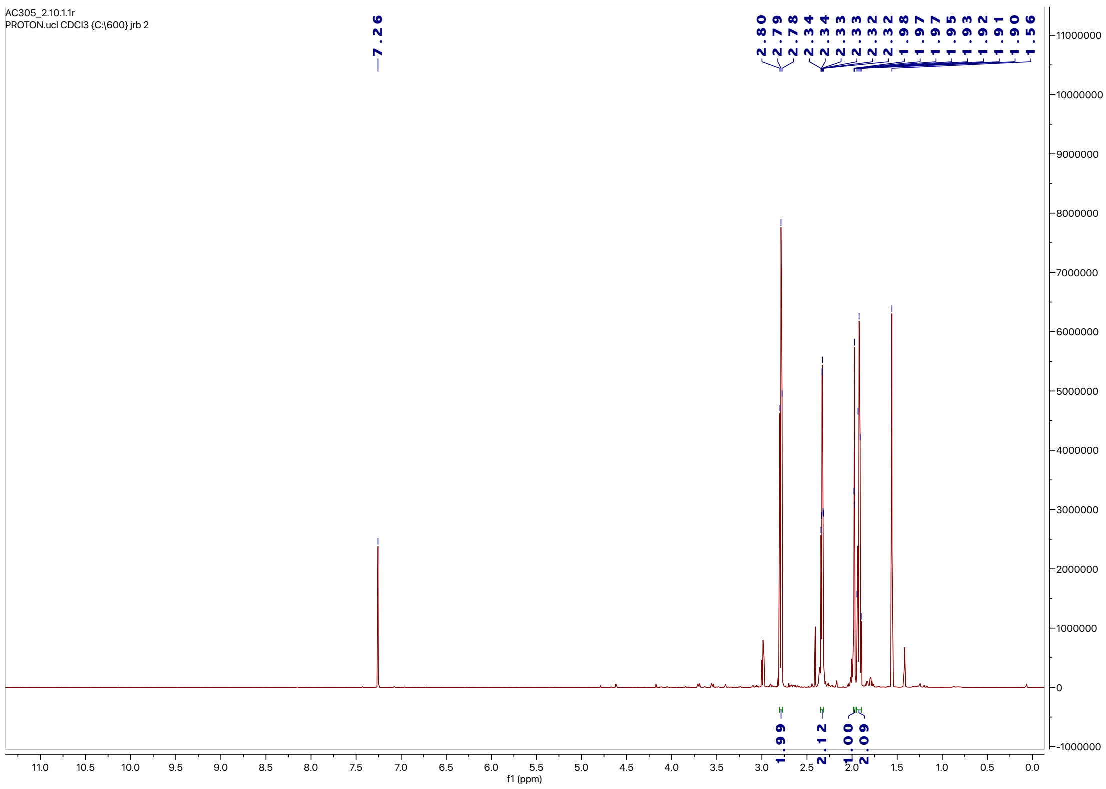
AC305_2.10.1.1r
PROTON.ucl CDCl3 {C:\600} jrb 2
7.26
2.80
2.79
2.78
2.34
2.34
2.33
2.33
2.32
2.32
1.98
1.97
1.97
1.95
1.93
1.92
1.91
1.90
1.56
1.99
2.12
1.00
2.09
f1 (ppm)

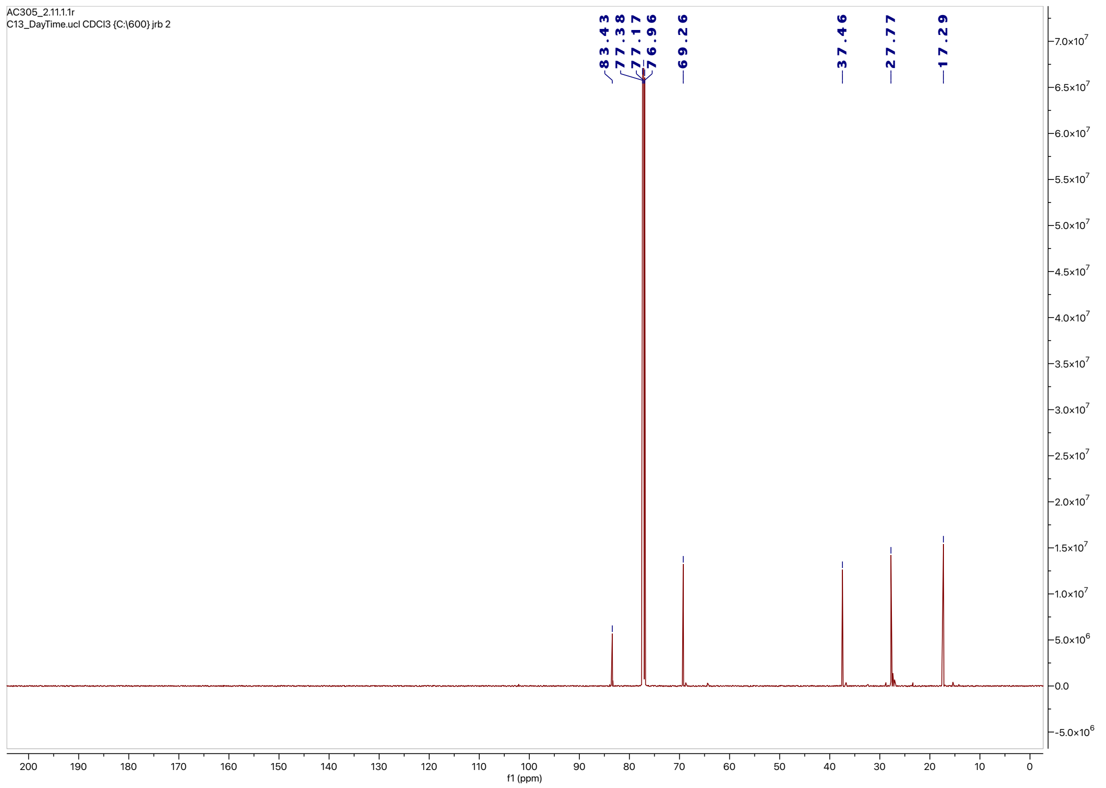
AC305_2.11.1.1r
C13_DayTime.ucl CDCl3 {C:\600} jrb 2
83.43
77.38
77.17
76.96
69.26
37.46
27.77
17.29
f1 (ppm)

2-(pent-4-yn-1-yldisulfaneyl)pyridine (S8)

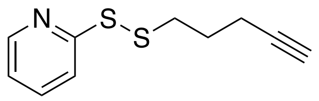

2,2’-dipyridyl disulfide (0.08 g, 0.38 mmol, 1.25 eq.) was dissolved in 5 mL of cold THF followed by addition of triethylamine (0.04 mL, 0.46 mmol, 1.5 eq.). To the cold mixture, pent-4-yne-1-thiol (**S7**) (0.03 g, 0.31 mmol, 1.0 eq.) was added and the ice bath was removed. The mixture was stirred at room temperature for 2 h. The solvent was then removed *in vacuo* and purification by column chromatography (gradient elution from 100% cyclohexane to 30% ethyl acetate in cyclohexane) afforded the target compound as light-yellow oil (15 mg, 0.07 mmol, 23% yield).

^{1}H NMR (600 MHz, $CDCl_3$) δ_H 8.47 (d, 1H, *J* = 5.0 Hz), 7.71 (d, 1H, *J* = 8.2 Hz), 7.64 (td, 1H, *J* = 7.7, 1.9 Hz), 7.10-7.08 (m, 1H), 2.91 (t, 2H, *J* = 7.2 Hz), 2.33 (dt, 2H, *J* = 6.9, 2.7 Hz), 1.95 (t, 1H, *J* = 2.7 Hz), 1.93 (q, 2H, *J* = 6.9 Hz); ^{13}C (150 MHz, $CDCl_3$) δ_C 160.4 (C), 149.8 (CH), 137.2 (CH), 120.8 (CH), 119.8 (CH), 83.2 (C), 69.4 (CH), 37.5 (CH_2), 27.5 (CH_2), 17.3 (CH_2); IR (oil) V_{max}/cm^{-1} 2926, 2116, 1972; LRMS (ESI) *m/z* (%) 210.0 ([M + H]$^+$, 100); HRMS (ESI) *m/z* calculated for [$C_{10}H_{11}NS_2$] 209.0332, observed 209.0410.

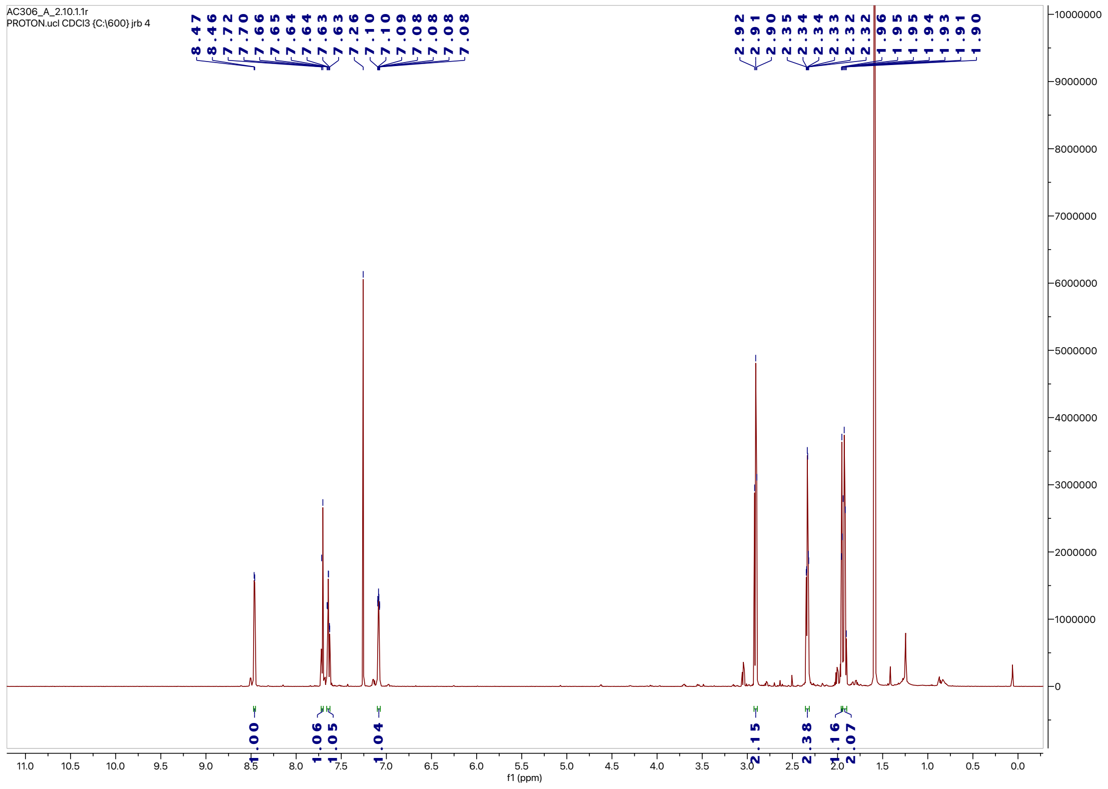

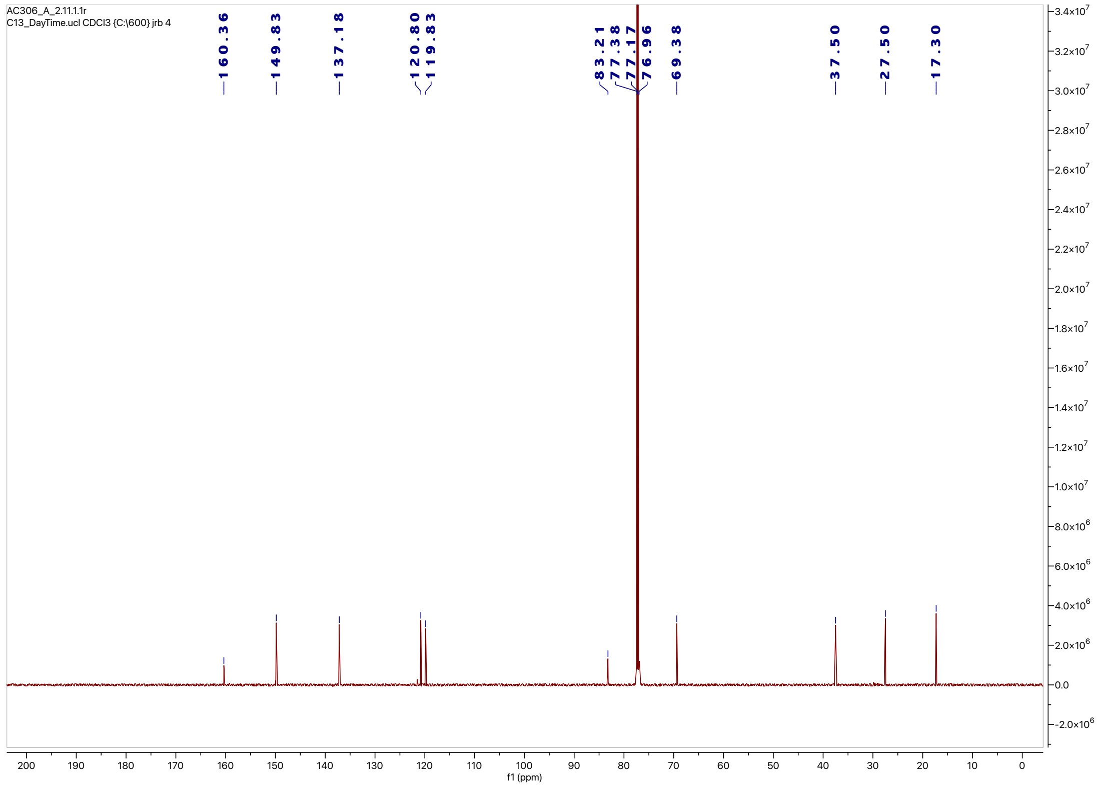

tert-butyl (2-(2-(2-aminoethoxy)ethoxy)ethyl)carbamate[2] (S9)

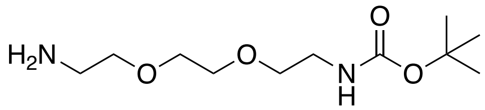

2,2'-(ethane-1,2-diylbis(oxy))bis(ethan-1-amine) (0.40 mL, 2.69 mmol, 5.0 eq.) was dissolved in 10 mL of DCM over ice, followed by slow addition of di-tert-butyl-dicarbonate (0.12 g, 0.54 mmol, 1.0 eq.) separately dissolved in 5 mL of DCM. The mixture was left for 16 h, stirring. The crude was then washed with water (1 x 5 mL) and extracted with DCM (2 x 5 mL), dried over $MgSO_4$, and concentrated *in vacuo* that afforded the target compound as light-yellow oil (142 mg, 0.57 mmol, quantitative yield).

^{1}H NMR (600 MHz, $CDCl_3$) δ_H 5.17 (s, 1H), 3.59 (s, 4H), 3.52 (t, 2H, *J* = 5.2 Hz), 3.49 (t, 2H, *J* = 5.2 Hz), 3.29 (d, 2H, *J* = 5.1 Hz), 2.85 (t, 2H, *J* = 5.7 Hz), 1.41 (s, 9H); ^{13}C (150 MHz, $CDCl_3$) δ_C 156.2 (C), 79.3 (C), 73.6 (CH_2), 70.4 (CH_2), 70.3 (CH_2), 41.9 (CH_2), 40.4 (CH_2), 28.5 (CH_3); IR (oil) V_{max}/cm^{-1} 2924, 1690; LRMS (ESI) *m/z* (%) 249.2 ([M + H]$^+$, 100); HRMS (ESI) *m/z* calculated for [$C_{11}H_{24}N_2O_4$] 248.1736, observed 248.1808.

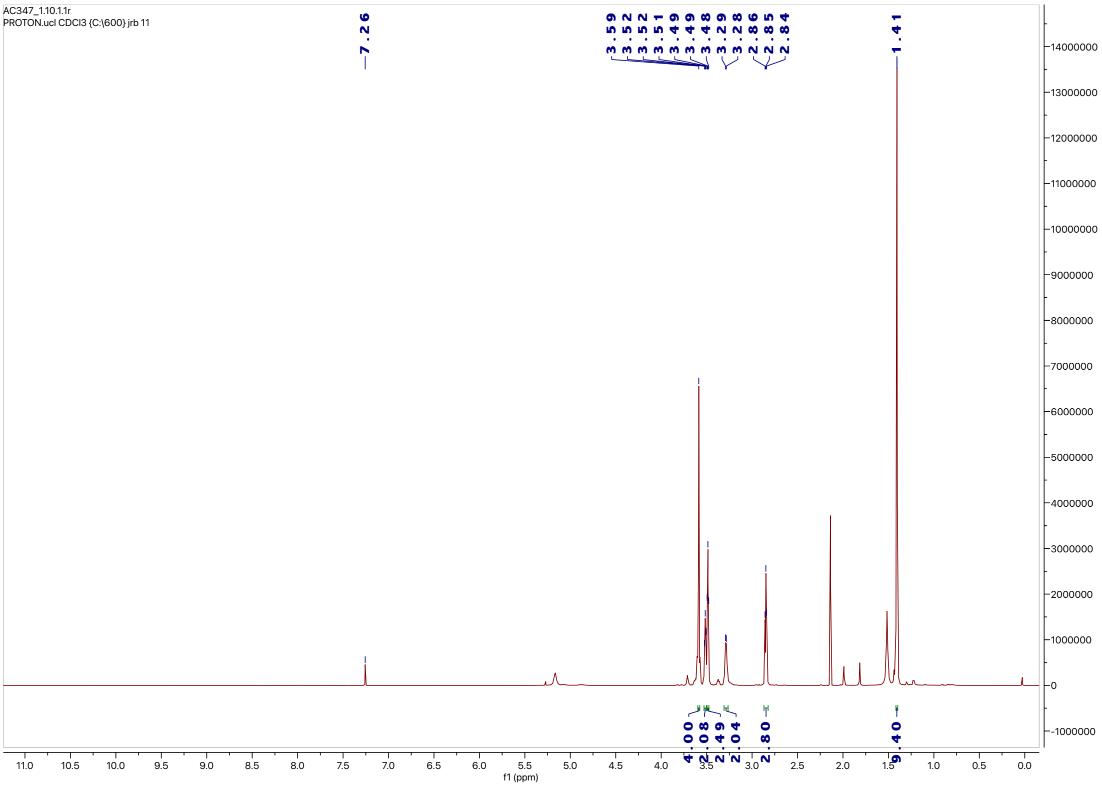
AC347_1.10.1.1r
PROTON.ucl CDCl3 {C:\600} jrb 11
7.26
3.59
3.52
3.52
3.51
3.49
3.48
3.29
3.28
2.86
2.85
2.84
1.41
4.00
2.08
2.49
2.04
2.80
9.40
f1 (ppm)

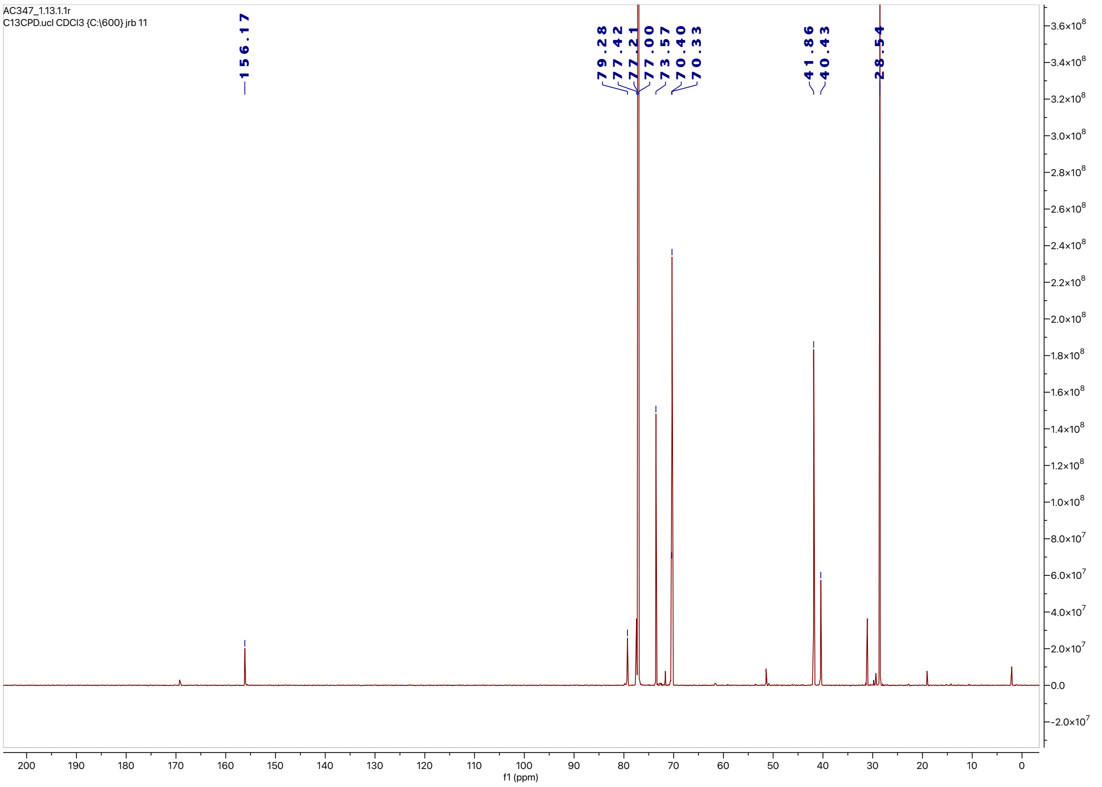
AC347_1.13.1.1r
C13CPD.ucl CDCl3 {C:\600} jrb 11
156.17
79.28
77.42
77.21
77.00
73.57
70.40
70.33
41.86
40.43
28.54
f1 (ppm)

tert-butyl (2-(2-(2-(5-((3a*S*,4*S*,6a*R*)-2-oxohexahydro-1*H*-thieno[3,4-*d*]imidazol-4-yl)pentanamido)ethoxy)ethoxy)ethyl)carbamate[2] (S10)

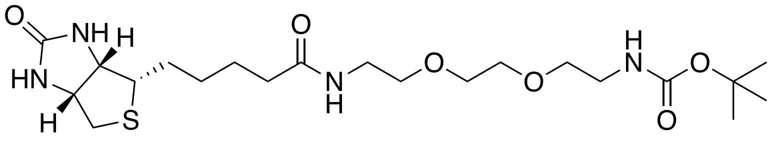

Biotin (0.14 g, 0.57 mmol, 1.0 eq.) was first dissolved in 2 mL DMF, followed by addition of EDC.HCl (0.16 g, 0.86 mmol, 1.5 eq.), HOBt (0.12 g, 0.86 mmol, 1.5 eq.) and triethylamine (0.24 mL, 1.72 mmol, 3.0 eq.). This was stirred for 30 min at room temperature. Then PEG-Boc (**S9**) (*tert*-butyl (2-(2-(2-aminoethoxy)ethoxy)ethyl)carbamate) was added, and the mixture was stirred for 16 h at room temperature. The solvent was then removed *in vacuo* and purification by column chromatography (gradient elution from 100% DCM to 30% methanol in DCM) afforded the target compound as white wax (106 mg, 0.22 mmol, 39% yield*). *Minor impurities observed in the aromatic region of the NMR, this was taken directly to the next step.

^{1}H NMR (700 MHz, MeOD) δ_H 4.50-4.48 (m, 1H), 4.32-4.29 (m, 1H), 3.61 (s, 4H), 3.54 (t, 2H, *J* = 5.6 Hz), 3.51 (t, 2H, *J* = 5.7 Hz), 3.36 (t, 2H, *J* = 5.6 Hz), 3.22 (t, 2H, *J* = 5.7 Hz), 3.20-3.19 (m, 1H), 2.92 (dd, 1H, *J* = 12.9, 4.9 Hz), 2.71 (d, 1H, *J* = 12.9 Hz), 2.22 (t, 2H, *J* = 7.4 Hz), 1.76-1.56 (m, 6H), 1.43 (s, 9H); ^{13}C (150 MHz, MeOD) δ_C 176.0 (C), 166.0 (C), 158.3 (C), 80.0 (C), 71.2 (CH_2), 71.0 (CH_2), 70.5 (CH_2), 63.3 (CH), 61.5 (CH), 56.9 (CH), 41.2 (CH_2), 41.0 (CH_2), 40.2 (CH_2), 36.7 (CH_2), 29.7 (CH_2), 29.4 (CH_2), 28.7 (CH_3), 26.8 (CH_2); IR (gum) V_{max}/cm^{-1} 2932, 2867, 1690; LRMS (ESI) *m/z* (%) 475.2 ([M + H]$^+$, 100); HRMS (ESI) *m/z* calculated for [$C_{21}H_{38}N_4O_6S$] 474.2512, observed 474.2586.

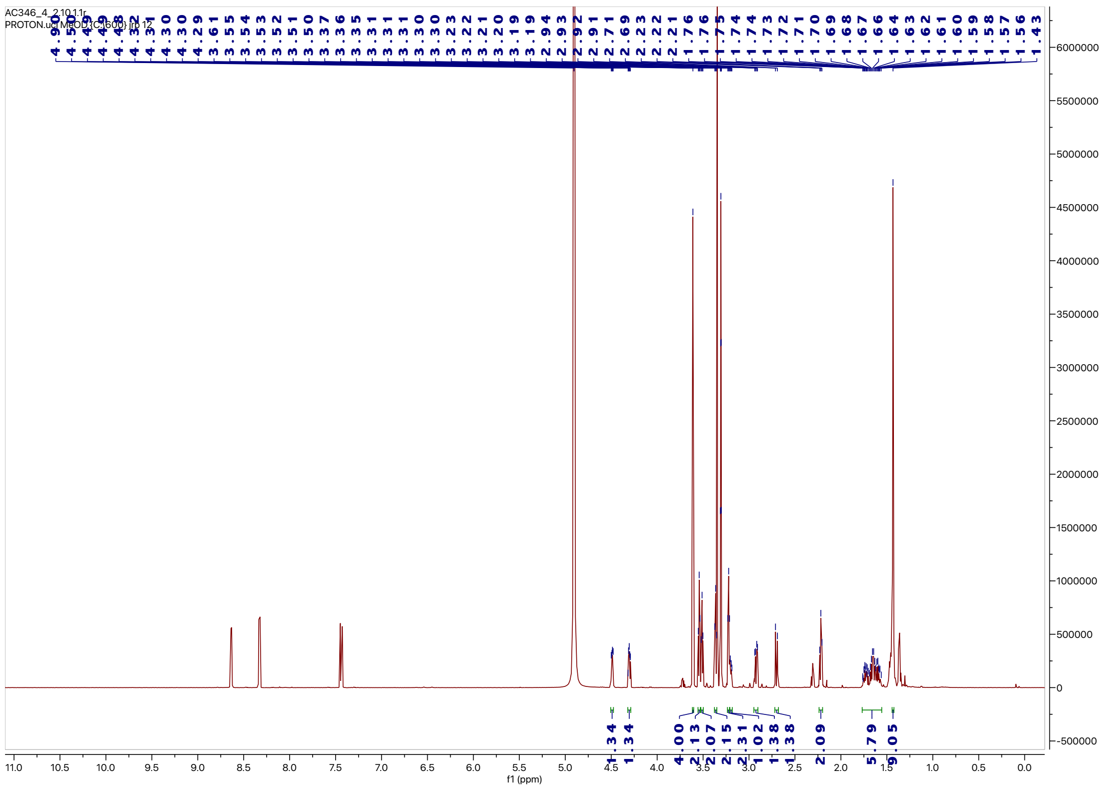

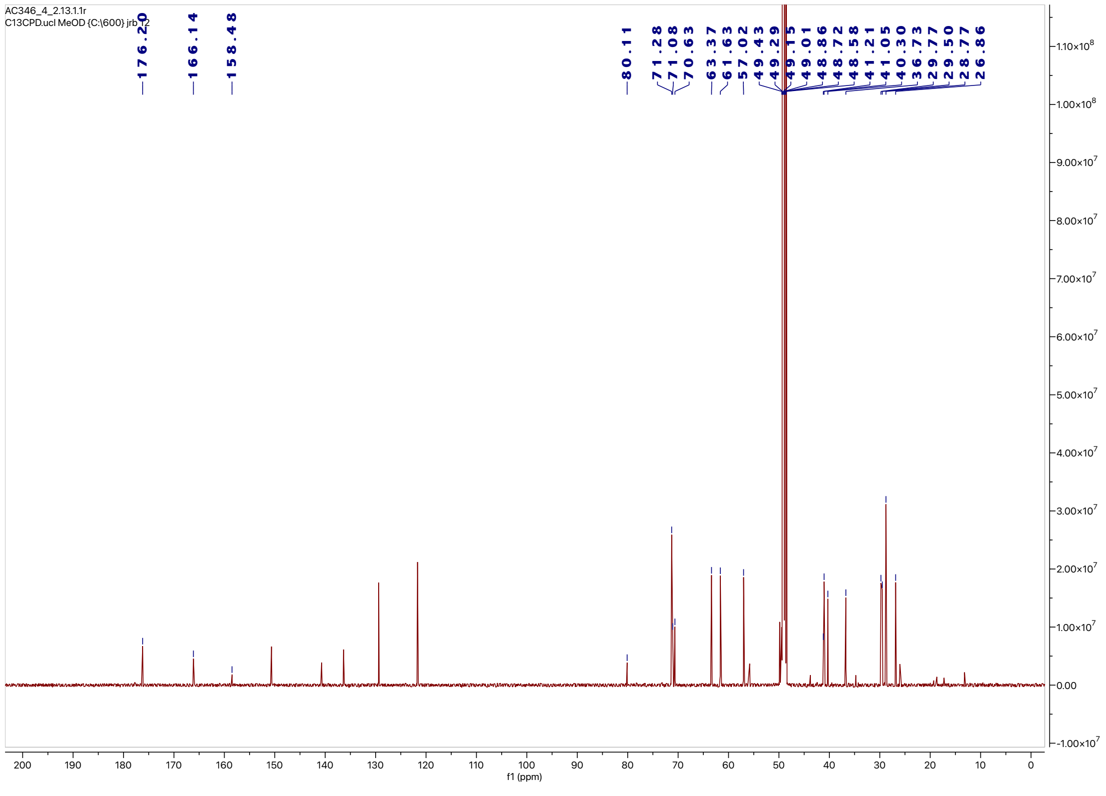

4-formyl-*N*-(2-(2-(2-(5-((3a*R*,4*R*,6a*S*)-2-oxohexahydro-1*H*-thieno[3,4-*d*]imidazol-4-yl)pentanamido)ethoxy)ethoxy)ethyl)benzamide[2] (S11)

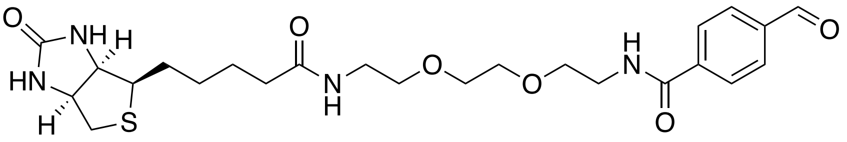

Impure Biotin-PEG-Boc from the previous step (**S10**) (0.06 g, 0.12 mmol, 1.0 eq.) was dissolved in 1 mL of DCM followed by addition of 1 mL of TFA, this was stirred at room temperature for 2 h. After 2 h the solvent was removed, and the mixture was washed with 3 x 5 mL of ethyl acetate to remove the TFA. In a separate flask, 4-formylbenzoic acid (0.04 g, 0.26 mmol, 2.1 eq.) was dissolved in 0.5 mL of MeCN and 0.5 mL DMF. EDC.HCl (0.05 g, 0.27 mmol, 2.2 eq.) and DIPEA (0.05 mL, 0.31 mmol, 2.5 eq.) were added and this was stirred for 30 min at room temperature. Lastly the acid mixture was added to the deprotected Biotin-PEG-NH_2 and the reaction was stirred for 16 h at room temperature. The solvent was then removed *in vacuo* and purification by column chromatography (gradient elution from 100% DCM to 30% methanol in DCM) afforded the target compound white gum (35.8 mg, 0.07 mmol, 57% yield*). *Minor impurities observed in the NMR, however, the purity was found to be sufficient for protein labelling.

^{1}H NMR (600 MHz, DMSO-d) δ_H 10.08 (s, 1H), 8.02 (d, 2H, *J* = 8.4 Hz), 8.00 (d, 2H, *J* = 8.4 Hz), 4.30-4.28 (m, 1H), 4.13-4.10 (m, 1H), 3.54-3.53 (m, 4H), 3.51-3.50 (m, 2H), 3.45-3.43 (m, 2H), 3.18-3.15 (m, 2H), 2.96 (t, 2H, *J* = 6.6 Hz), 2.80 (dd, 1H, *J* = 12.7, 5.2 Hz), 2.58 (d, 1H, *J* = 12.7 Hz), 2.43 (d, 1H, *J* = 7.2 Hz), 2.05 (t, 2H, *J* = 7.7 Hz), 1.51-1.42 (m, 4H), 1.30-1.24 (m, 2H); ^{13}C (150 MHz, DMSO-d) δ_C 193.0 (C), 172.2 (C), 165.5 (*C*), 162.8 (C), 139.4 (C), 135.7 (C), 129.5 (CH), 128.0 (CHCH), 69.6 (CH_2), 69.2 (CH_2), 68.8 (CH_2), 61.1 (CH), 59.2 (CH), 55.5 (CH), 40.0 (CH_2), 38.4 (CH_2), 38.3 (CH_2), 35.1 (CH_2), 28.2 (CH_2), 28.1 (CH_2), 25.3 (CH_2); IR (gum) V_{max}/cm^{-1} 2924, 2825, 1668; LRMS (ESI) *m/z* (%) 507.2 ($[M + H]^+$, 100); HRMS (ESI) *m/z* calculated for [$C_{24}H_{34}N_4O_6S$] 506.2127, observed 507.2270.

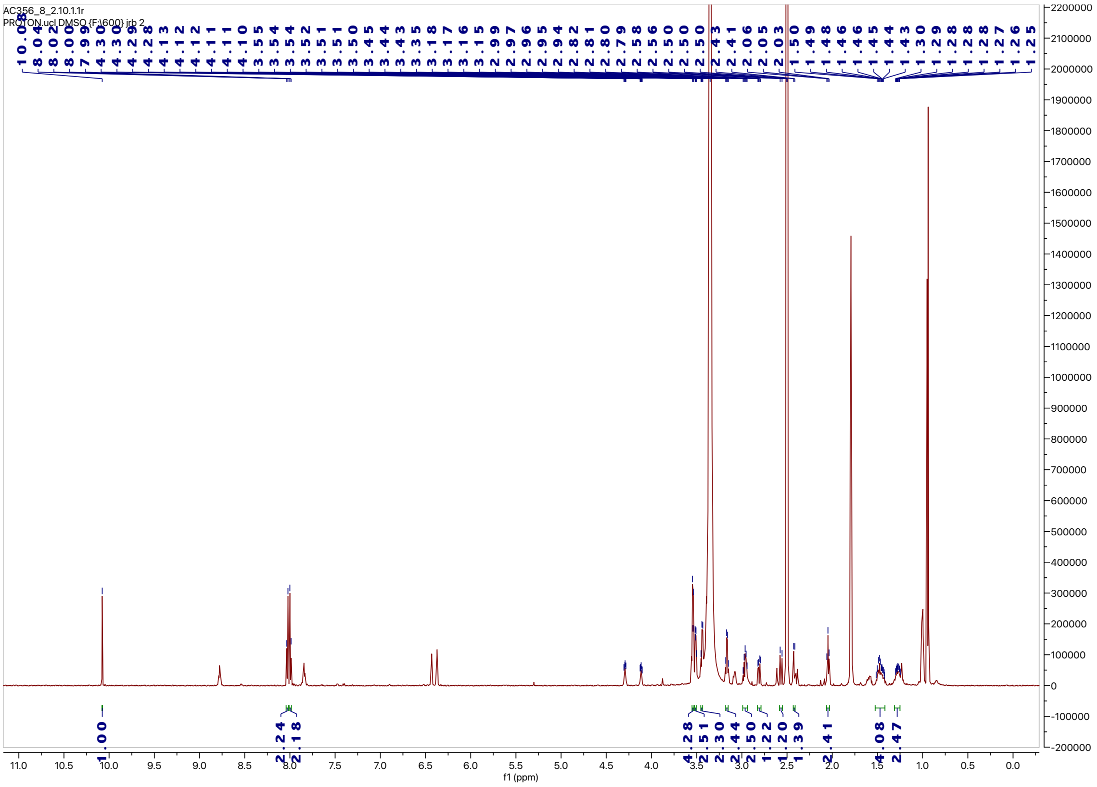
AC356_8_2.10.1.1r
PROTON.ucl DMSO {F:\600} jrb 2
f1 (ppm)

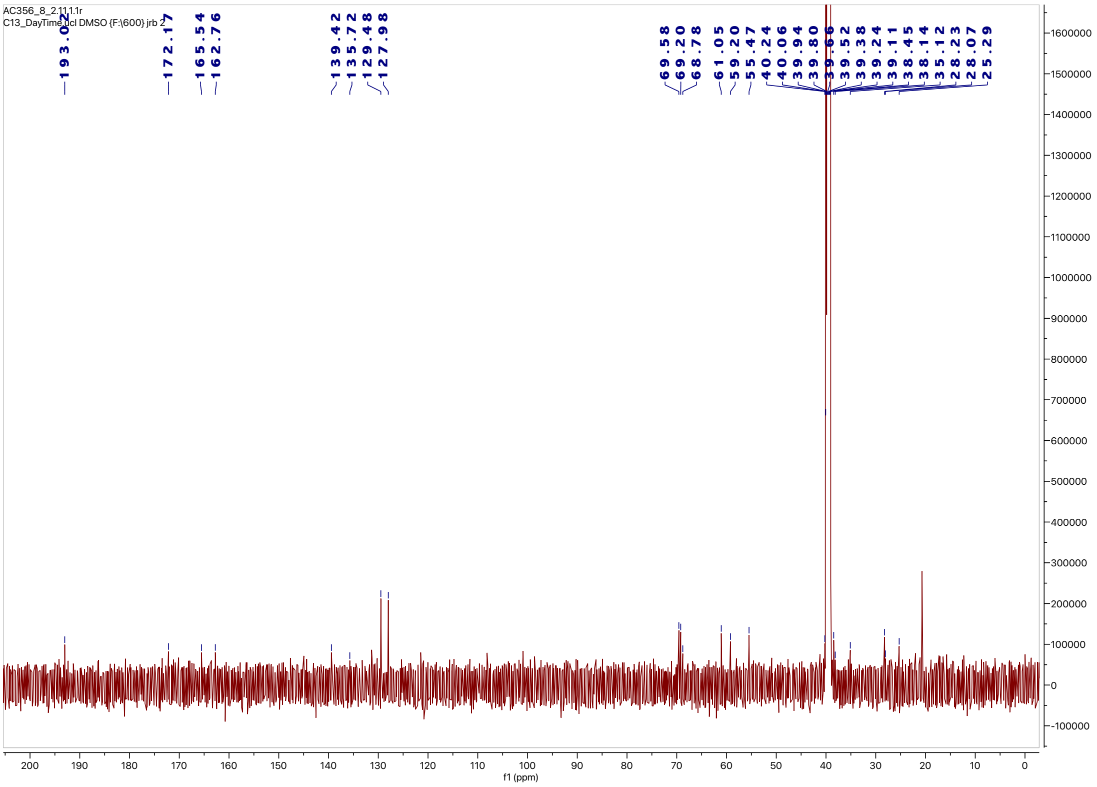
AC356_8_2.11.1.1r
C13_DayTime.ucl DMSO {F:\600} jrb 2
f1 (ppm)

((1*R*,8*S*,9*s*)-bicyclo[6.1.0]non-4-yn-9-yl)methyl (2-(2-(2-(3-(4,5-dibromo-2-methyl-3,6-dioxo-3,6-dihydropyridazin-1(2*H*)-yl)propanamido)ethoxy)ethoxy)ethyl)carbamate[3] (S12)

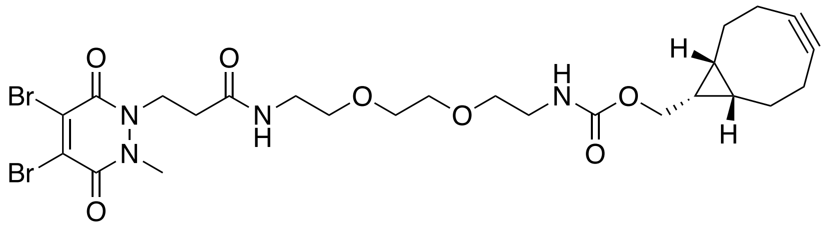

Full characterisation was observed as per Bahou *et al.,* 2021[3].

Bioconjugation general marks

All conjugations experiments were performed in a standard polypropylene Eppendorf safe-lock tubes (1.5 or 2.0 mL) at atmospheric pressure with mixing at the temperature stated. All reagents and solvents were purchased from commercial sources; Sigma UK, Fisher UK or VWR UK and used as per manufacturer instructions. Buffers were prepared with double-deionised water and filter sterilised (0.20 µm). Borate Buffer Saline (BBS) contains 50 mM sodium borate, 50 mM sodium chloride, and 5 mM ethylenediaminetetraacetic acid (EDTA) at pH 8.5 or 7.4. Phosphate Buffer Saline (PBS) contains 10 mM phosphate, 138 mM NaCl, 2.7 mM KCl, pH 7.4. Conjugation Buffer contains 40 mM phosphate, 20 mM NaCl, 6 mM EDTA, pH 7.4. Ultrapure DMF was purchased from Sigma and stored under dry conditions. Ultrafiltration was carried out using Amicon Ultra-4 Centrifugal Filter Units with molecular weight cut-off (MWCO) of 10 kDa or in Vivaspin 500 centrifugal concentrators (Sartorius, UK) with molecular weight cut-off (MWCO) of 10 kDa. Centrifugation was performed using Eppendorf 5415R fixed angle bench rotor operating at 14000 rcf at 20 °C or in an Eppendorf 5810 swing-bucket rotor centrifuge operating at 3220 rcf at 20 °C. Trastuzumab (Ontruzant™) was purchased from UCLH in its clinical formulation (Samsung Bioepis, lyophilised). Ontruzant Fab was prepared by a sequential enzymatic digest of the full antibody with pepsin and papain, following a literature procedure.[4] UV-Vis spectroscopy was used to determine protein concentration using NanoDrop One/One Microvolume UV-Vis Spectrophotometer (Thermo Fisher) operating at room temperature. Sample buffer was used as blank for baseline correction with extinction coefficients: ε_{280} = 68590 M^{-1} cm^{-1} for Fab,[5] ε_{505} = 74000 M^{-1} cm^{-1} for Azide-fluor 488, ε_{335} = 9100 M^{-1} cm^{-1} for pyridazinedione (PD) scaffolds. A correction factor at 280 nm of 0.25 for PD, 0.11 for Azide-fluor 488 was employed. Antibody conjugate concentration was determined using the same extinction coefficient as for native trastuzumab Fab (PD were found to have negligible absorbance at 280 nm).

Protein conjugation reactions were monitored by 12% glycine-SDS-PAGE with 6% stacking gel under non-reducing conditions. Samples were mixed 1:1 with SDS non-reducing loading buffer (composition for 5X SDS: 10 g (8 mL) of glycerol, 4 mL dH_2O, 1.6 mL of 10 % (w/v) SDS, 1 mL of 0.5 M Tris-HCl pH 6.8, 0.025 g Coomassie Brilliant Blue R-250) and heated at 75 °C for 5 min before loaded to the gel. Page Ruler Plus Pre-Stained Protein Ladder (Thermo Scientific) was used. Samples were run at 200 V, 60 min at 1 X running buffer. The 10 X running buffer contained 30 g of Tris base, 144 g of glycine and 10 g of SDS in 1 L of dH_2O, with final pH of 8.3. Gels were stained in Coomassie Blue Stain (10% Ammonium Sulfate (100 g), 0.1% Coomassie Brilliant Blue R-250 (500 mg), 3% Phosphoric Acid (30 mL), ethanol (200 mL), and water (1 L). Gels were de-stained with water. It should be noted that for all thioester containing bridged conjugates the SDS-PAGE lane is streaky, and whilst predominantly showing up as a re-bridged Fab, there is also some detectable heavy and light chain species. This is consistent with having a hydrolytically unstable bridge treated at high temperatures under denaturing conditions, as some hydrolysis will be occurring during the analysis breaking the covalent linkage between the chains. The SDS-PAGE gels (Figure S13, S16, S30, S32), have had lanes cut out of the gel that included data not related to the specific experiments. TAT Peptide (CGISYGRKKRRQRRR) was synthesised by Generon, UK. Peptide arrived in >98% purity and was presented in 9.6 mg.

LCMS general remarks

The molecular masses of the conjugated, native antibodies and antibodies fragment was measured using Agilent 6510 QTOF LC-MS system (Agilent, UK). Agilent 1200 HPLC system was built in with Agilent PLRP-S, 1000 Å, 8 μm, 150 mm x 2.1 mm column that was maintained at 60 °C. Protein sample (10 μl, ~ 5 μM) was separated on the column consisting of mobile phase A (water-0.1 % formic acid) and B (acetonitrile - 0.1 % formic acid) using a gradient solution. The flow rate was adjusted to 300 μL/min.

Table 1. LCMS mobile phase A/B gradient elution

Time (min)	Solvent A (%)	Solvent B (%)
0	85	15
2	85	15
3	68	32
4	68	32
14	65	35
18	5	95
20	5	95
22	85	15
25	85	15

Agilent 6510 QTOF mass spectrometer was operated in a positive polarity mode, coupled with an ESI ion source. The ion source parameters were set up with a VCap of 3500 V, a gas temperature at 350 °C, a dry gas flow rate at 10 L/min and a nebulizer of 30 psig. MS Tof was acquired under conditions of a fragmentor at 350 V, a skimmer at 65 V and an acquisition rate at 0.5 spectra/s in a profile mode, within a scan range between 700 and 5000 m/z. The .d data was then analysed by deconvoluting a spectrum to a zero charge mass spectra using a maximum entropy deconvolution algorithm within the MassHunter software version B.07.00.

Native Fab

Fab (30 µL, 100 µM, 4.76 mg/mL) in conjugation buffer was desalted (7 kDa MWCO, ZebaSpin) prior to LCMS analysis. Concentration was determined photometrically using ε_{280} = 68590 M^{-1} cm^{-1}.

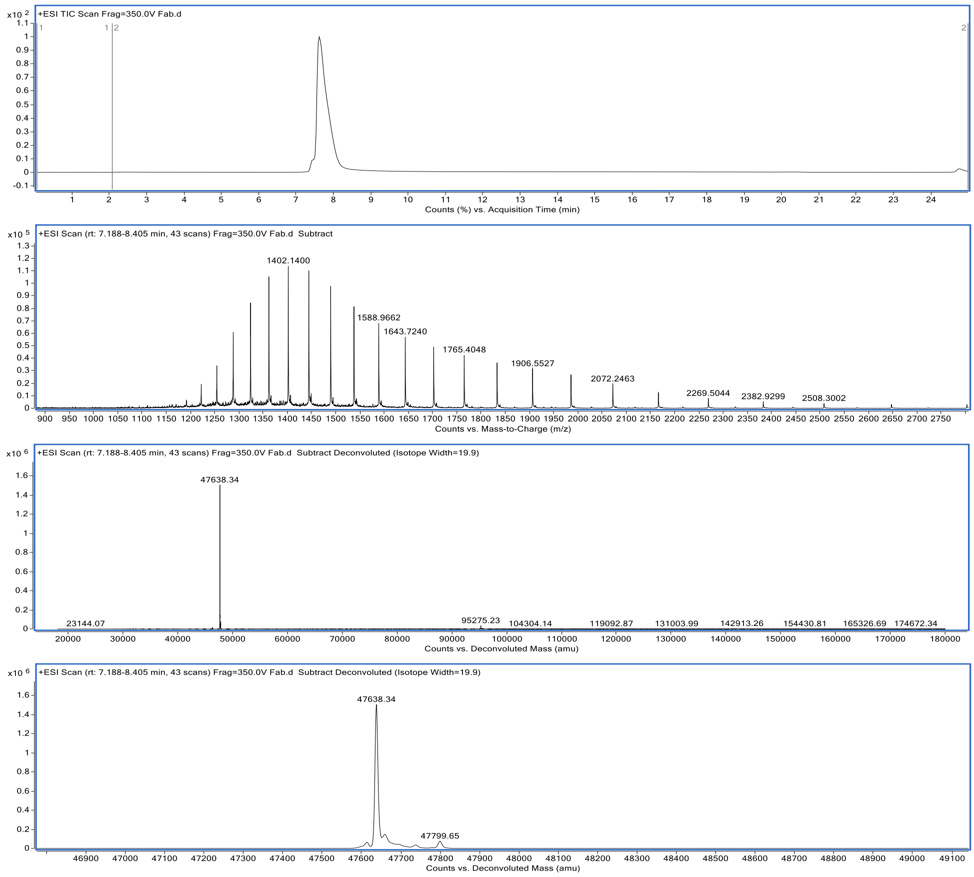

a)
b)
c)
d)

Figure S1. LCMS analysis of native Fab; a) TIC, b) non-deconvoluted ion-series, c) full range deconvoluted ion series mass spectrum, d) zoomed in deconvoluted ion series mass spectrum; native Fab expected 47638, observed 47638.

Reduced Fab

Fab (30 µL, 100 µM, 4.76 mg/mL) in conjugation buffer was reduced with tris(2-carboxyethyl)phosphine (TCEP) (2 µL, 15 mM in diH_2O, 10 eq.) The mixture was incubated at 37 °C for 1.5 h, 300 rpm. Lastly, sample was desalted (7 kDa MWCO, ZebaSpin) prior to LCMS analysis. Concentration was determined photometrically using ε_{280} = 68590 M^{-1} cm^{-1}.

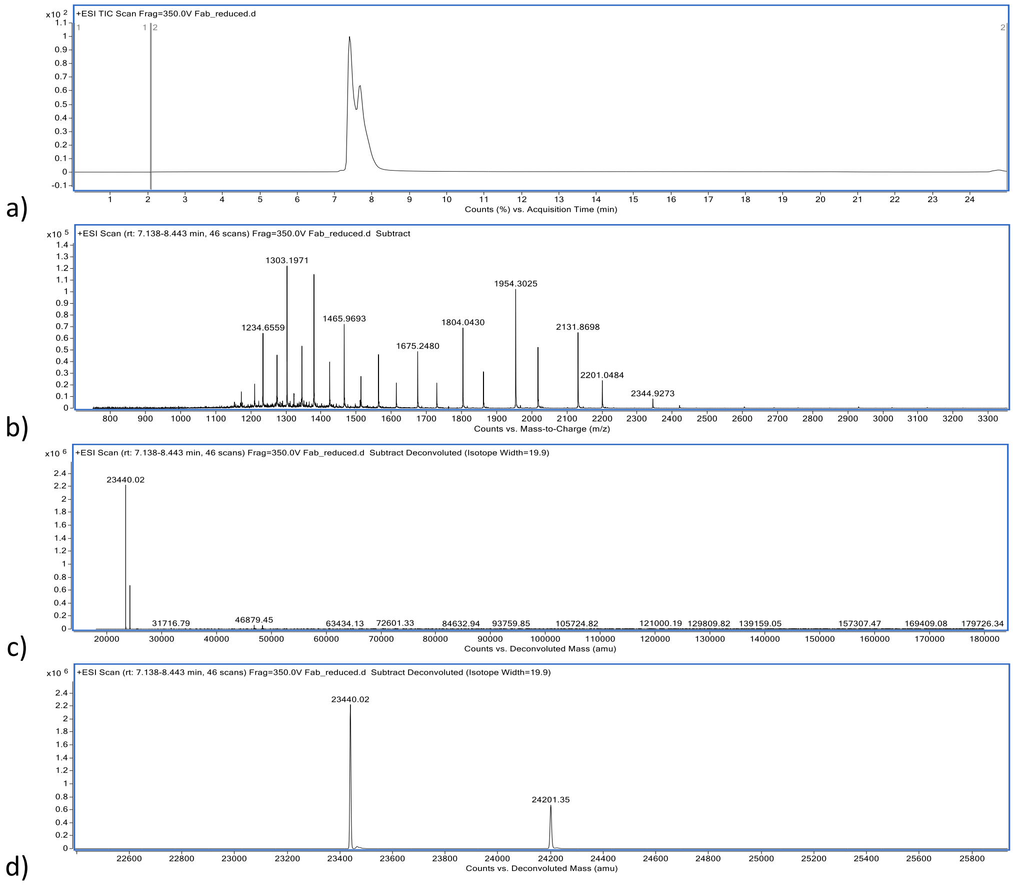

Figure S2. LCMS analysis of reduced Fab; a) TIC, b) non-deconvoluted ion-series, c) full range deconvoluted ion series mass spectrum, d) zoomed in deconvoluted ion series mass spectrum; native light chain expected 23438, observed 23440; native heavy chain expected 24200, observed 24201.

Reaction of Fab with α-chlorothioester (1)

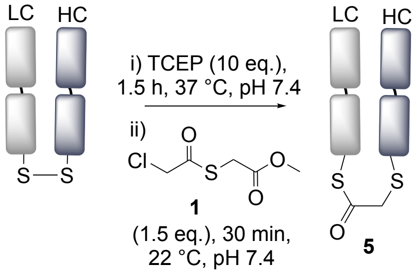

Fab (30 µL, 100 µM, 4.76 mg/mL) in conjugation buffer was reduced with tris(2-carboxyethyl)phosphine (TCEP) (2.0 µL, 15 mM in diH_2O, 10 eq.) The mixture was incubated at 37 °C for 1.5 h, 300 rpm. Following that, α-chlorothioester **1** (0.4 µl, 12 mM in DMF, 1.5 eq.) was added and incubated at 22 °C for 30 min. Lastly, sample was desalted into HPLC grade water (7 kDa MWCO, ZebaSpin) prior to LCMS analysis. Concentration was determined photometrically using ε_{280} = 68590 M^{-1} cm^{-1}.

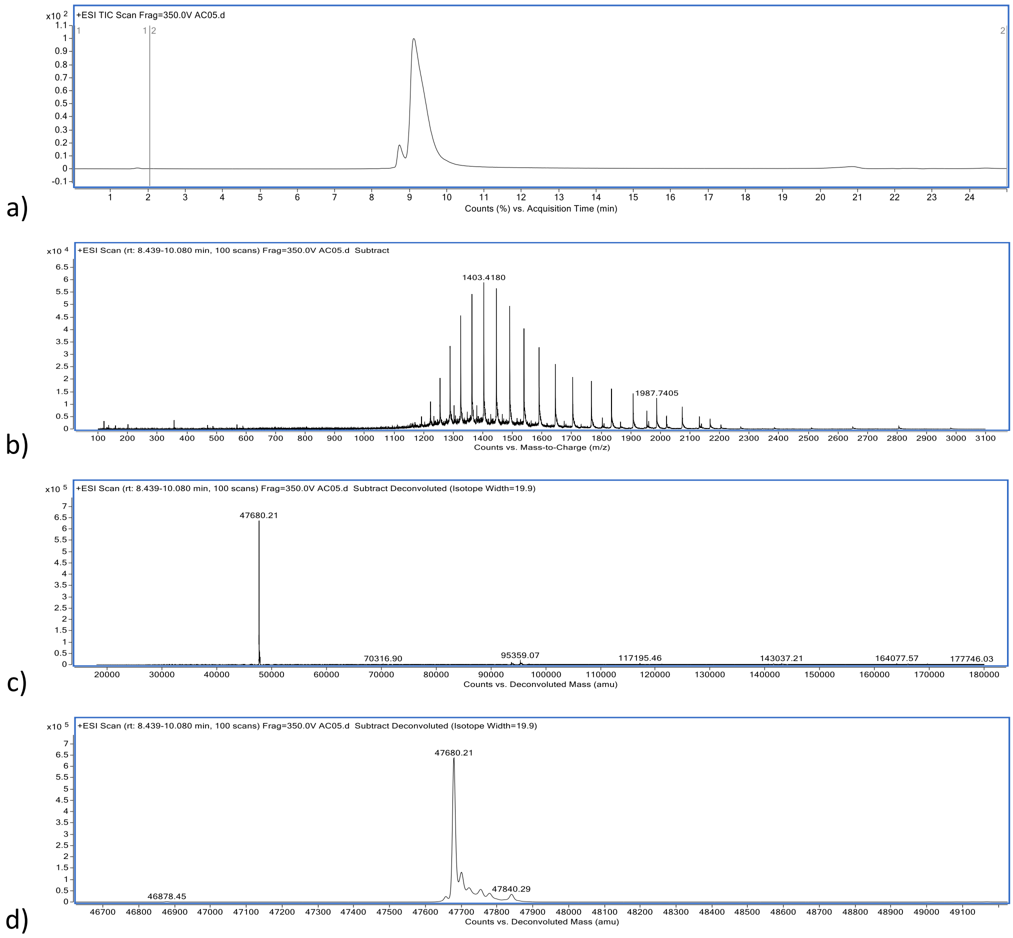

Figure S3. LCMS analysis of rebridging Fab with α-chlorothioester **1**; a) TIC, b) non-deconvoluted ion-series, c) full range deconvoluted ion series mass spectrum, d) zoomed in deconvoluted ion series mass spectrum; rebridged Fab with α-chlorothioester **1** expected 47680, observed 47680.

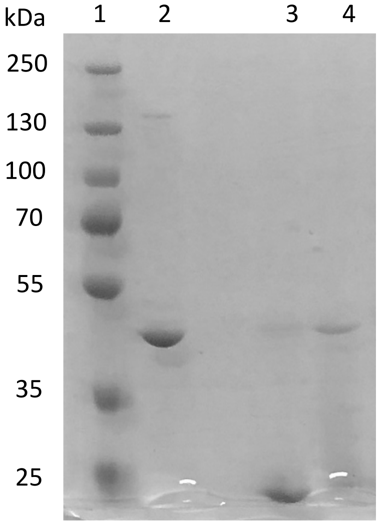

Figure S4. SDS-PAGE of rebridging Fab with α-chlorothioester **1**; 1 – molecular marker, 2 – Native Fab, 3 – Reduced Fab, 4 – rebridged Fab with **1**.

Reaction of Fab conjugate 5 and cysteine

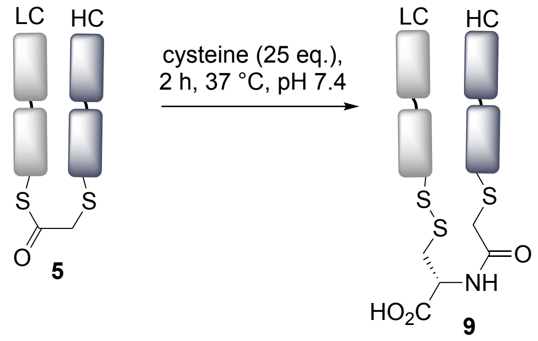

Fab (30 µL, 100 µM, 4.76 mg/mL) in conjugation buffer was reduced with tris(2-carboxyethyl)phosphine (TCEP) (2.0 µL, 15 mM in diH_2O, 10 eq.). The mixture was incubated at 37 °C for 1.5 h, 300 rpm. Following that, α-chlorothioester **1** (0.4 µl, 12 mM in DMF, 1.5 eq.) was added and incubated at 22 °C for 30 min. After that, L-cysteine (0.7 µL, 108 mM in diH_2O, 25 eq.) was added and left for at 37 °C, for 2 h, 300 rpm. Lastly, sample was desalted into HPLC grade water (7 kDa MWCO, ZebaSpin) prior to LCMS analysis. Concentration was determined photometrically using ε_{280} = 68590 M^{-1} cm^{-1}.

a)

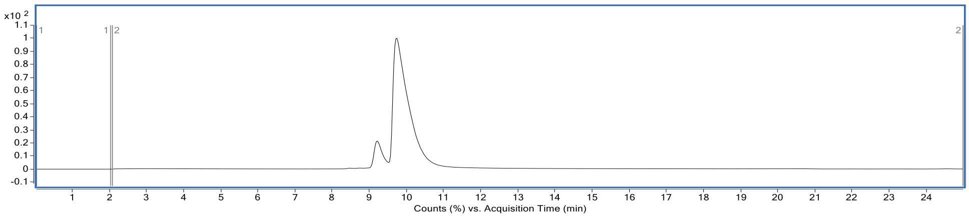

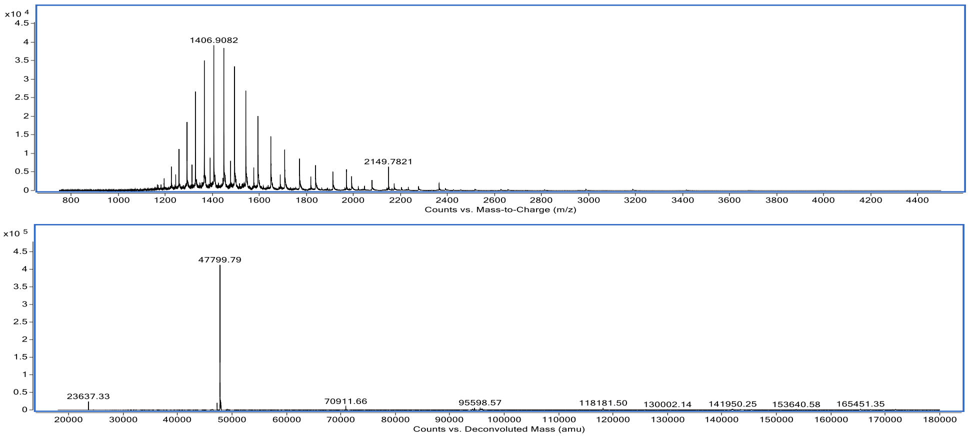

b)

c)

Figure S5. LCMS analysis of cysteine conjugate **9**; a) TIC, b) non-deconvoluted ion-series, c) full range deconvoluted ion series mass spectrum; Fab conjugate with cysteine expected 47799, observed 47799.

Reaction of Fab conjugate 5 with cysteine and *N*-Me-maleimide

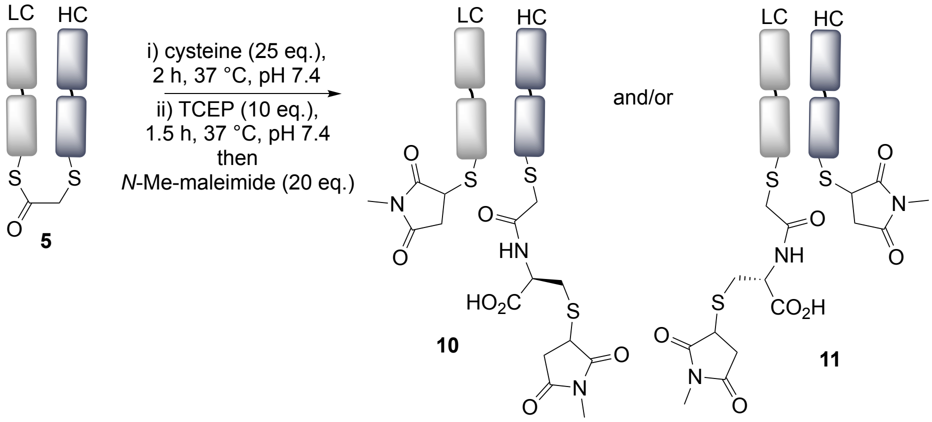

Fab (20 µL, 80 µM, 3.81 mg/mL) in conjugation buffer was reduced with tris(2-carboxyethyl)phosphine (TCEP) (1.1 µL, 15 mM in diH_2O, 10 eq.) The mixture was incubated at 37 °C for 1.5 h, 300 rpm. Following that, α-chlorothioester **1** (0.2 µl, 12 mM in DMF, 1.5 eq.) was added and incubated at 22 °C for 30 min. After that, L-cysteine (0.4 µL, 108 mM in diH_2O, 25 eq.) was added and left for at 37 °C, for 2 h, 300 rpm. After this period, the excess reagent was removed *via* ultrafiltration (10 kDa MWCO), into conjugation buffer, new Fab concentration was determined, and TCEP reduction was performed again as before. To cap the free thiols, an excess of *N*-methylmaleimide was added (39 mM in diH_2O, 20 eq.). Lastly, sample was desalted (7 kDa MWCO, ZebaSpin) prior to LCMS analysis. Concentration was determined photometrically using ε_{280} = 68590 M^{-1} cm^{-1}.

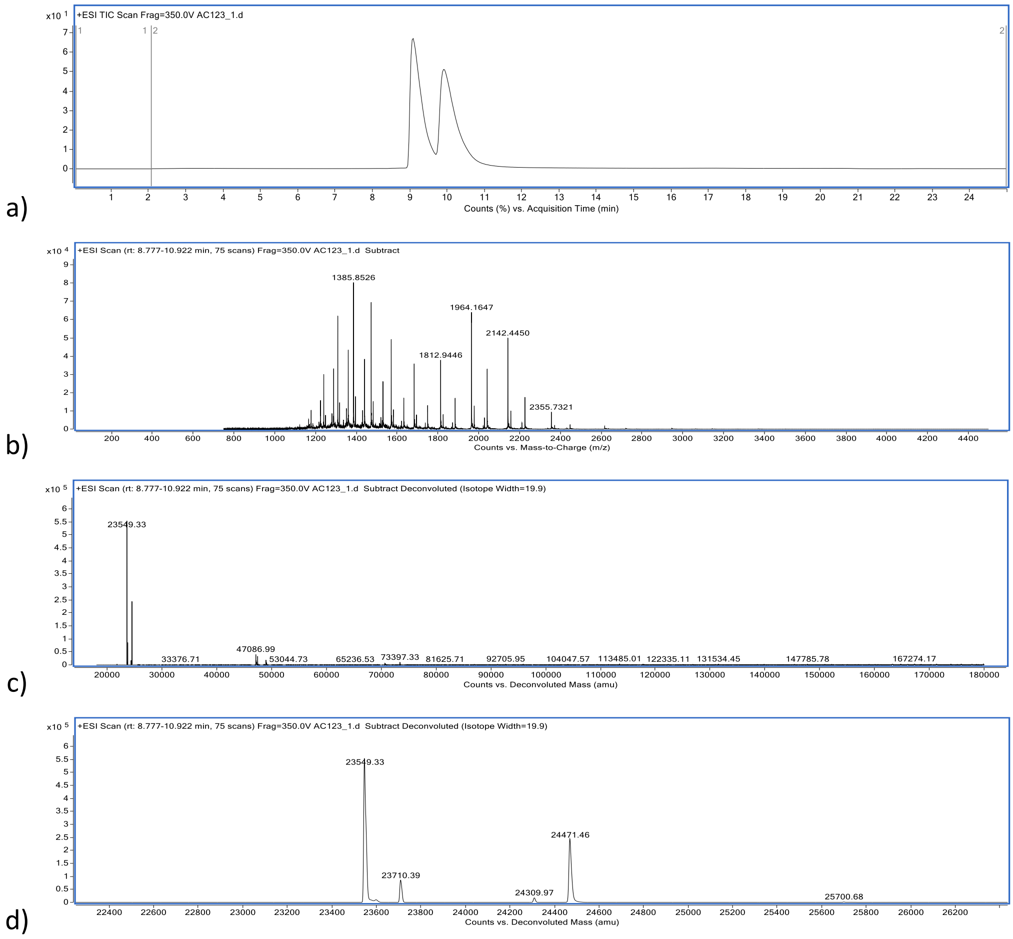

Figure S6. LCMS analysis of regioselectivity preference of α-chlorothioester **1**; a) TIC, b) non-deconvoluted ion-series, c) full range deconvoluted ion series mass spectrum, d) zoomed in deconvoluted ion series mass spectrum; **10** (LC) expected 23550, observed 23549; **11** (LC) expected 23711, observed 23710; **11** (HC) expected 24312, observed 24309; **10** (HC) expected 24473, observed 24471.

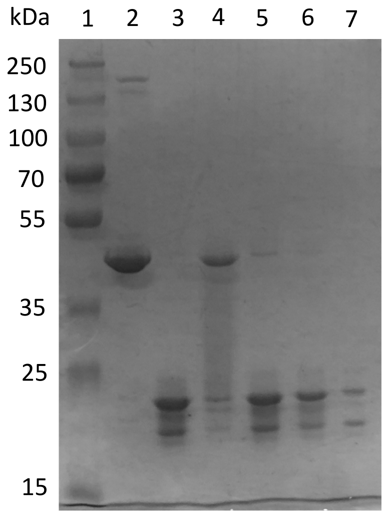

Figure S7. SDS-PAGE of regioselectivity preference of α-chlorothioester **1**; 1 – molecular marker, 2 – Native Fab, 3 – Reduced Fab, 4 – rebridged Fab with **1**, 5 – treatment of rebridged Fab with cysteine, 6 – 2nd TCEP reduction step, 7 – *N*-Me-maleimide capped.

Stability study of Fab conjugate 5

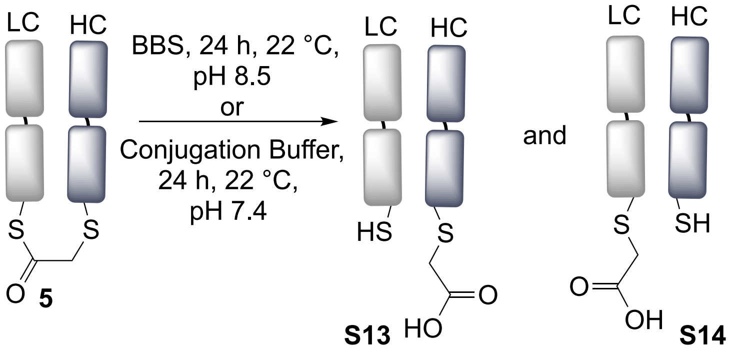

Fab (30 µL, 100 µM, 4.76 mg/mL) in conjugation buffer was reduced with tris(2-carboxyethyl)phosphine (TCEP) (2.0 µL, 15 mM in diH_2O, 10 eq.) The mixture was incubated at 37 °C for 1.5 h, 300 rpm. Following that, α-chlorothioester **1** (0.4 µl, 12 mM in DMF, 1.5 eq.) was added and incubated at 22 °C for 30 min. After that, the buffer was swapped into BBS pH 8.5 or pH 7.4 (3 x ultrafiltration) and left for 24 h at 22 °C. Lastly, sample was desalted into HPLC grade water (7 kDa MWCO, ZebaSpin) prior to LCMS analysis. Concentration was determined photometrically using ε_{280} = 68590 M^{-1} cm^{-1}.

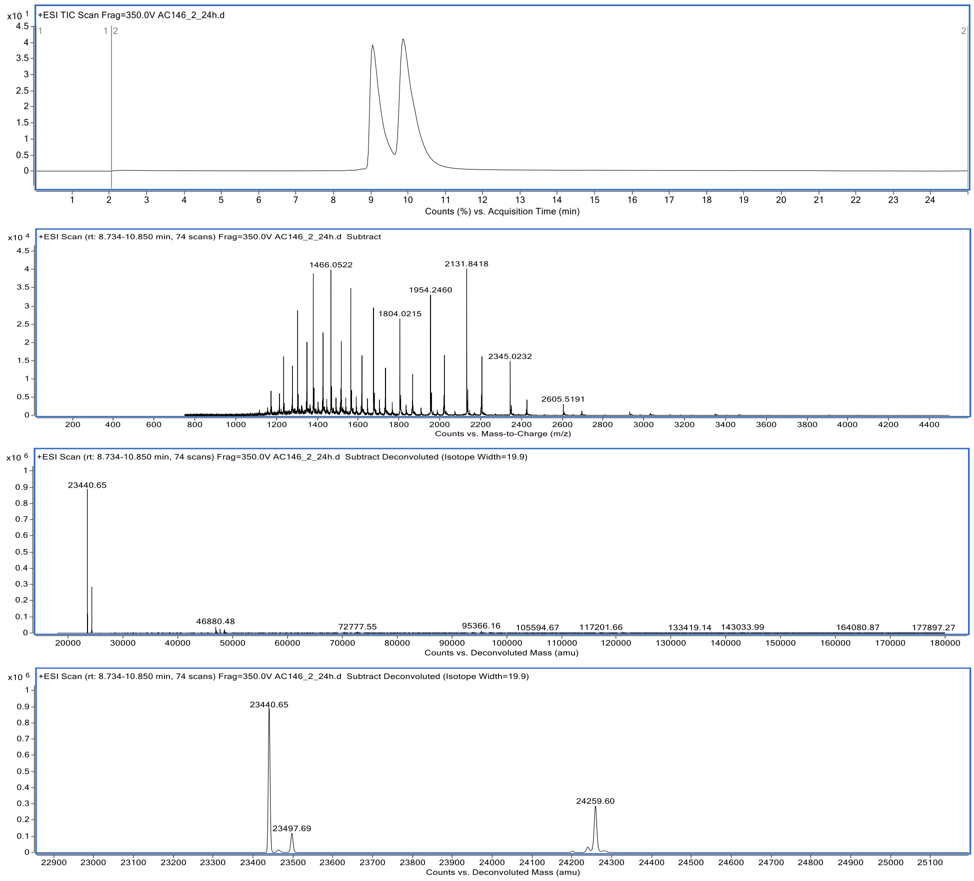

a)
b)
c)
d)

Figure S8. Stability study of Fab conjugate **5** at pH 8.5, 24 h. a) TIC, b) non-deconvoluted ion-series c) full range deconvoluted ion series mass spectrum, d) zoomed in deconvoluted ion-series of LC and HC

at 24 h. **S13** (LC) expected 23438, observed 23440, **S14** (LC) expected 23497, observed 23497, **S14** (HC) expected 24200, observed 24200, **S13** (HC) expected 24259, observed 24259.

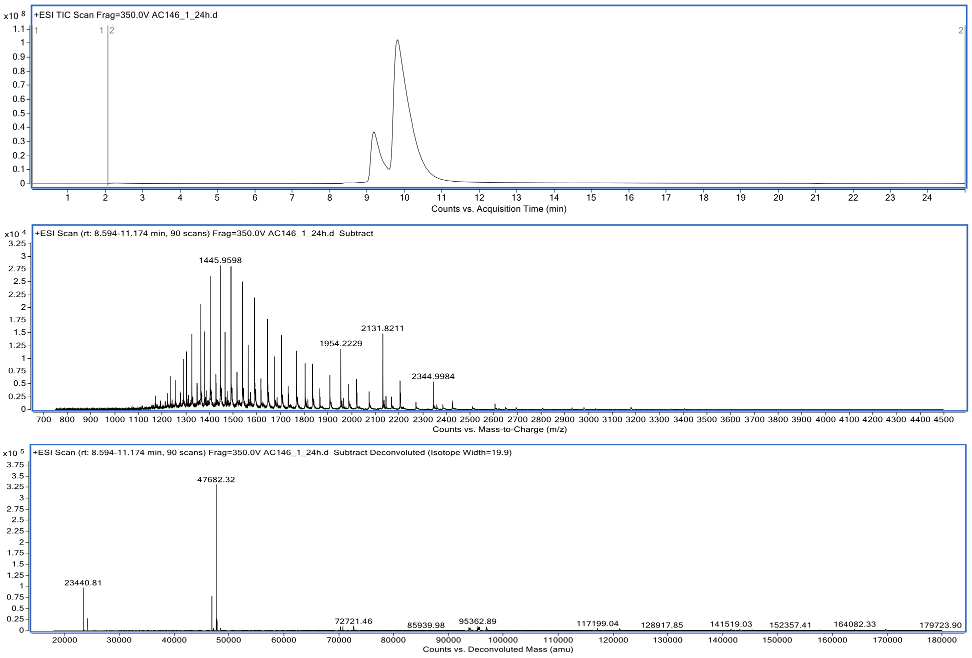

a) b) c)

Figure S9. Stability study of Fab conjugate **5** at pH 7.4, 24 h. a) TIC, b) non-deconvoluted ion-series c) full range deconvoluted ion series mass spectrum, rebridged Fab with α-chlorothioester **1** expected 47680, observed 47682.

Reaction of Fab with methyl 2-(acryloylthio)acetate (2)

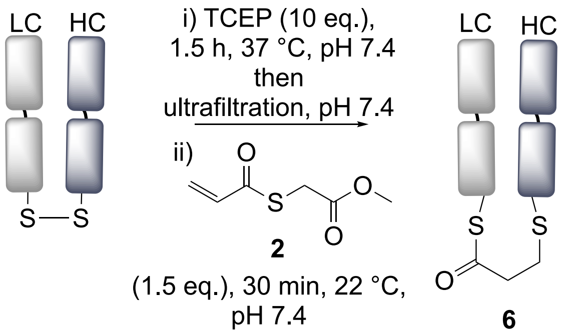

Fab (30 µL, 100 µM, 4.76 mg/mL) in conjugation buffer was reduced with tris(2-carboxyethyl)phosphine (TCEP) (2.0 µL, 15 mM in diH_2O, 10 eq.). The mixture was incubated at 37 °C for 1.5 h, 300 rpm. The excess of TCEP was removed *via* ultrafiltration (10 kDa MWCO) into conjugation buffer and new Fab concentration was determined. Following that, methyl 2-(acryloylthio)acetate **2** (51 mM in DMF, 1.5 eq.) was added and incubated at 22 °C for 30 min. Lastly, sample was desalted into HPLC grade water (7 kDa MWCO, ZebaSpin) prior to LCMS analysis. Concentration was determined photometrically using ε_{280} = 68590 M^{-1} cm^{-1}.

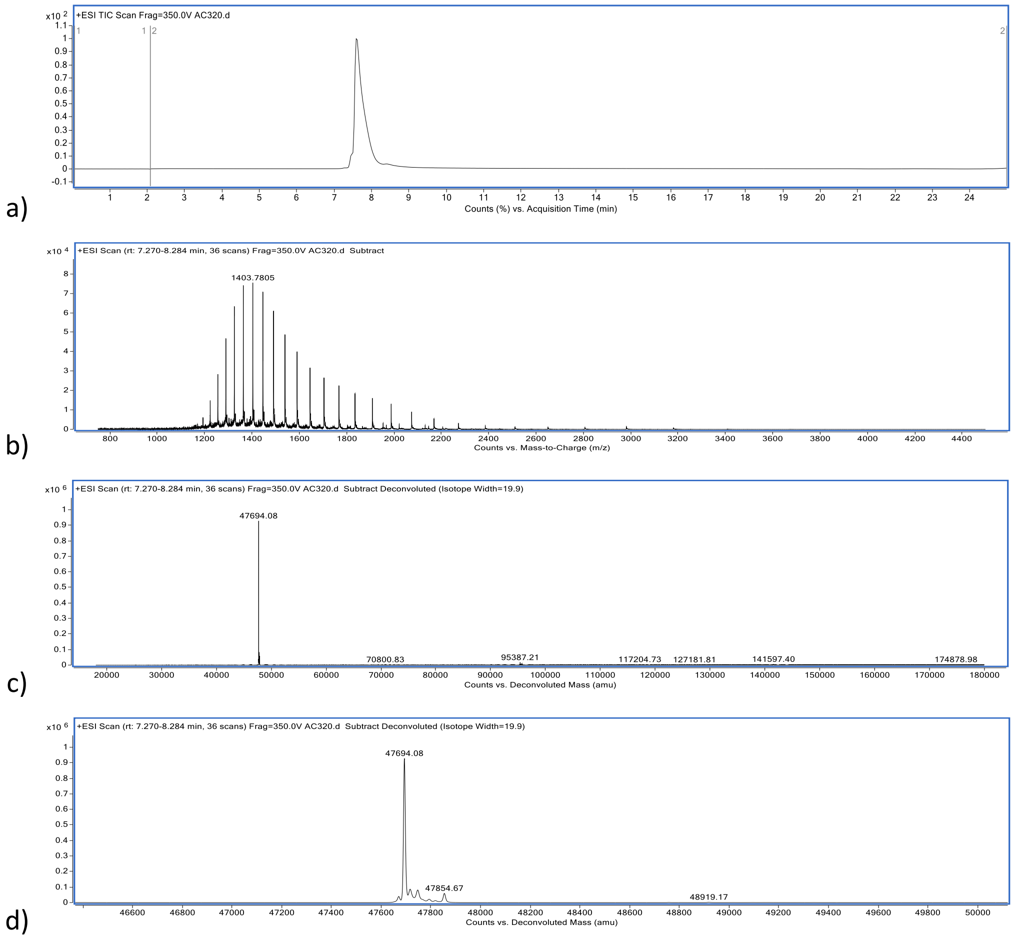

Figure S10. LCMS analysis of rebridging Fab with methyl 2-(acryloylthio)acetate **2**; a) TIC, b) non-deconvoluted ion-series, c) full range deconvoluted ion series mass spectrum, d) zoomed in deconvoluted ion series mass spectrum; rebridged Fab with methyl 2-(acryloylthio)acetate **2** expected 47694, observed 47694.

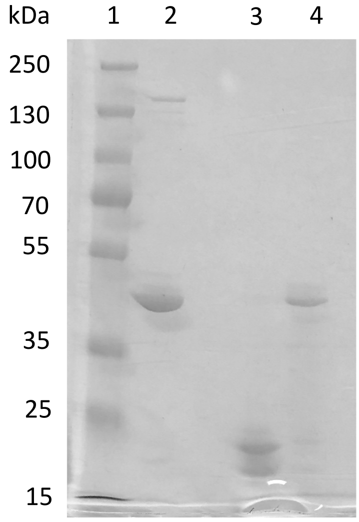

Figure S11. SDS-PAGE of rebridging Fab with methyl 2-(acryloylthio)acetate **2**; 1 – molecular marker, 2 – Native Fab, 3 – Reduced Fab, 4 – rebridged Fab with **2**.

Reaction of Fab conjugate 6 with cysteine and *N*-Me-maleimide

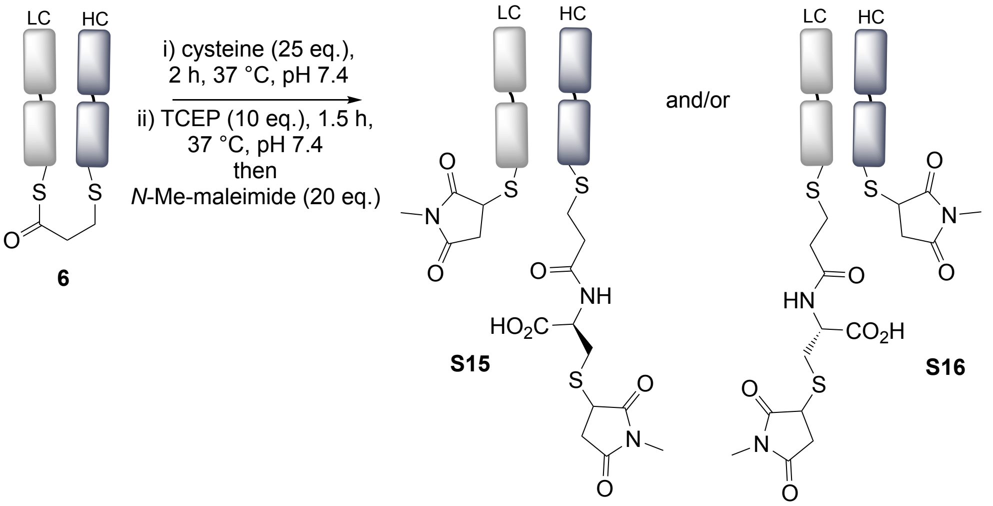

Fab (30 µL, 100 µM, 4.76 mg/mL) in conjugation buffer was reduced with tris(2-carboxyethyl)phosphine (TCEP) (2.0 µL, 15 mM in diH_2O, 10 eq.). The mixture was incubated at 37 °C for 1.5 h, 300 rpm. The excess of TCEP was removed *via* ultrafiltration (10 kDa MWCO) into conjugation buffer, new Fab concentration was determined. Following that, methyl 2-(acryloylthio)acetate **2** (11 mM in DMF, 1.5 eq.) was added and incubated at 22 °C for 30 min. After that, L-cysteine (108 mM in diH_2O, 25 eq.) was added and left for at 37 °C, for 2 h, 300 rpm. After this period, the excess reagent was removed *via* ultrafiltration (10 kDa MWCO), into conjugation buffer and TCEP reduction was performed again as before. To cap the free thiols, an excess of *N*-methylmaleimide was added (39 mM in diH_2O, 20 eq.). Lastly, sample was desalted (7 kDa MWCO, ZebaSpin) prior to LCMS analysis. Concentration was determined photometrically using ε_{280} = 68590 M^{-1} cm^{-1}.

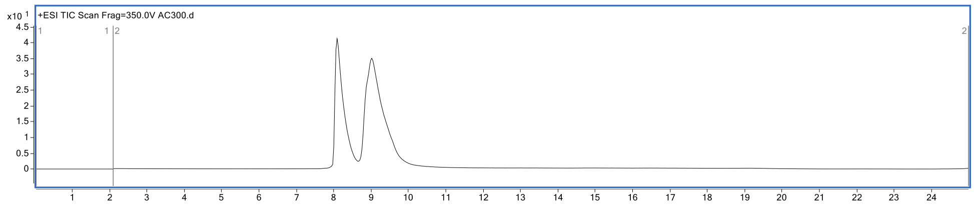

a)

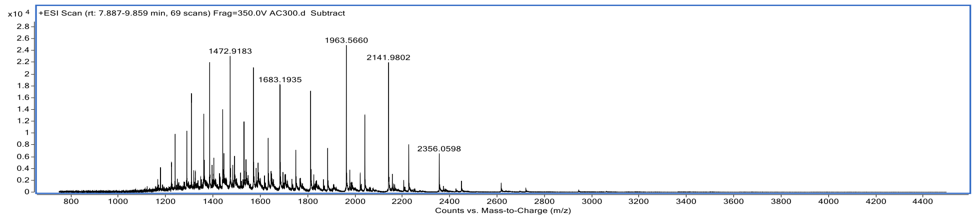

b)

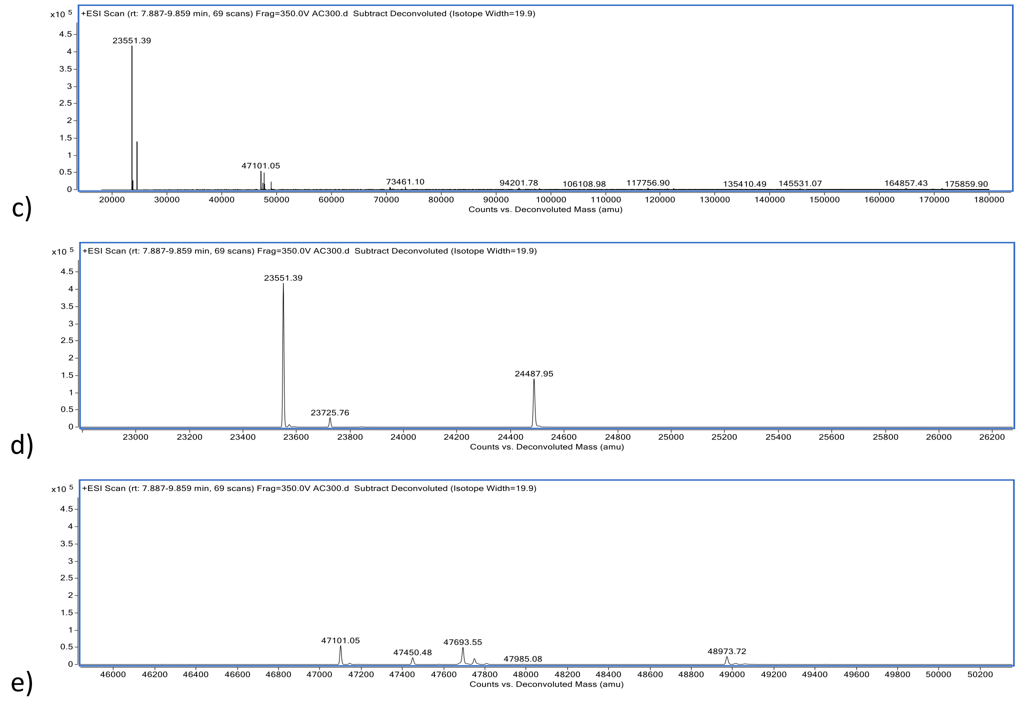

Figure S12. LCMS analysis of regioselectivity preference of methyl 2-(acryloylthio)acetate **2**; a) TIC, b) non-deconvoluted ion-series, c) full range deconvoluted ion series mass spectrum, d) zoomed in deconvoluted ion series mass spectrum; **S15** (LC) expected 23550, observed 23551, **S16** (LC) expected 23725, observed 23725, **S16** (HC) expected 24312, not observed, **S15** (HC) expected 24487, observed 24487, e) zoomed in deconvoluted ion series mass spectrum; rebridged Fab with methyl 2-(acryloylthio)acetate **2** expected 47694, observed 47693. Peaks 47101 (2 x **S15** (LC)), 47450 (2 x **S16** (LC)), and 48973 (2 x **S15** (HC)) are proposed to be artifacts formed during ionisation.

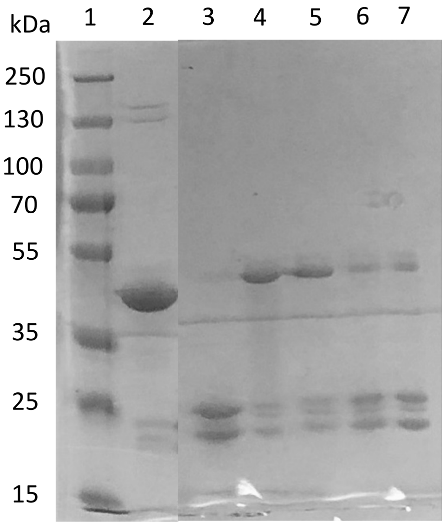

Figure S13. SDS-PAGE of regioselectivity preference of methyl 2-(acryloylthio)acetate **2** and cysteine; 1 – molecular marker, 2 – Native Fab, 3 – Reduced Fab, 4 – rebridged Fab with **2**, 5 – treatment of rebridged Fab with cysteine, 6 – 2nd TCEP reduction step, 7 – *N*-Me-maleimide capped.

Stability study of Fab conjugate 6

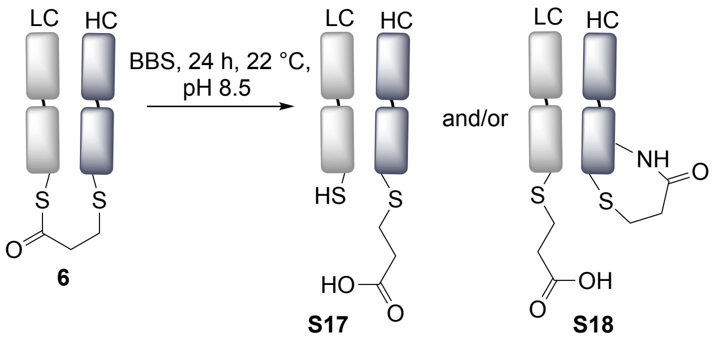

Fab (30 µL, 100 µM, 4.76 mg/mL) in conjugation buffer was reduced with tris(2-carboxyethyl)phosphine (TCEP) (2.0 µL, 15 mM in diH_2O, 10 eq.). The mixture was incubated at 37 °C for 1.5 h, 300 rpm. The excess of TCEP was removed *via* ultrafiltration (10 kDa MWCO) into conjugation buffer, new Fab concentration was determined. Following that, methyl 2-(acryloylthio)acetate **2** (51 mM in DMF, 1.5 eq.) was added and incubated at 22 °C for 30 min. After that, the buffer was swapped into BBS pH 8.5 (3 x ultrafiltration) and left for 24 h at 22 °C. Lastly, sample was desalted into HPLC grade water (7 kDa MWCO, ZebaSpin) prior to LCMS analysis. Concentration was determined photometrically using ε_{280} = 68590 M^{-1} cm^{-1}.

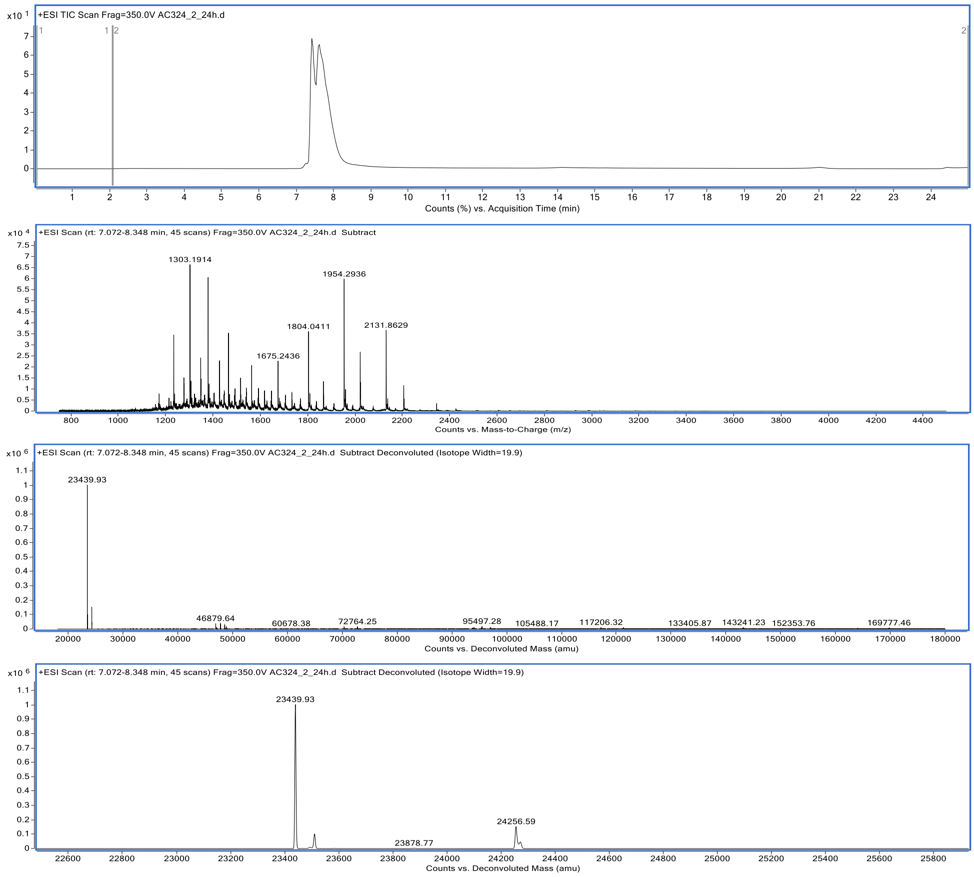

a) b) c) d)

Figure S14. Stability study of Fab conjugate **6** at pH 8.5. a) TIC, b) non-deconvoluted ion-series c) full range deconvoluted ion series mass spectrum, d) zoomed in deconvoluted ion-series of LC and HC at 24 h. **S17** (LC) expected 23438, observed 23439, **S18** (LC) expected 23511, observed 23511, **S18** (HC) expected 24256, observed 24256, **S17** (HC) expected 24273, observed 24273.

Reaction of Fab with methyl 2-((2-chloro-2-phenylacetyl)thio)acetate (3)

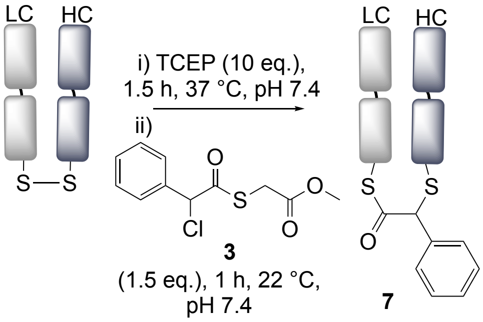

Fab (30 µL, 100 µM, 4.76 mg/mL) in conjugation buffer was reduced with tris(2-carboxyethyl)phosphine (TCEP) (2.0 µL, 15 mM in diH_2O, 10 eq.). The mixture was incubated at 37 °C for 1.5 h, 300 rpm. Following that, methyl 2-((2-chloro-2-phenylacetyl)thio)acetate **3** (0.1 µl, 43 mM in DMF, 1.5 eq.) was added and incubated at 22 °C for 1 h. Lastly, sample was desalted into HPLC grade water (7 kDa MWCO, ZebaSpin) prior to LCMS analysis. Concentration was determined photometrically using ε_{280} = 68590 M^{-1} cm^{-1}.

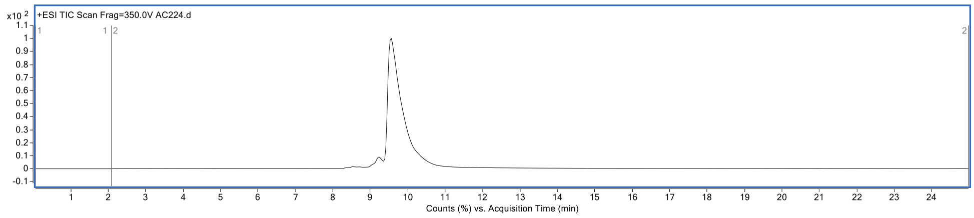

a)

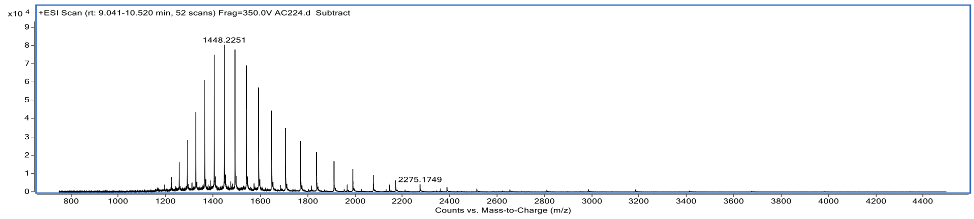

b)

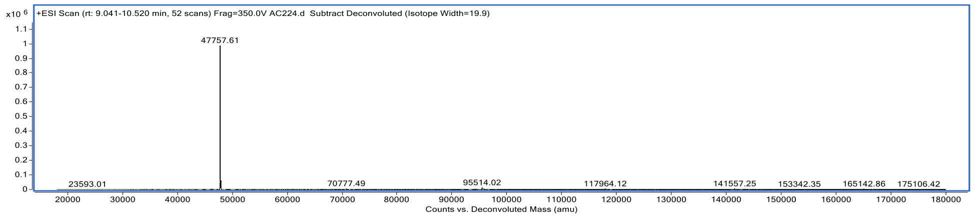

c)

d)
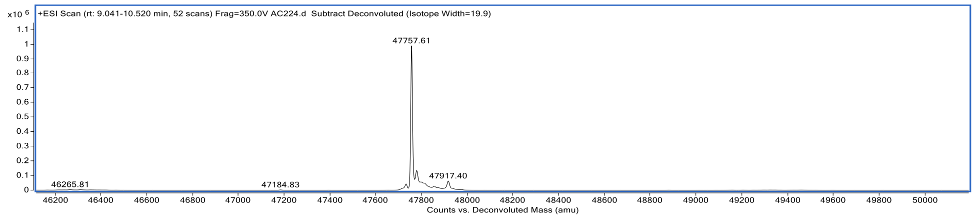

Figure S15. LC-MS analysis of rebridging Fab with 2-((2-chloro-2-phenylacetyl)thio)acetate **3**; a) TIC, b) non-deconvoluted ion-series, c) full range deconvoluted ion series mass spectrum, d) zoomed in deconvoluted ion series mass spectrum; rebridged Fab with 2-((2-chloro-2-phenylacetyl)thio)acetate **3** expected 47756, observed 47757.

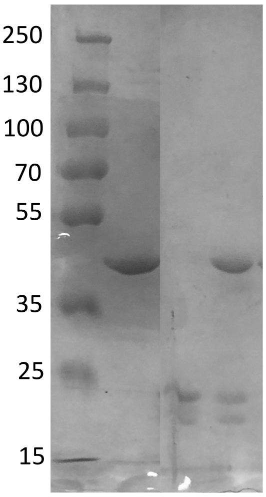

Figure S16. SDS-PAGE of rebridged Fab with 2-((2-chloro-2-phenylacetyl)thio)acetate **3**; 1 – molecular marker, 2 – Native Fab, 3 – Reduced Fab, 4 – rebridged Fab with **3**.

Reaction of Fab conjugate 7 with cysteine and *N*-Me-maleimide

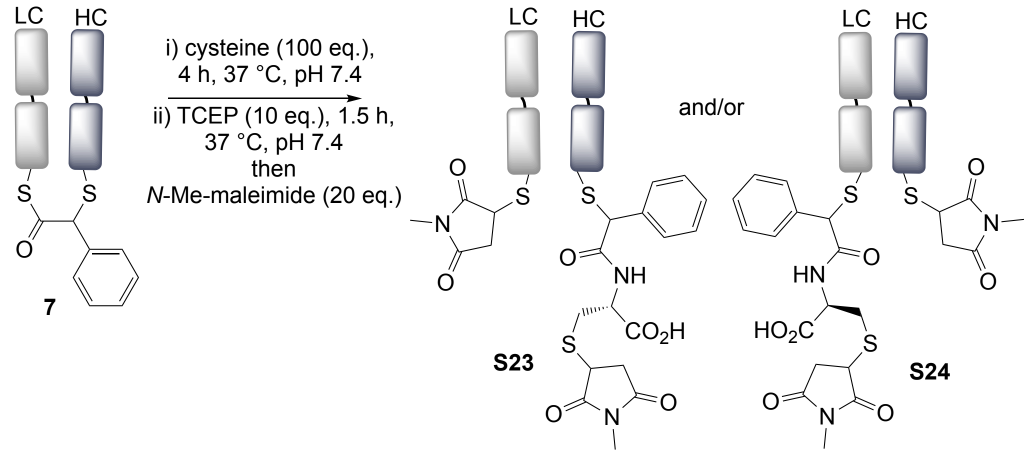

Fab (30 µL, 100 µM, 4.76 mg/mL) in conjugation buffer was reduced with tris(2-carboxyethyl)phosphine (TCEP) (2.0 µL, 15 mM solution in diH_2O, 10 eq.). The mixture was incubated at 37 °C for 1.5 h, 300 rpm.

Following that, 2-((2-chloro-2-phenylacetyl)thio)acetate **3** (0.1 µl, 43 mM in DMF, 1.5 eq.) was added and incubated at 22 °C for 1 h. After that, L-cysteine (2.7 µL, 108 mM in diH_2O, 100 eq.) was added and left for at 37 °C, for 4 h, 300 rpm. After this period, the excess reagent was removed *via* ultrafiltration (10 kDa MWCO), into conjugation buffer, new Fab concentration was determined, and TCEP reduction was performed again as before. To cap the free thiols, an excess of *N*-methylmaleimide was added (39 mM in diH_2O, 20 eq.). Lastly, sample was desalted into HPLC grade water (7 kDa MWCO, ZebaSpin) prior to LCMS analysis. Concentration was determined photometrically using ε_{280} = 68590 M^{-1} cm^{-1}.

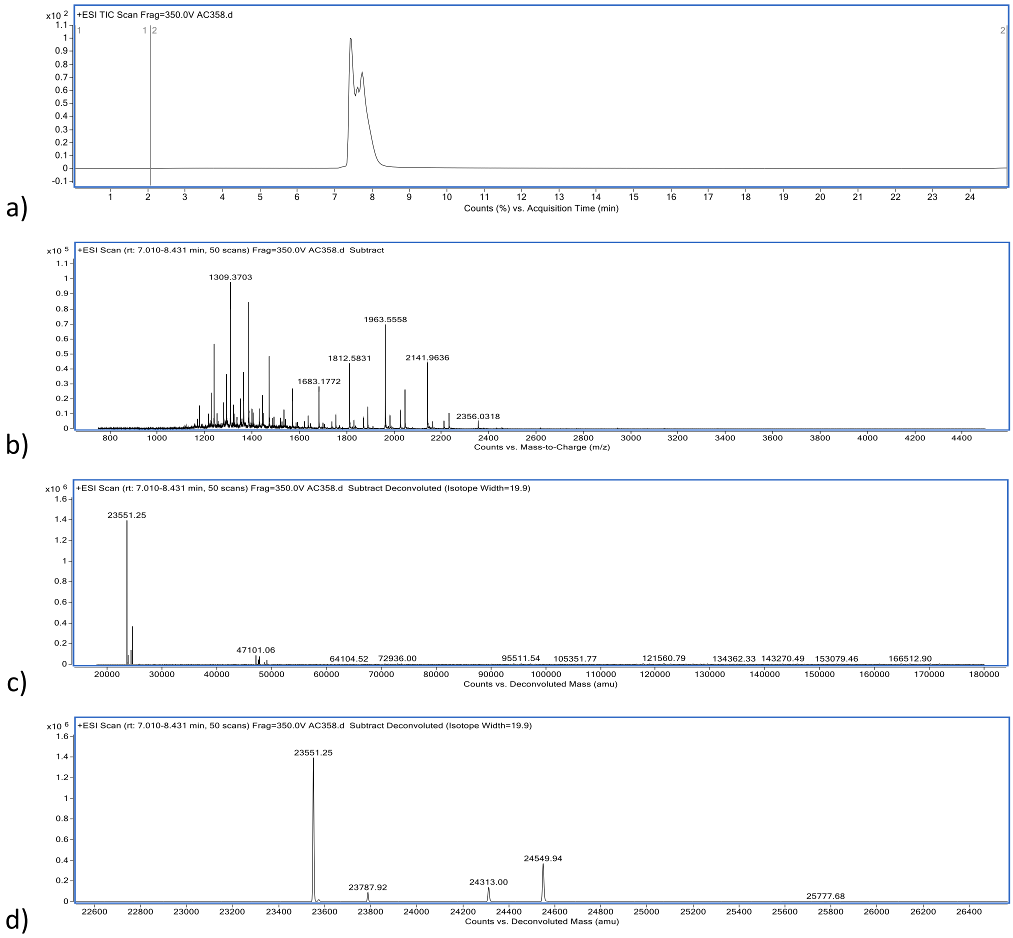

Figure S17. LCMS analysis of regioselectivity preference of 2-((2-chloro-2-phenylacetyl)thio)acetate **3**; a) TIC, b) non-deconvoluted ion-series, c) full range deconvoluted ion series mass spectrum, d) zoomed in deconvoluted ion series mass spectrum; **S23** (LC) expected 23550, observed 23551, **S24** (LC) expected 23787, observed 23787, **S24** (HC) expected 24312, observed 24313, **S23** (HC) expected 24549, observed 24549.

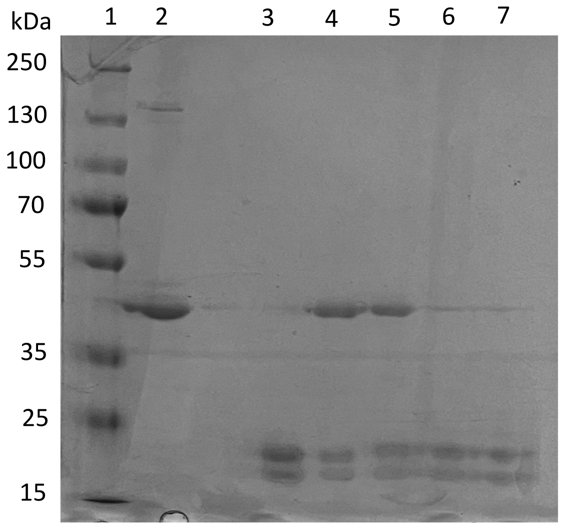

Figure S18. SDS-PAGE analysis of regioselectivity preference of 2-((2-chloro-2-phenylacetyl)thio)acetate **3** and cysteine; 1 – molecular marker, 2 – Native Fab, 3 – Reduced Fab, 4 – rebridged Fab with **3**, 5 – treatment of rebridged Fab with cysteine, 6 – 2nd TCEP reduction, 7 – *N*-Me-maleimide capped.

Stability study of Fab conjugate 7

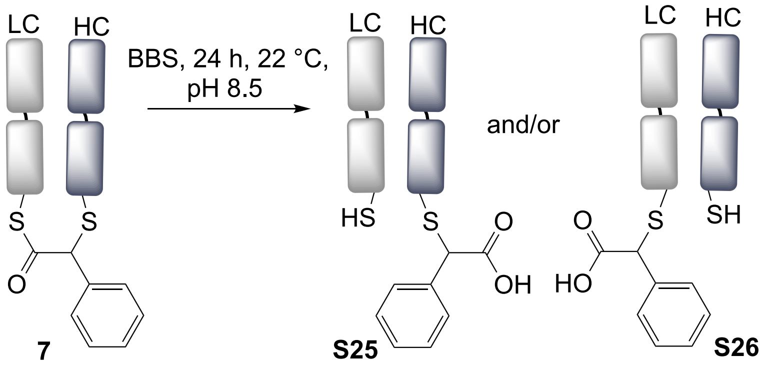

Fab (30 µL, 100 µM, 4.76 mg/mL) in conjugation buffer was reduced with tris(2-carboxyethyl)phosphine (TCEP) (2.0 µL, 15 mM in diH_2O, 10 eq.). The mixture was incubated at 37 °C for 1.5 h, 300 rpm. Following that, methyl 2-((2-chloro-2-phenylacetyl)thio)acetate **3** (0.1 µl, 43 mM in DMF, 1.5 eq.) was added and incubated at 22 °C for 1 h. After that, the buffer was swapped into BBS pH 8.5 (3 x ultrafiltration) and left for 24 h at 22 °C. Lastly, sample was desalted into HPLC grade water (7 kDa MWCO, ZebaSpin) prior to LCMS analysis. Concentration was determined photometrically using ε_{280} = 68590 M^{-1} cm^{-1}.

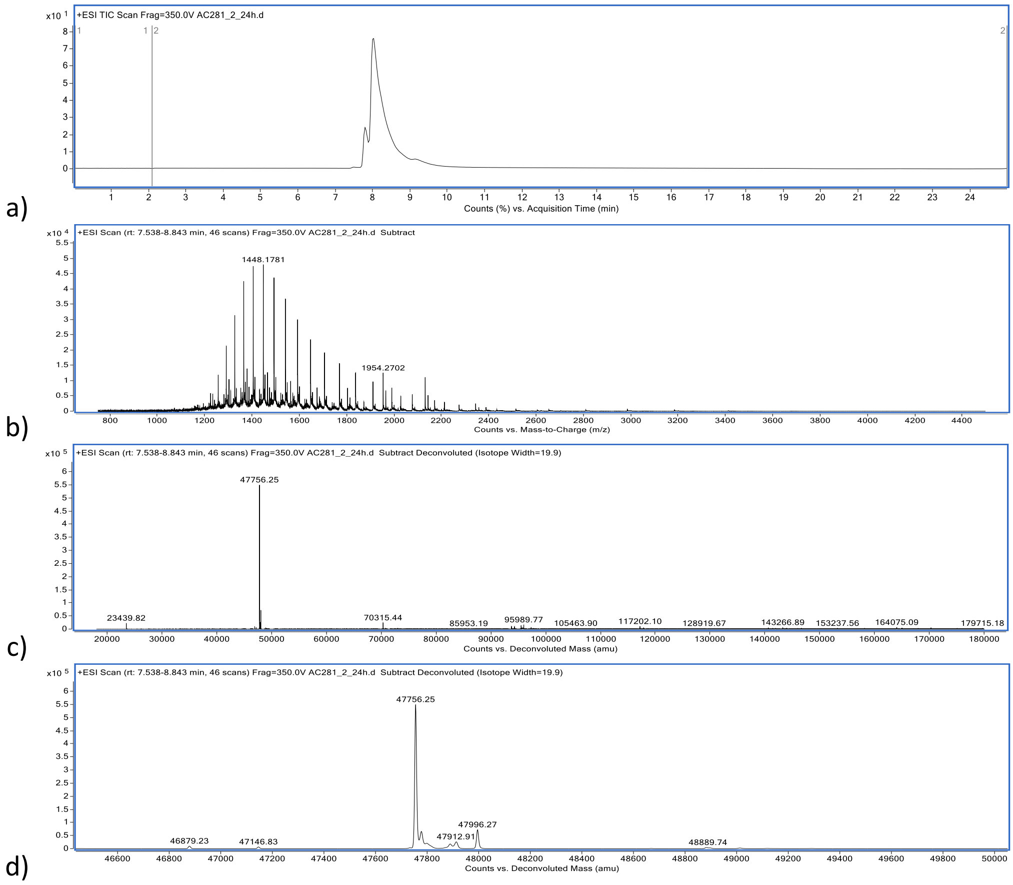

Figure S19. Stability study of Fab conjugate **7** at pH 8.5. a) TIC, b) non-deconvoluted ion-series c) full range deconvoluted ion series mass spectrum, d) zoomed in deconvoluted ion-series of LC and HC at 24 h. **S25** (LC) expected 23438, observed 23439, **S26** (LC) expected 23573, not observed, **S26** (HC) expected 24200, not observed, **S25** (HC) expected 24335, not observed, Fab conjugate **7** expected 47756, observed 47756.

Reaction of Fab with methyl 2-((2-fluoro-5-nitrobenzoyl)thio)acetate (4)

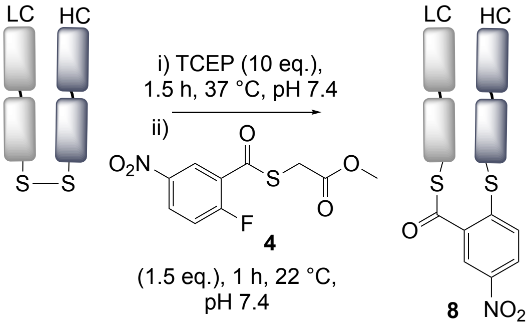

Fab (30 µL, 100 µM, 4.76 mg/mL) in conjugation buffer was reduced with tris(2-carboxyethyl)phosphine (TCEP) (2.0 µL, 15 mM in diH_2O, 10 eq.). The mixture was incubated at 37 °C for 1.5 h, 300 rpm. Following that, methyl 2-((2-fluoro-5-nitrobenzoyl)thio)acetate **4** (0.2 µl, 23 mM in DMF, 1.5 eq.) was added and incubated at 22 °C for 1 h. Lastly, sample was desalted into HPLC grade water (7 kDa MWCO, ZebaSpin) prior to LCMS analysis. Concentration was determined photometrically using ε_{280} = 68590 M^{-1} cm^{-1}.

a)
b)
c)
d)

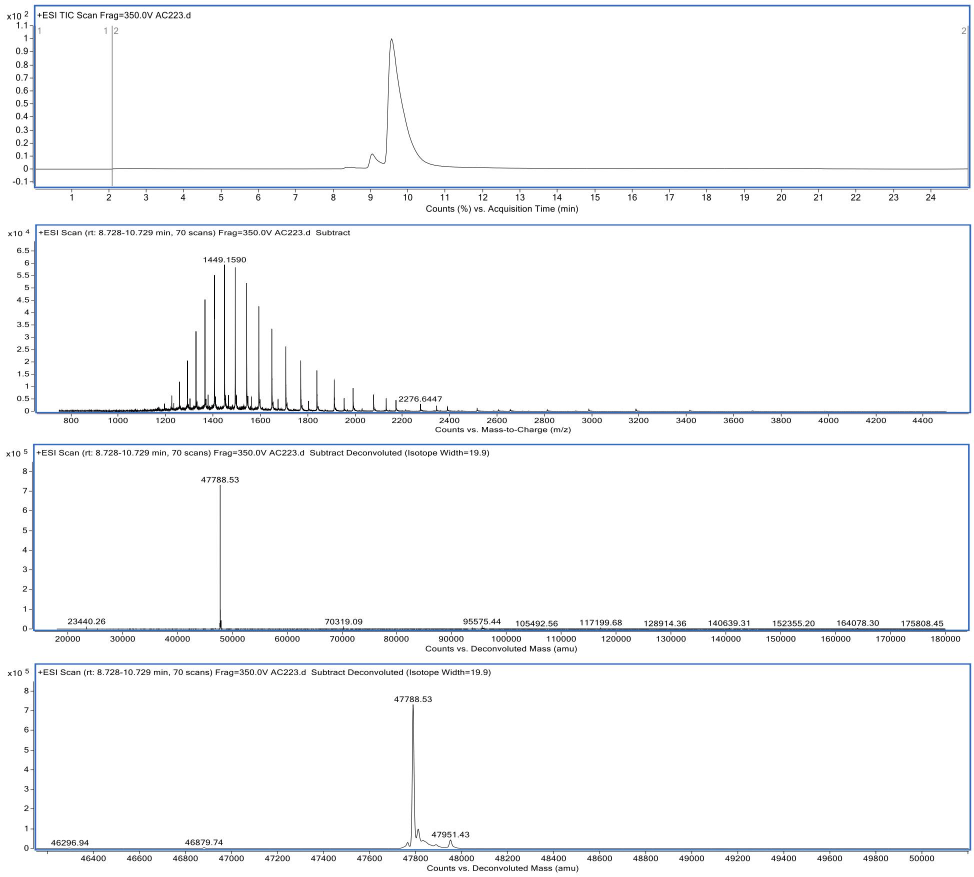

Figure S20. LCMS analysis of rebridging Fab with methyl 2-((2-fluoro-5-nitrobenzoyl)thio)acetate **4**; a) TIC, b) non-deconvoluted ion-series, c) full range deconvoluted ion series mass spectrum, d) zoomed in deconvoluted ion series mass spectrum; rebridged Fab with methyl 2-((2-fluoro-5-nitrobenzoyl)thio)acetate **4** expected 47787, observed 47788.

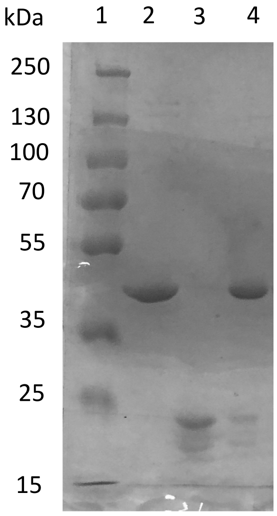

Figure S21. SDS-PAGE of rebridged Fab with methyl 2-((2-fluoro-5-nitrobenzoyl)thio)acetate **4**; 1 – molecular marker, 2 – Native Fab, 3 – Reduced Fab, 4 – rebridged Fab with **4**.

Reaction of Fab conjugate 8 with cysteine and *N*-Me-maleimide

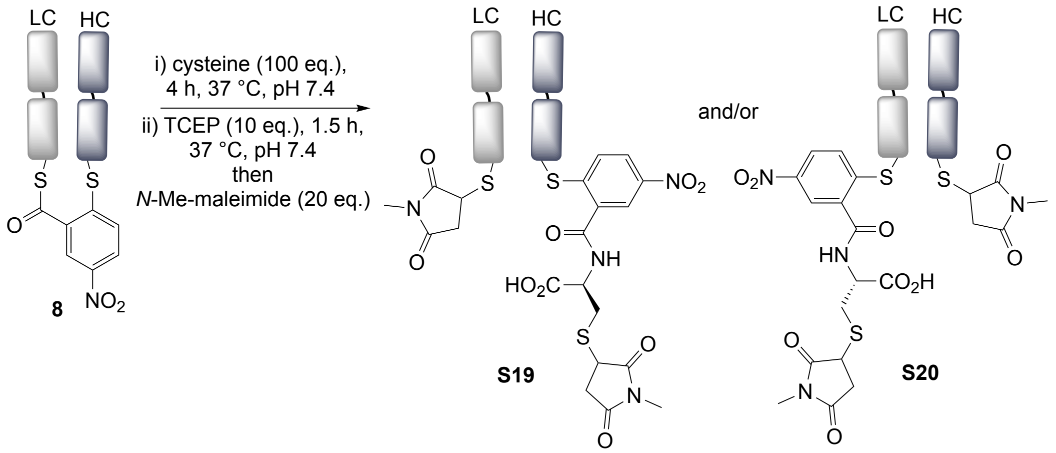

Fab (30 µL, 100 µM, 4.76 mg/mL) in conjugation buffer was reduced with tris(2-carboxyethyl)phosphine (TCEP) (2.0 µL, 15 mM in diH_2O, 10 eq.). The mixture was incubated at 37 °C for 1.5 h, 300 rpm. Following that, methyl 2-((2-fluoro-5-nitrobenzoyl)thio)acetate **4** (0.2 µl, 23 mM in DMF, 1.5 eq.) was added and incubated at 22 °C for 1 h. After that, L-cysteine (2.7 µL, 108 mM in diH_2O, 100 eq.) was added and left for at 37 °C, for 4 h, 300 rpm. After this period, the excess reagent was removed *via* ultrafiltration (10 kDa MWCO), into conjugation buffer, new Fab concentration was determined, and TCEP reduction was performed again as before. To cap the free thiols, an excess of *N*-methylmaleimide was added (39 mM in diH_2O, 20 eq.). Lastly, sample was desalted (7 kDa MWCO, ZebaSpin) prior to LCMS analysis. Concentration was determined photometrically using ε_{280} = 68590 M^{-1} cm^{-1}.

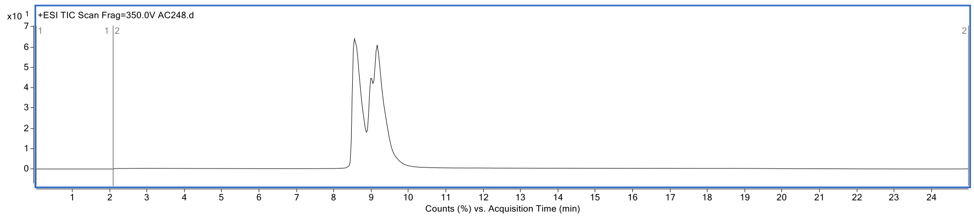

a)

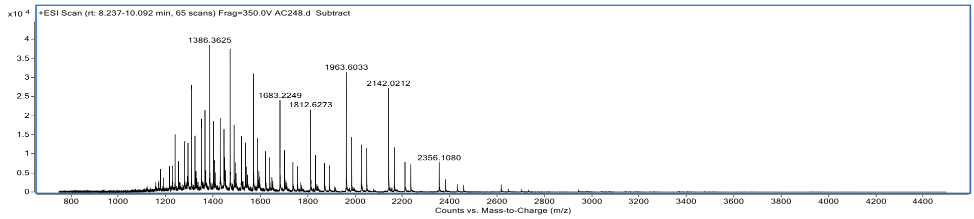

b)

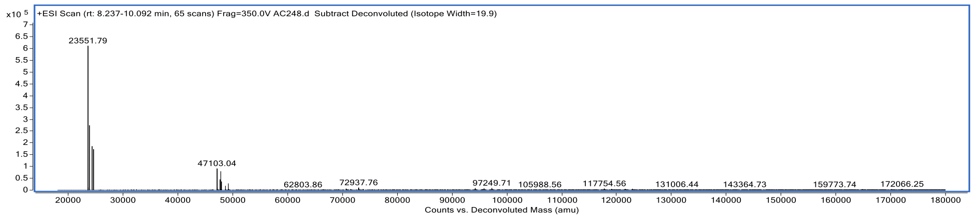

c)

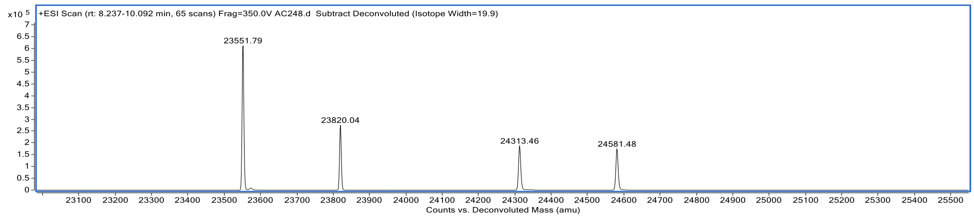

d)

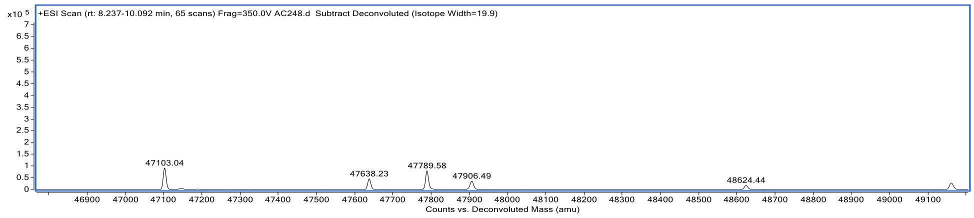

e)

Figure S22. LCMS analysis of regioselectivity preference of methyl 2-((2-fluoro-5-nitrobenzoyl)thio)acetate **4**; a) TIC, b) non-deconvoluted ion-series, c) full range deconvoluted ion series mass spectrum, d) zoomed in deconvoluted ion series mass spectrum; **S19** (LC) expected 23550, observed 23551, **S20** (LC) expected 23818, observed 23820, **S20** (HC) expected 24312, observed 24313, **S19** (HC) expected 24580, observed 24581, e) zoomed in deconvoluted ion series mass spectrum; rebridged Fab with methyl 2-((2-fluoro-5-nitrobenzoyl)thio)acetate **4** expected 47787, observed 47789. Peaks 47103 (2 x **S19** (LC)), and 48624 (2 x **S19** (HC)) are proposed to be artifacts formed during ionisation.

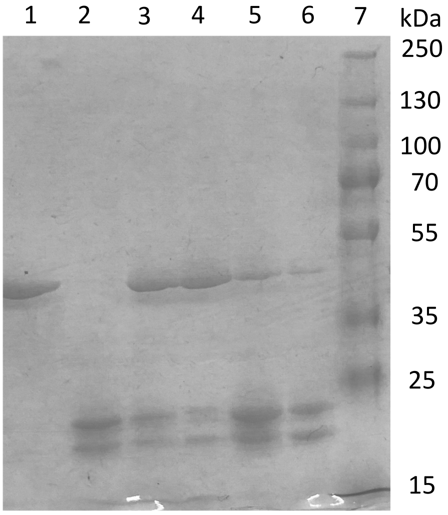

Figure S23. SDS-PAGE analysis of regioselectivity preference of methyl 2-((2-fluoro-5-nitrobenzoyl)thio)acetate **4** with cysteine; 1 – Native Fab, 2 – Reduced Fab, 3 – rebridged Fab with **4**, 4 – treatment of rebridged Fab with cysteine, 5 – 2nd TCEP reduction, 6 – *N*-Me-maleimide capped, 7 – molecular marker.

Stability study of Fab conjugate 8

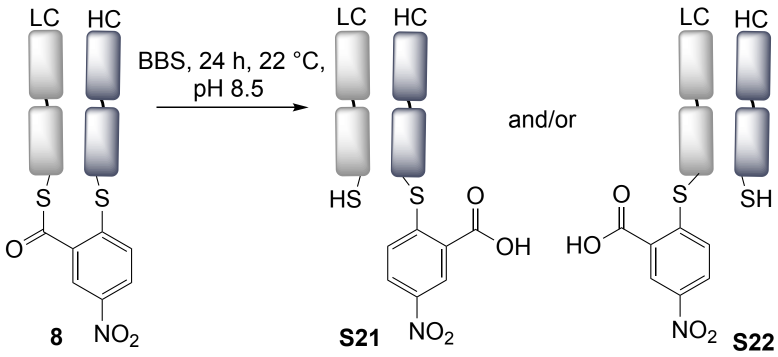

Fab (30 µL, 100 µM, 4.76 mg/mL) in conjugation buffer was reduced with tris(2-carboxyethyl)phosphine (TCEP) (2.0 µL, 15 mM in diH_2O, 10 eq.). The mixture was incubated at 37 °C for 1.5 h, 300 rpm. Following that, methyl 2-((2-fluoro-5-nitrobenzoyl)thio)acetate **4** (0.2 µl, 23 mM in DMF, 1.5 eq.) was added and incubated at 22 °C for 1 h. After that, the buffer was swapped into BBS pH 8.5 (3 x ultrafiltration) and left for 24 h at 22 °C. Lastly, sample was desalted into HPLC grade water (7 kDa MWCO, ZebaSpin) prior to LCMS analysis. Concentration was determined photometrically using ε_{280} = 68590 M^{-1} cm^{-1}.

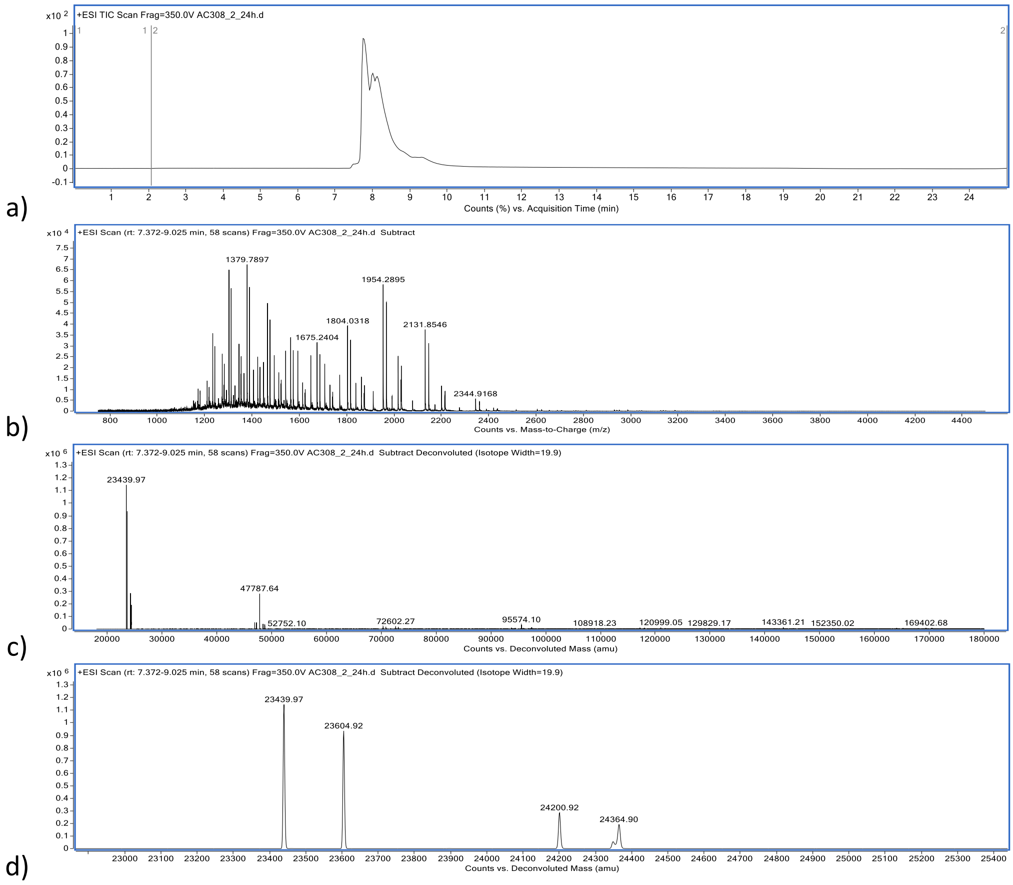

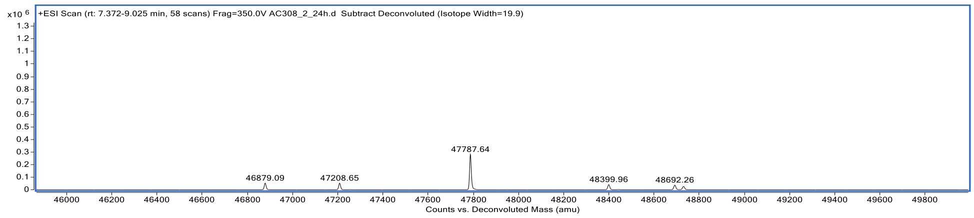

e)

Figure S24. Stability study of Fab conjugate **8** at pH 8.5. a) TIC, b) non-deconvoluted ion-series, c) full range deconvoluted ion series mass spectrum, d) zoomed in deconvoluted ion-series of LC and HC at 24 h; **S21** (LC) expected 23438, observed 23439, **S22** (LC) expected 23604, observed 23604, **S22** (HC) expected 24200, observed 24200, **S21** (HC) expected 24366, observed 24364, e) deconvoluted ion-series of Fab rebridged with **4** expected 47787, observed 47787. Peaks 46879 (2 x **S21** (LC)), 47208 (2 x **S22** (LC)), 48399 (2 x **S22** (HC)), 48692 (2 x **S21** (HC)) are proposed to be artifacts formed during ionisation.

Reaction of Fab with α-chlorothioester (1) and TAT peptide

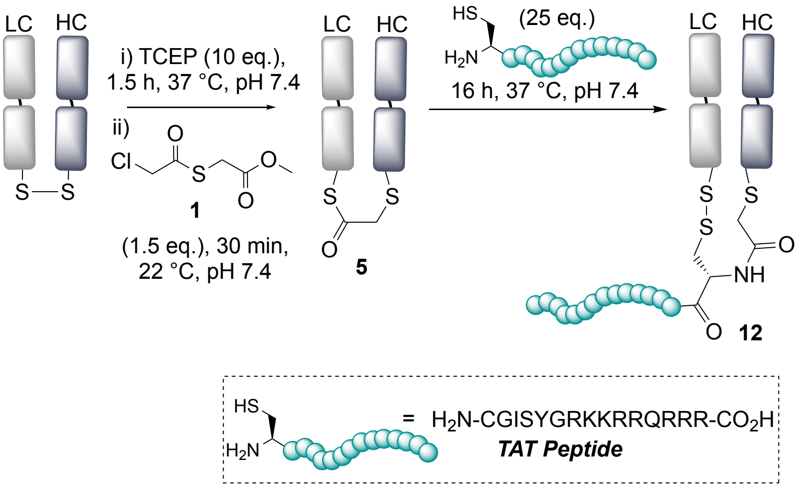

Fab (30 µL, 100 µM, 4.76 mg/mL) in conjugation buffer was reduced with tris(2-carboxyethyl)phosphine (TCEP) (2.0 µL, 15 mM in diH_2O, 10 eq.). The mixture was incubated at 37 °C for 1.5 h, 300 rpm. Following that, α-chlorothioester **1** (0.2 µl, 24 mM in DMF, 1.5 eq.) was added and incubated at 22 °C for 30 min. After that, TAT peptide (7.5 µL, 10 mM in diH_2O and 15 % MeCN, 25 eq.) was added and left at 37 °C for 16 h, 300 rpm. Lastly, sample was desalted into HPLC grade water (7 kDa MWCO, ZebaSpin) prior to LCMS analysis. Concentration was determined photometrically using ε_{280} = 68590 M^{-1} cm^{-1}.

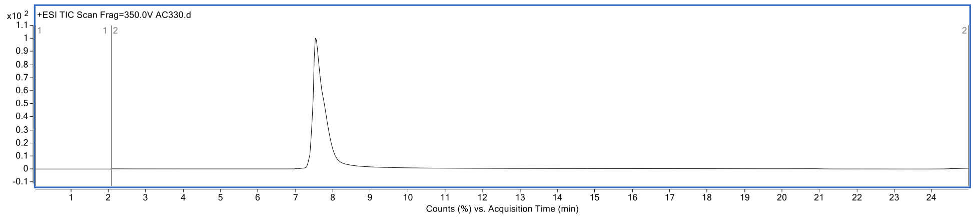

a)

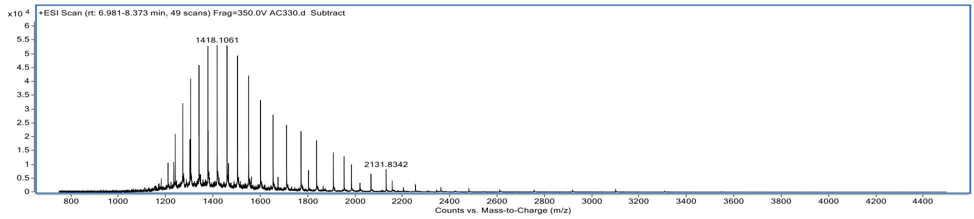

b)

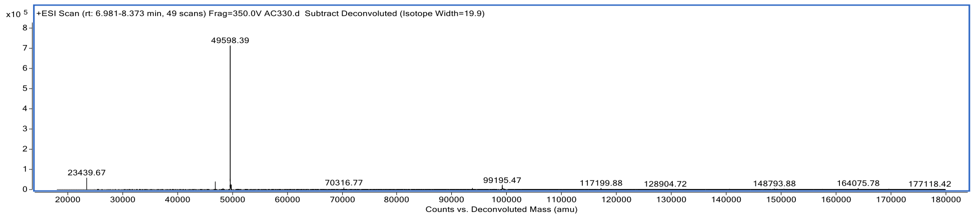

c)

Figure S25. LCMS analysis of TAT peptide conjugate **12**; a) TIC, b) non-deconvoluted ion-series, c) full range deconvoluted ion series mass spectrum; Fab conjugate **12** with TAT peptide expected 49600, observed 49598.

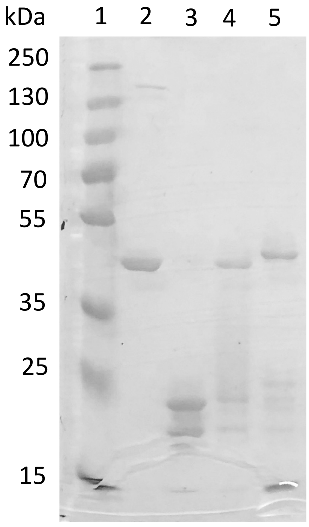

Figure S26. SDS-PAGE of TAT peptide conjugate **12**; 1 – molecular marker, 2 – Native Fab, 3 – Reduced Fab, 4 – rebridged Fab with **1**, 5 – TAT peptide.

Reaction of Fab with α-chlorothioester (1) with TAT peptide and BCN-PD

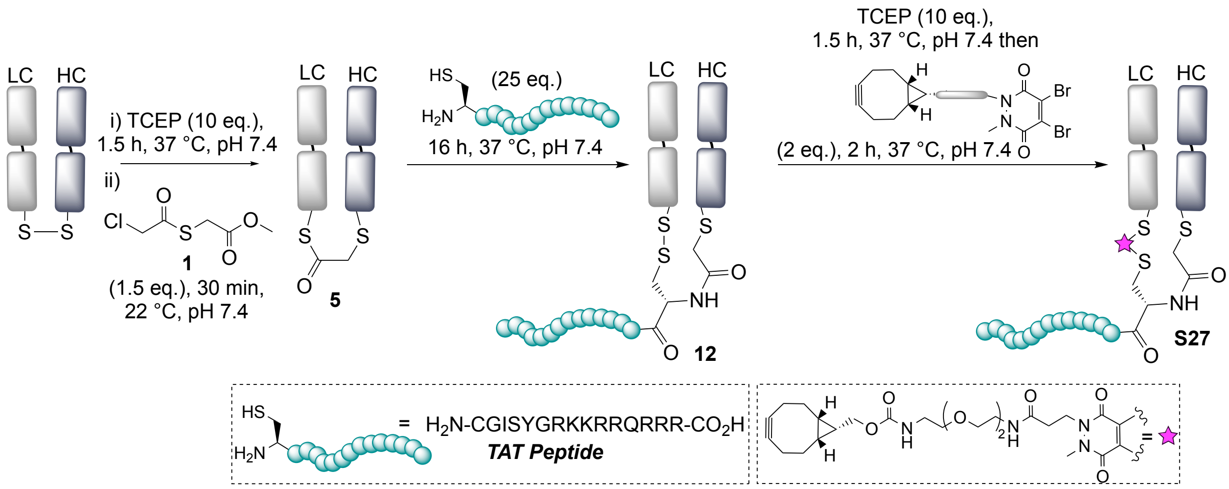

Fab (30 µL, 100 µM, 4.76 mg/mL) in conjugation buffer was reduced with tris(2-carboxyethyl)phosphine (TCEP) (2.0 µL, 15 mM in diH_2O, 10 eq.). The mixture was incubated at 37 °C for 1.5 h, 300 rpm. Following that, α-chlorothioester **1** (0.2 µl, 24 mM in DMF, 1.5 eq.) was added and incubated at 22 °C for 30 min. After that, TAT peptide (7.5 µL, 10 mM in diH_2O and 15 % MeCN, 25 eq.) was added and left at 37 °C for 16 h, 300 rpm. Upon completion of this step, excess of the reagents was removed *via* ultrafiltration (10 kDa MWCO) into conjugation buffer, new Fab concentration was determined. TCEP reduction was performed again as before followed by removal of the excess of the reagent. Subsequently, BCN-PD (20 mM in DMSO, 2 eq.) was added and left at 37 °C for 2 h, 300 rpm. This was followed by another ultrafiltration to remove excess of the reagent. Lastly, sample was desalted into HPLC grade water (7 kDa MWCO, ZebaSpin) prior to LCMS analysis. Concentration was determined photometrically using ε_{280} = 68590 M^{-1} cm^{-1}.

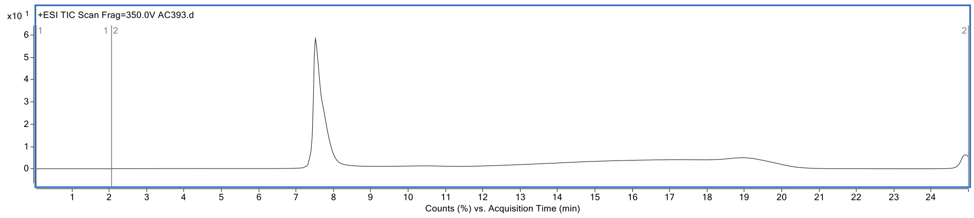

a)

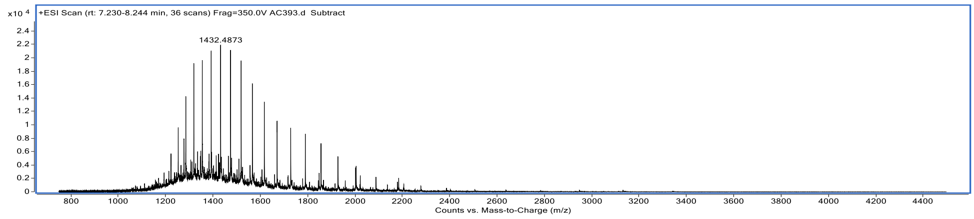

b)

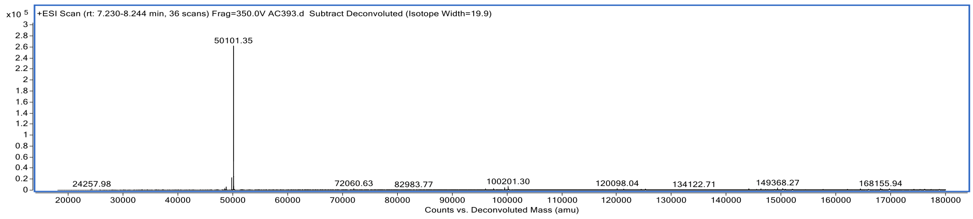

c)

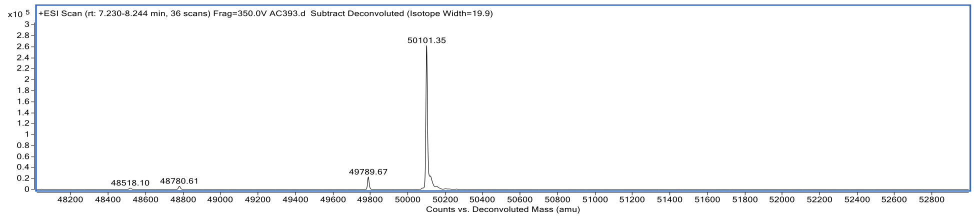

d)

Figure S27. LCMS analysis of conjugate **S27**; a) TIC, b) non-deconvoluted ion-series, c) full range deconvoluted ion series mass spectrum, d) zoomed in deconvoluted ion series mass spectrum; Fab conjugate **S27** with TAT peptide and BCN-PD expected 50102, observed 50101. The minor peak 49789 is undefined, but it is notably not present, and therefore not associated with, the key TAT peptide conjugation step.

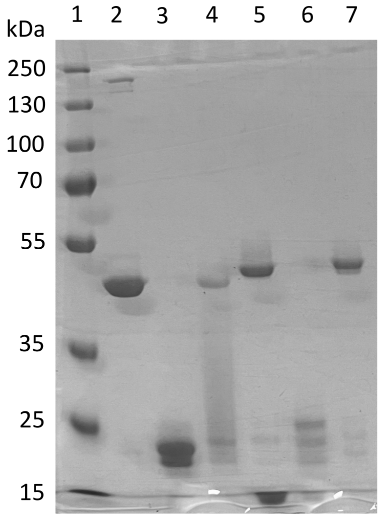

Figure S28. SDS-PAGE analysis of conjugate **S27**; 1 – molecular marker, 2 – Native Fab, 3 – Reduced Fab, 4 – rebridged Fab with **1**, 5 – TAT peptide, 6 – 2nd TCEP reduction, 7 – BCN-PD. Calculated densitometry analysis shows >90% formation of Fab conjugate.

Reaction of Fab with α-chlorothioester (1) with TAT peptide, BCN-PD and Azide-fluor 488

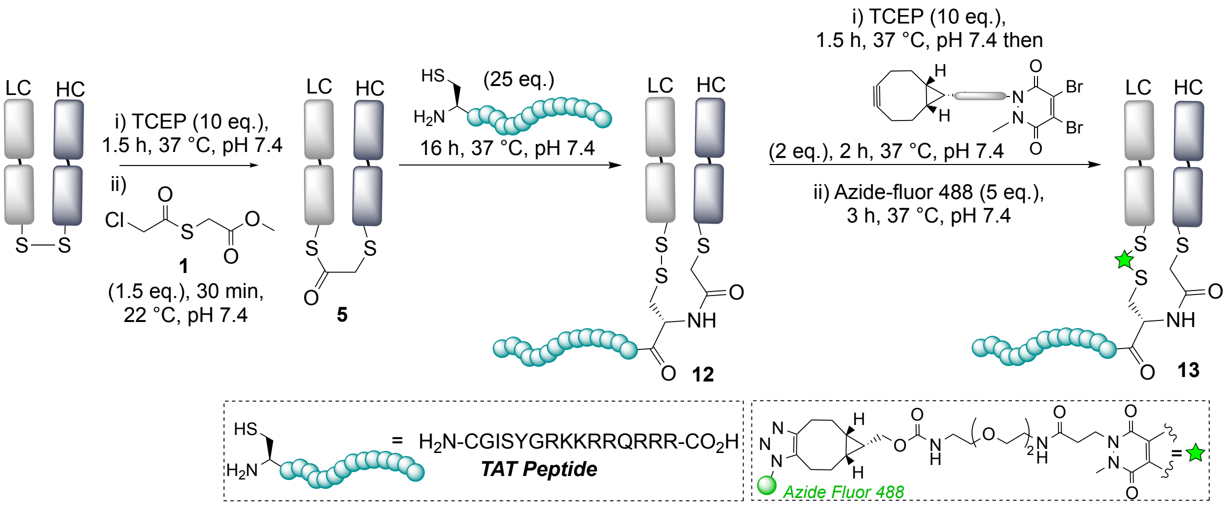

Fab (26 µL, 100 µM, 4.76 mg/mL) in conjugation buffer was reduced with tris(2-carboxyethyl)phosphine (TCEP) (1.7 µL, 15 mM in diH_2O, 10 eq.). The mixture was incubated at 37 °C for 1.5 h, 300 rpm. Following that, α-chlorothioester **1** (0.2 µl, 24 mM in DMF, 1.5 eq.) was added and incubated at 22 °C for 30 min. After that, TAT peptide (6.5 µL, 10 mM in diH_2O and 15 % MeCN, 25 eq.) was added and left at 37 °C for 16 h, 300 rpm. Upon completion of this step, excess of the reagents was removed *via* ultrafiltration (10 kDa MWCO) into conjugation buffer, new Fab concentration was determined. TCEP reduction was performed on conjugate **12** as before followed by removal of the excess of the reagent. Subsequently, BCN-PD (20 mM in DMSO, 2 eq.) was added and left at 37 °C for 2 h, 300 rpm. This was followed by another ultrafiltration to remove excess of the reagent. Finally, Azide-fluor 488 (10 mM in DMF, 5 eq.) was added and this was left in the dark at 37 °C for 3 h, 300 rpm. The excess reagent was then removed using a desalting column (PD Minitrap G-25, GE Healthcare) followed by ultrafiltration (10 kDa MWCO) into conjugate buffer to concentrate the sample. Lastly, sample was desalted into HPLC grade water (7 kDa MWCO, ZebaSpin) prior to LCMS analysis. Concentration was determined photometrically using ε_{280} = 68590 M^{-1} cm^{-1}. The sample was then analysed by UV/Vis spectroscopy and SDS-PAGE.

The fluorophore-to-antibody ratio (FAR) was the determined using the following formula, where *Cf* is the correction factor for the absorbance of Azide-fluor 488 at 280 nm and 505 nm:

$$n = \frac{\varepsilon_{280}\ x\ Abs_{335}}{\varepsilon_{335}\ x\ (Abs_{280} - Cf\ x\ Abs_{335} - Cf\ x\ Abs_{505})}$$

$$n = \frac{68590\ x\ 0.040}{9100\ x\ (0.260 - (0.25\ x\ 0.040) - (0.11\ x\ 0.210))} = 1.32$$

$$FAR = \frac{n\ x\ \varepsilon_{335}\ x\ Abs_{505}}{\varepsilon_{505}\ x\ Abs_{335}} = \frac{1.32\ x\ 9100\ x\ 0.210}{74000\ x\ 0.040} = 0.9$$

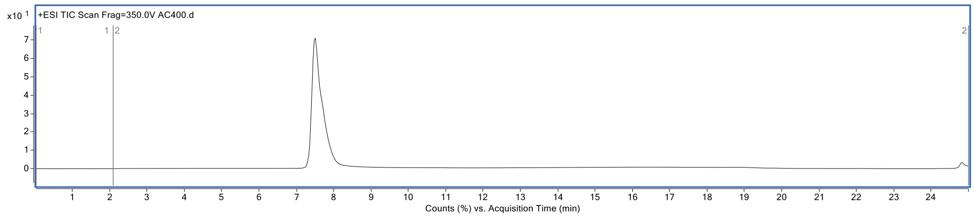

a)

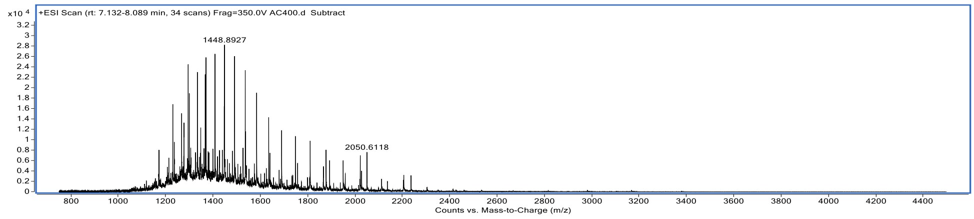

b)

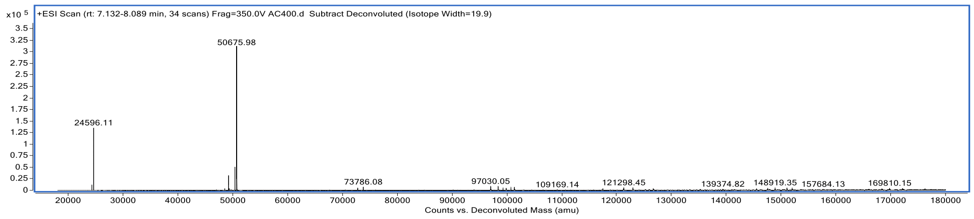

c)

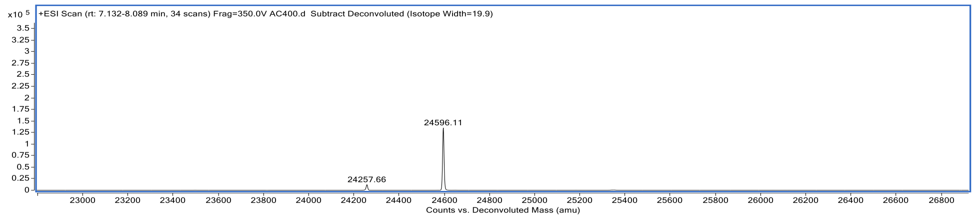

d)

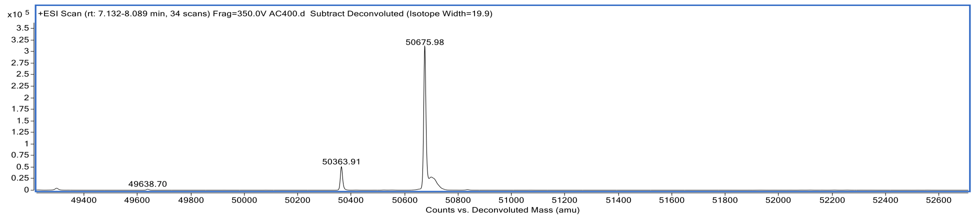

e)

Figure S29. LCMS analysis of conjugate **13**; a) TIC, b) non-deconvoluted ion-series, c) full range deconvoluted ion series mass spectrum, d) and e) zoomed in deconvoluted ion series mass spectrum; HC with hydrolysed rebridging species expected 24259, observed 24257; LC with BCN-PD-Br and Azide-fluor 488 expected 24594, observed 24596; Fab conjugate **13** with TAT peptide, BCN-PD and Azide-fluor 488 expected 50677, observed 50675. The peak 24596 shows relatively high intensity on the MS spectra, however, this can be accounted for less than 10% measured by densitometry on the SDS-PAGE analysis. The minor peak 50363 is undefined, but it is notably not present, and therefore not associated with, the key TAT peptide conjugation step.

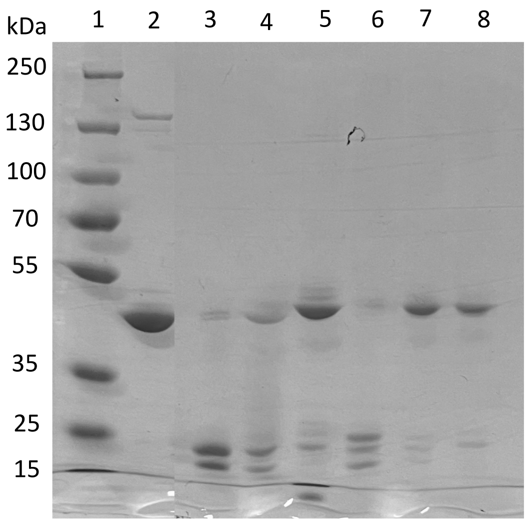

Figure S30. SDS-PAGE of Fab analysis of conjugate **13**; 1 – molecular marker, 2 – Native Fab, 3 – Reduced Fab, 4 – rebridged Fab with **1**, 5 – TAT peptide, 6 – 2nd TCEP reduction, 7 – BCN-PD, 8 – Azide-fluor 488. Calculated densitometry analysis shows >88% formation of Fab conjugate.

Reaction of Fab with α-chlorothioester (1), hydrazine hydrate, pyridyldisulfide and Azide-Fluor 488

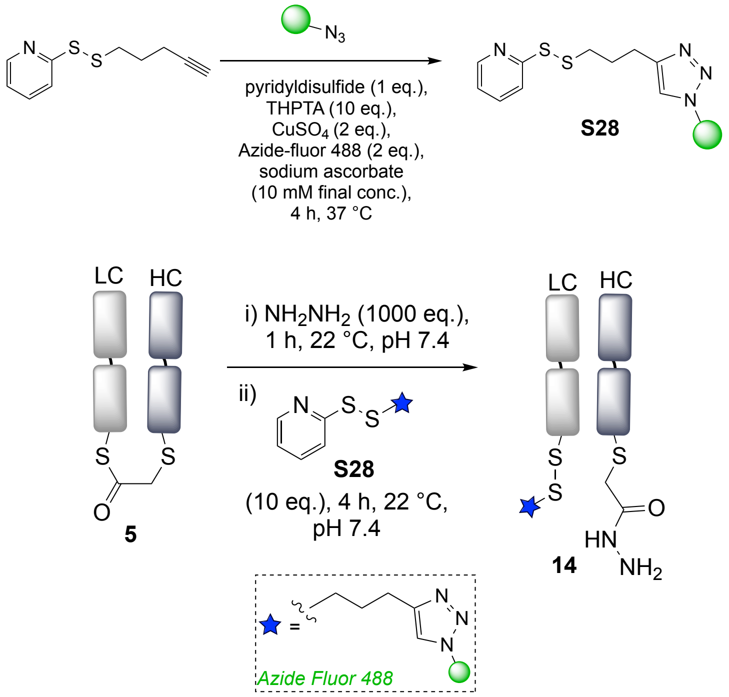

To a solution of the pyridyldisulfide alkyne (0.3 µL, 81 mM in DMF, 1 eq.), THPTA (2.5 µL, 100 mM in diH_2O, 10 eq.), $CuSO_4$ (2.5 µL, 20 mM in diH_2O, 2 eq.), Azide-fluor 488 (5 µL, 10 mM in DMF, 2 eq.), and sodium ascorbate (1.2 µL, 100 mM in diH_2O, final conc. 10 mM) were added and stirred at 37 °C for 4 h, 300 rpm to form **S28**. In meantime, Fab (25 µL, 100 µM, 4.76 mg/mL) in conjugation buffer was reduced

with tris(2-carboxyethyl)phosphine (TCEP) (1.7 µL, 15 mM in diH_2O, 10 eq.). The mixture was incubated at 37 °C for 1.5 h, 300 rpm. Following that, α-chlorothioester **1** (0.2 µl, 24 mM in DMF, 1.5 eq.) was added and incubated at 22 °C for 30 min. Followed by addition of hydrazine hydrate (1.1 µL, 2.2 M in DMF, 1000 eq.) at 22 °C, 1 h, 300 rpm and ultrafiltration into conjugation buffer. After this, **S28** was added to the Fab conjugate and this was further incubated at 22 °C for 4 h, 300 rpm. The excess reagent was then removed using a desalting column (PD Minitrap G-25, GE Healthcare) followed by ultrafiltration (10 kDa MWCO) into conjugate buffer to concentrate the sample. Lastly, sample was desalted into HPLC grade water (7 kDa MWCO, ZebaSpin) prior to LCMS analysis. Concentration was determined photometrically using ε_{280} = 68590 M^{-1} cm^{-1}. The sample was then analysed by UV/Vis spectroscopy and SDS-PAGE.

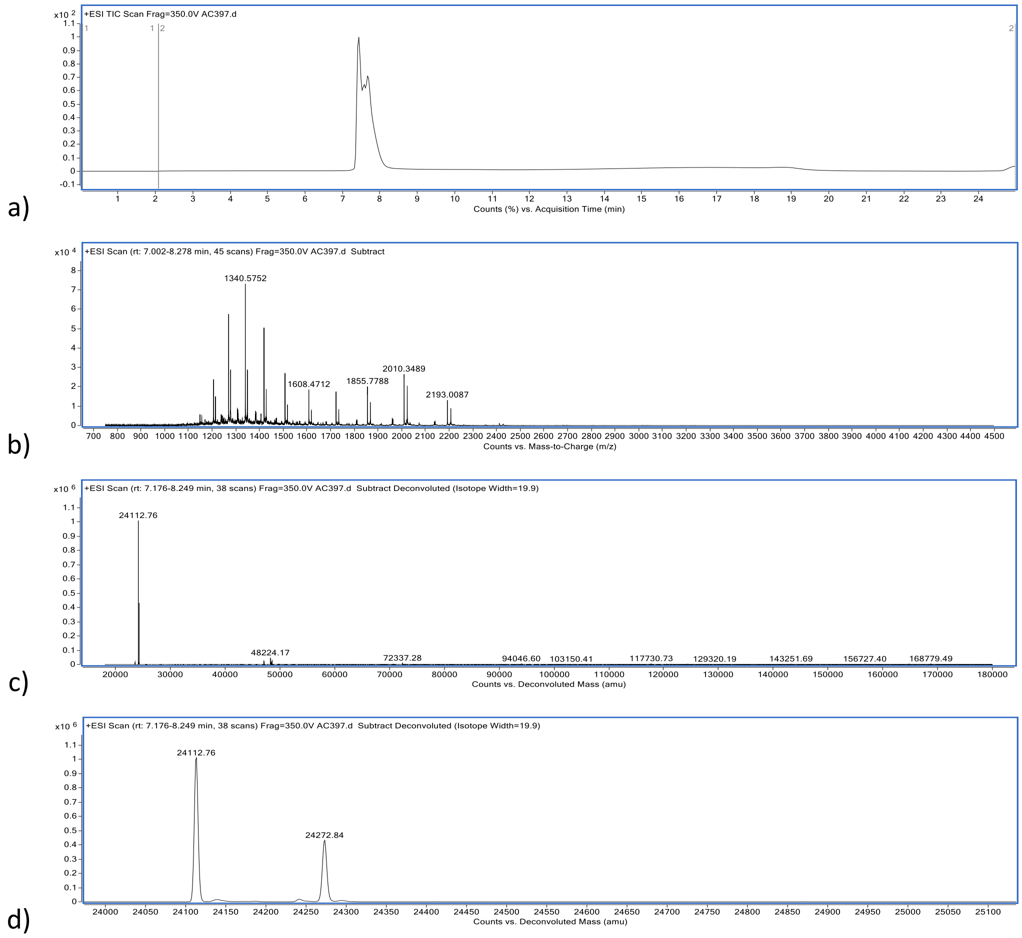

Figure S31. LCMS analysis of conjugate **14**; a) TIC, b) non-deconvoluted ion-series, c) full range deconvoluted ion series mass spectrum, d) zoomed in deconvoluted ion series mass spectrum; **14** (LC) expected 24112, observed 24112; **14** (HC) expected 24272, observed 24272.

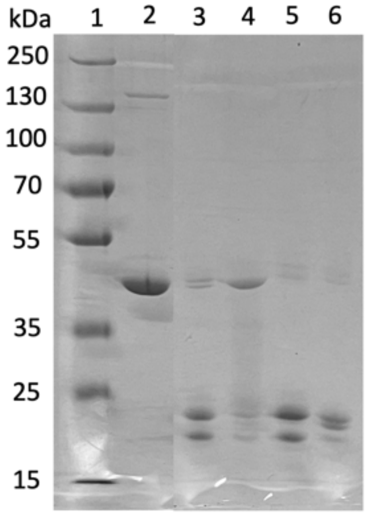

Figure S32. SDS-PAGE analysis of conjugate **14**; 1 – molecular marker, 2 – Native Fab, 3 – Reduced Fab, 4 – rebridged Fab with **1**, 5 – hydrazine hydrate, 6 – pre-click. Calculated densitometry analysis shows >90% formation of HC and LC species.

The fluorophore-to-antibody ratio (FAR) was the determined using the following formula, where *Cf* is the correction factor for the absorbance of Azide-fluor 488 at 280 nm and 505 nm:

$$FAR = \frac{\frac{Abs_{505}}{\varepsilon_{505}}}{\frac{Abs_{280} - (Cf\ x\ Abs_{505})}{\varepsilon_{280}}} = \frac{\frac{1.25}{74000}}{\frac{1.80 - (0.11\ x\ 1.25)}{68590}} = 0.7$$

Reaction of Fab with α-chlorothioester (1), hydrazine hydrate, pyridyldisulfide, Azide-fluor 488, and biotin-aldehyde

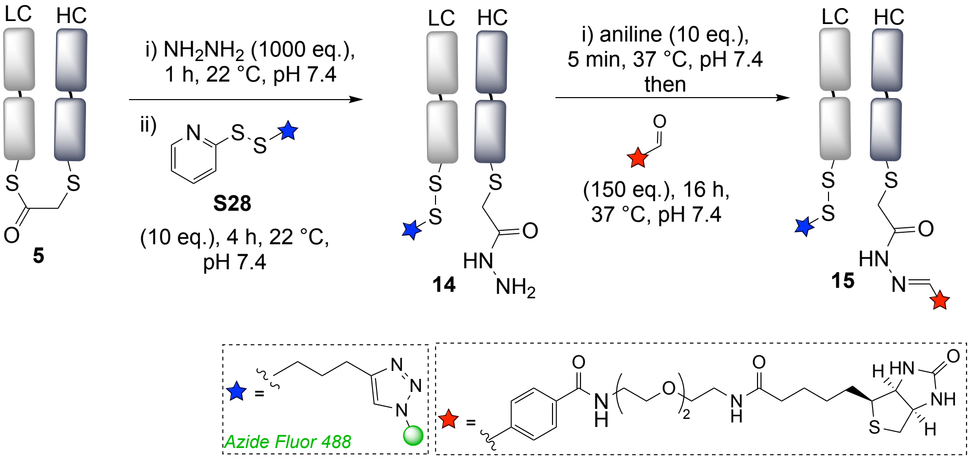

To a solution of the pyridyldisulfide alkyne (0.3 µL, 81 mM in DMF, 1 eq.), THPTA (2.5 µL, 100 mM in diH_2O, 10 eq.), $CuSO_4$ (2.5 µL, 20 mM in diH_2O, 2 eq.), Azide-fluor 488 (5 µL, 10 mM in DMF, 2 eq.), and

sodium ascorbate (1.2 µL, 100 mM in diH_2O, final conc. 10 mM) were added and stirred at 37 °C for 4 h, 300 rpm to form **S28**. In meantime, Fab (26 µL, 100 µM, 4.76 mg/mL) in conjugation buffer was reduced with tris(2-carboxyethyl)phosphine (TCEP) (1.7 µL, 15 mM in diH_2O, 10 eq.). The mixture was incubated at 37 °C for 1.5 h, 300 rpm. Following that, α-chlorothioester **1** (0.2 µl, 24 mM in DMF, 1.5 eq.) was added and incubated at 22 °C for 30 min. Followed by addition of hydrazine hydrate (1.0 µL, 2.5 M in DMF, 1000 eq.) at 22 °C, 1 h, 300 rpm and ultrafiltration into conjugation buffer. After this, **S28** was added to the Fab conjugate and this was further incubated at 22 °C for 4 h, 300 rpm. The excess reagent was then removed using a desalting column (PD Minitrap G-25, GE Healthcare) followed by ultrafiltration (10 kDa MWCO) into conjugate buffer to concentrate the sample, new Fab concentration was determined. Subsequently, aniline (370 mM in DMF, 100 eq.) was added and biotin-aldehyde (49 mM in DMF, 150 eq.), this was left at 37 °C, for 16 h, 300 rpm. Lastly, sample was desalted into HPLC grade water (7 kDa MWCO, ZebaSpin) prior to LCMS analysis. Concentration was determined photometrically using ε_{280} = 68590 M^{-1} cm^{-1}. The sample was then analysed by UV/Vis spectroscopy and SDS-PAGE.

The fluorophore-to-antibody ratio (FAR) was the determined using the following formula, where *Cf* is the correction factor for the absorbance of Azide-fluor 488 at 280 nm and 505 nm:

$$FAR = \frac{\frac{Abs_{505}}{\varepsilon_{505}}}{\frac{Abs_{280} - (Cf\ x\ Abs_{505})}{\varepsilon_{280}}} = \frac{\frac{3.04}{74000}}{\frac{3.97 - (0.11\ x\ 3.04)}{68590}} = 0.8$$

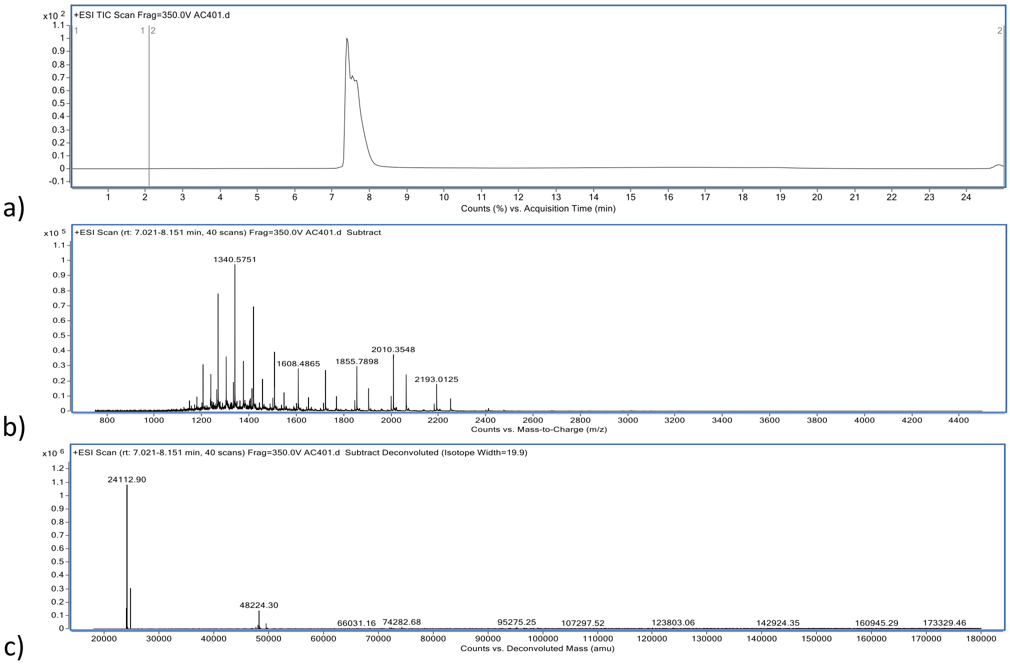

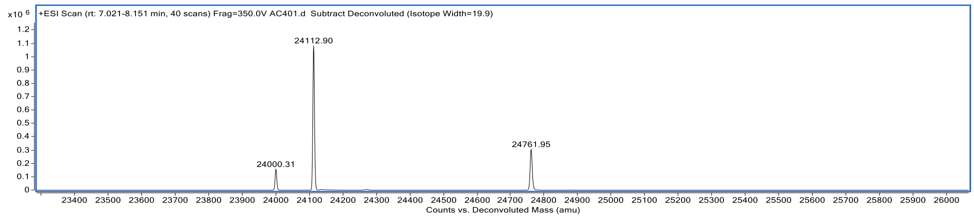

d)

Figure S33. LCMS analysis of conjugate **15**; a) TIC, b) non-deconvoluted ion-series, c) full range deconvoluted ion series mass spectrum, d) zoomed in deconvoluted ion series mass spectrum; **15** (LC) expected 24112, observed 24112, LC of minor regioisomer expected 23999, observed 24000; **15** (HC) expected 24761, observed 24761.

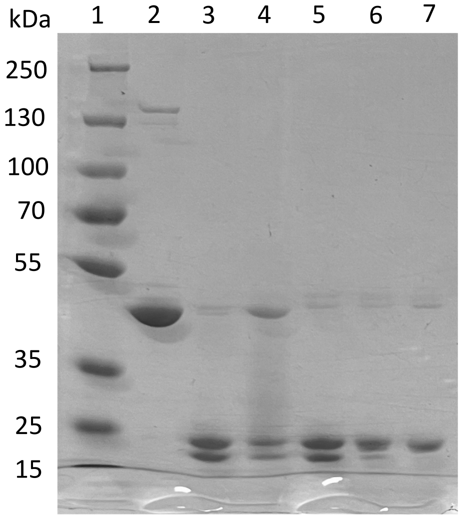

Figure S34. SDS-PAGE analysis of conjugate **15**; 1 – molecular marker, 2 – Native Fab, 3 – Reduced Fab, 4 – rebridged Fab with **1**, 5 – hydrazine hydrate, 6 – pre-click, 7 – biotin-aldehyde. Calculated densitometry analysis shows >90% formation of HC and LC species.

Dual conjugation – disulfide cleavage

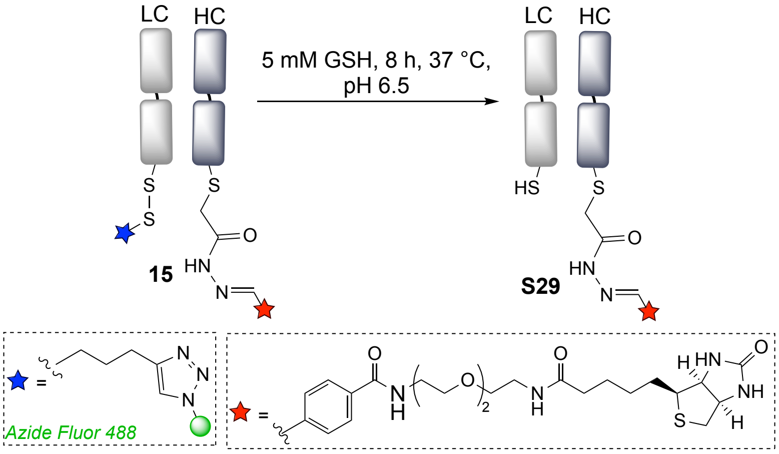

Upon forming the dual conjugate **15** as described in section above, the conjugate was then buffer swapped into 5 mM glutathione (GSH) in PB, pH 6.5 and it was left at 37 °C, for 8 h, 300 rpm. Lastly,

sample was desalted into HPLC grade water (7 kDa MWCO, ZebaSpin) prior to LCMS analysis. Concentration was determined photometrically using ε_{280} = 68590 M^{-1} cm^{-1}. The sample was then analysed by UV/Vis spectroscopy and SDS-PAGE.

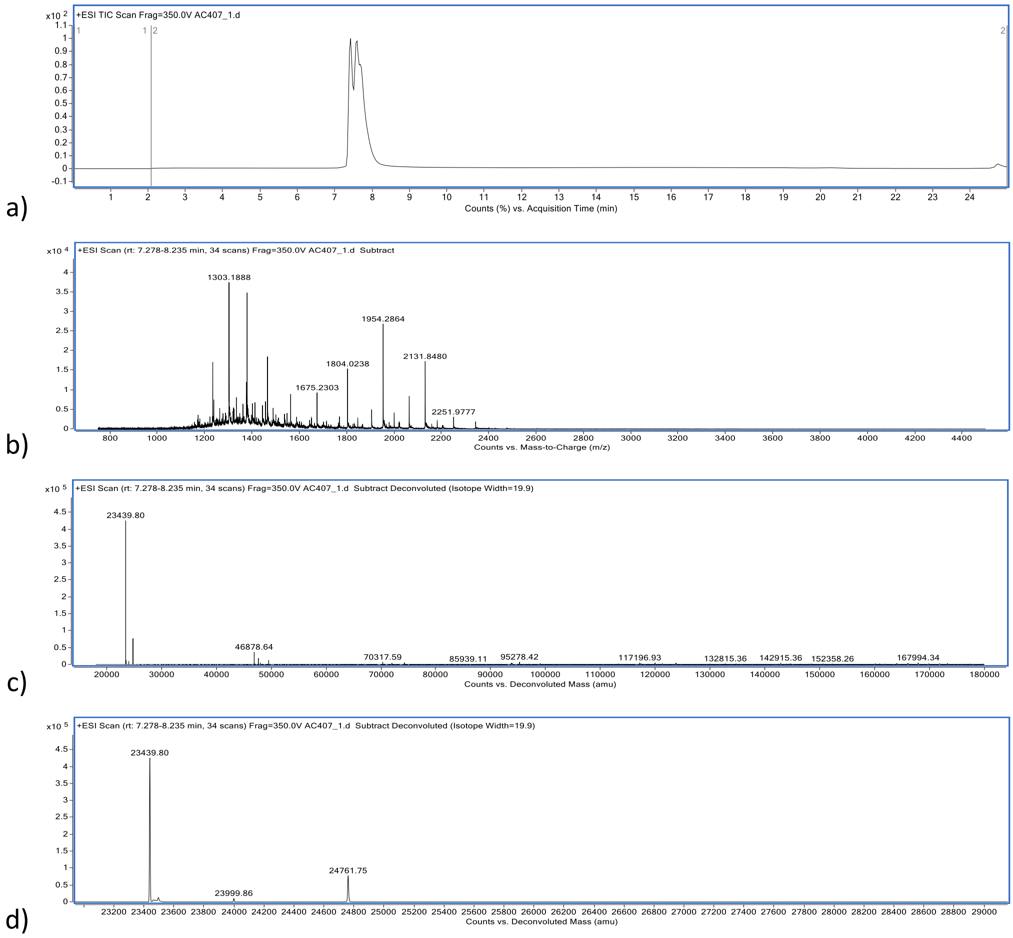

Figure S35. LCMS analysis of conjugate **S29**; a) TIC, b) non-deconvoluted ion-series, c) full range deconvoluted ion series mass spectrum, d) zoomed in deconvoluted ion series mass spectrum; **S29** (LC) expected 23438, observed 23439; LC of minor regioisomer expected 23999, observed 23999; **S29** (HC) expected 24761, observed 24761.

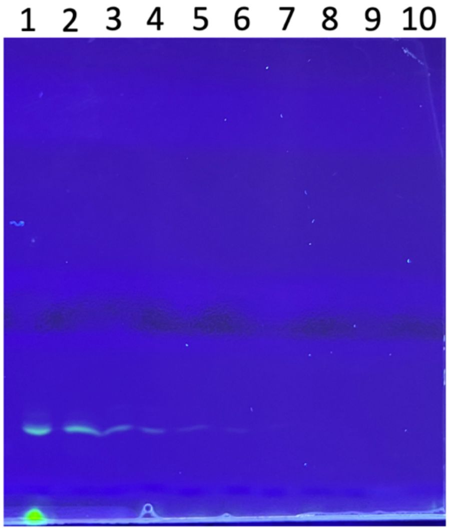

Figure S36. SDS-PAGE analysis of conjugate **S29**: 1 – pre-click, 2 – biotin-aldehyde, 3 – GSH 1 h, 4 – GSH 2 h, 5 – GSH 3 h, 6 – GSH 4 h, 7 – GSH 5 h, 8 – GSH 6 h, 9 – GSH 7 h, 10 – GSH 8 h. Calculated densitometry analysis shows 52% decrease of fluorescence in the 1 h, 72% decrease of fluorescence in the 2 h, 86% decrease of fluorescence in 3 h, 92% decrease of fluorescence in 4 h, and 100% decrease of fluorescence in lanes 7-10.

Enzyme-linked immunosorbent assay (ELISA) – Trastuzumab against HER2

A 96-well plate was coated for 16 h at 4 °C with HER2 (Sino Biological, 100 µL/well, 0.25 µg/mL solution in PBS), all wells except row D and H. After washing (3 × 0.1% Tween® 20 in PBS, followed by 3 × PBS), wells were blocked for 1 h at room temperature with 5% Marvel milk powder (Premier foods) in PBS (200 µL/well). The wells were then washed (3 × 0.1% Tween® 20 in PBS, followed by 3 × PBS), and the following dilutions of Fab conjugate were applied: 810 nM, 270 nM, 90 nM, 30 nM, 10 nM, 3.33 nM, 1.11 nM, 0.37 nM, 0.123 nM, 0.0412 nM, 0.0137 nM, prepared in 1% Marvel solution in PBS (100 µL/well), these were added to rows A to C – 1-11 and E to G – 1-11. The assay was then incubated at room temperature for 1 h. After 1 h, the plate was washed (3 × 0.1% Tween® 20 in PBS, followed by 3 × PBS), and the detection antibody (Anti-Human IgG, Fab specific-horseradish peroxidase (HRP) antibody, Sigma Aldrich, 1:5000 in 1% Marvel solution in 0.1% Tween® 20 in PBS) was added to the whole plate (100 µL/well), and incubated for 1 h at room temperature. After that, the plate was washed (3 × 0.1% Tween® 20 in PBS, followed by 3 × PBS), and *o*-phenylenediamine hydrochloride (Sigma-Aldrich, 100 µL/well, 0.5 mg/mL in a phosphate-citrate buffer with sodium perborate) was added to the whole plate, left for 15 – 30 min in dark (monitor the colour development), room temperature. Once a yellow-orange colour was observed, the reaction was stopped by addition of HCl to the whole plate (4 M, 50 µL/well).

Absorbance was immediately measured at 450 nm and was corrected by subtracting the average of negative controls (i.e. PBS had been added to some of the wells instead of HER2 or instead of the

samples). Each sample was tested in triplicate and errors are shown as the standard deviation of the average. ELISA data was analysed with Graphpad Prism 7.03 (using equation Sigmoidal, 4PL, X is log(concentration)) and the values have been normalised.

Thermal Shift Assay

Melting temperature (T_m) of trastuzumab Fab conjugates was determined by thermal melt using Eppendorf qPCR equipment and Mastercycler software. Trastuzumab Fab conjugates were diluted to 4 µM in conjugation buffer pH 7.4 to give a final volume of 10 µL. SYPRO™ orange (Thermo Fischer) (1 µL, diluted 1:500 in conjugation buffer pH 7.4) was added to each sample before transferring to a 96-well PCR plate (Fischer Sci). Samples were heated in the range of 25 – 95 °C with ramping temperature at 1 °C/min. Thermal shift curves were analysed using Graph Pad Prism software to determine T_m (midpoint) values.

References

1 L. Benati, G. Calestani, R. Leardini, M. Minozzi, D. Nanni, P. Spagnolo and S. Strazzari, *Org. Lett.*, 2003, **5**, 1313–1316.

2 Z. Fang, B. Jiang, W. Wu, Z. Xiang, C. Ouyang, T. Huang, J. Chen and L. Zeng, *Chem. Commun.*, 2013, **49**, 6164–6166.

3 C. Bahou, P. A. Szijj, R. J. Spears, A. Wall, F. Javaid, A. Sattikar, E. A. Love, J. R. Baker and V. Chudasama, *Bioconjug. Chem.*, 2021, **32**, 672–679.

4 M. T. W Lee, A. Maruani, J. R. Baker, S. Caddick and V. Chudasama, *Chem. Sci.*, 2016, **7**, 799–802.

5 F. Bryden, A. Maruani, H. Savoie, V. Chudasama, M. E. B. Smith, S. Caddick and R. W. Boyle, *Bioconjug. Chem.*, 2014, **25**, 611–617.
